# Characterization of Plaque Variants and the Involvement of Quasi-Species in a Population of EV-A71

**DOI:** 10.3390/v12060651

**Published:** 2020-06-17

**Authors:** Madiiha Bibi Mandary, Malihe Masomian, Seng-Kai Ong, Chit Laa Poh

**Affiliations:** 1 Centre for Virus and Vaccine Research, School of Science and Technology, Sunway University, Kuala Lumpur, Selangor 47500, Malaysia; madi.mandary@live.com (M.B.M.); malihem@sunway.edu.my (M.M.); 2 Department of Biological Science, School of Science and Technology, Sunway University, Kuala Lumpur, Selangor 47500, Malaysia; ongsk@sunway.edu.my

**Keywords:** EV-A71, epidemiology, recombination, quasi-species, spontaneous mutations, virulence

## Abstract

Viral plaque morphologies in human cell lines are markers for growth capability and they have been used to assess the viral fitness and selection of attenuated mutants for live-attenuated vaccine development. In this study, we investigate whether the naturally occurring plaque size variation reflects the virulence of the variants of EV-A71. Variants of two different plaque sizes (big and small) from EV-A71 sub-genotype B4 strain 41 were characterized. The plaque variants displayed different in vitro growth kinetics compared to the parental wild type. The plaque variants showed specific mutations being present in each variant strain. The big plaque variants showed four mutations I97L, N104S, S246P and N282D in the VP1 while the small plaque variants showed I97T, N237T and T292A in the VP1. No other mutations were detected in the whole genome of the two variants. The variants showed stable homogenous small plaques and big plaques, respectively, when re-infected in rhabdomyosarcoma (RD) and Vero cells. The parental strain showed faster growth kinetics and had higher viral RNA copy number than both the big and small plaque variants. Homology modelling shows that both plaque variants have differences in the structure of the VP1 protein due to the presence of unique spontaneous mutations found in each plaque variant This study suggests that the EV-A71 sub-genotype B4 strain 41 has at least two variants with different plaque morphologies. These differences were likely due to the presence of spontaneous mutations that are unique to each of the plaque variants. The ability to maintain the respective plaque morphology upon passaging indicates the presence of quasi-species in the parental population.

## 1. Introduction

Enterovirus 71 (EV-A71) belongs to the genus *Enterovirus* within the family *Picornaviridae.* It is a common etiological agent of hand, foot and mouth disease (HFMD) which was first isolated from a young child in the USA in 1969 [1]. It is capable of causing severe neurological diseases which range from encephalitis to acute flaccid paralysis and cardiopulmonary complications which can lead to death [2]. Although EV-A71 has been reported to cause infections in many countries [2,3,4,5], massive outbreaks caused by EV-A71 especially in Asia-Pacific countries such as China, Taiwan and Malaysia are particularly worrisome [6,7,8,9].

Human enteroviruses such as EV-A71 are non-enveloped and contain single-stranded, positive-sense RNA of approximately 7.4 kb [10]. The genome contains two non-coding regions, the 5′-NTR and the 3′-NTR that flank a single open reading frame (ORF) [11]. All four structural proteins (*VP1-VP4*) are encoded by the P1 region of the genome while P2 and P3 encode for the non-structural proteins *2A–2C* and *3A–3D* [10,12]. The complete phylogenetic analysis of EV-A71 based on the structural *VP1* gene identified seven independent lineages: A, B, C, D, E, F and G [13]. Group A consists of the prototype BrCr strain only. Group B is divided into the sub-genotypes B1–B5 which are predominant in Malaysia and Singapore. The C group could be further subdivided into C1–C5 whereby the sub-genotypes C2 and C4 are predominant in China and Vietnam [2,12].

The evolution of EV-A71 has been established to occur naturally and recombination is the fastest way for EV-A71 to evolve, often resulting in novel genomic regions with high diversity when compared to the parental genome [14]. In turn, these new genomic regions may drive a change in the characteristics of the virus such as its virulence [15]. Notably, it was reported that EV-A71 has established its endemicity in the Asia-Pacific region with different circulating strains which include sub-genotypes C1,C2, B3 and B4 [16,17]. Interestingly, another factor that has an impact on the viral pathogenesis in EV-A71 is the occurence of spontaneous mutations. There are numerous reports of single mutations occurring in the genome of EV-A71 that had significantly affected its virulence [18,19,20,21,22]. Multiple changes in the characteristics of EV-A71 caused by these spontaneous mutations could also mediate the evolution of EV-A71. The high divergence of EV-A71 strains circulating in the human population occurred as a consequence of the low fidelity of the 3D RNA-dependent RNA polymerase [15]. The distinctive feature of the viral RNA polymerase used by RNA viruses to induce high mutation rates often results in a swarm of mutant viruses [23]. 

Many different studies attest to the presence of distinct genetic variants arising from wild type viruses. Dengue virus serotype 2 was observed to exhibit mixed plaque phenotypes in LLC-MK cells. Small plaque variants selected from the mixed population were temperature sensitive in LLC-MK cells and became attenuated in suckling mice [24]. Similarly, another study examined the determinants of small plaque phenotype using two different Dengue virus (DENV) vaccine candidate strains: DENV-3 PGMK30FRhL3 and DENV-2 PDK53. It was observed that DENV-3 PGMK30FRhL3 produced small plaques in BHK-21 cells due to its slow in vitro growth rate. However, DENV-2 PDK53 replicated quickly but was unable to evade antiviral responses that constrained its spread; hence it also gave rise to small plaques. It was concluded that at least two molecular mechanisms govern plaque phenotypes in DENV 2 [25]. Likewise, two different plaque variants of Japanese encephalitis virus (JEV) were characterized from the wild-type (WT) whereby the small plaque variant displayed delayed replication kinetics in Vero cells and was resistant to neutralization by monoclonal antibodies [26]. Moreover, Dengue virus serotype 4 (DENV-4) was observed to display small plaque phenotypes with attenuated pathogenicity in mice [27,28,29]. Neuroblastoma cell-adapted yellow fever 17D virus also exhibited the small plaque morphology (SPYF). The virulence properties of the small plaques were distinguishable from non-neuroadapted viruses (YF5.2iv, the infectious clone) whereby it presented decreased average survival time in severe combined immunodeficient (SCID) mice after peripheral inoculation. SPYF also exhibited more efficient growth in peripheral tissues of SCID mice compared to YF5.2iv. This resulted in a quicker accumulation of viral burden but resulted in low-titer viremia at the onset of fatal encephalitis [30,31]. More recently, other viruses were observed to display mixed plaque phenotypes in varying cell lines whereby the plaque variants (big or small) were capable of displaying different levels of virulence in vitro and in vivo [25,32,33,34]. Small plaque variants of chikungunya virus (CHIKV) showed stable homogenous small plaques after four plaque purifications and grew slower with lower titres when compared to the WT virus [35]. Of particular interest, the Zika virus (ZIKV) derived from Asian/American lineage formed small and large plaques with consistent amino acid differences (230^Gln^ for small plaque variants and 230^Arg^ for big plaque variants) in each of the variants. The growth kinetics of the virus clone derived from the large plaque variant was faster than the virus clone derived from the small plaque variant and the amino acid at position 230 in the viral polyprotein is a molecular determinant for plaque morphology, growth characteristics and virulence in mice [34]. In an attempt to characterize the small plaque variants of West Nile Virus (WNV), it was discovered that the SP variant replicated less efficiently than the WT in Vero cells whereas the SP growth was severely restricted at high temperatures in avian cells. Relative to the WT, the SP phenotype exhibited reduced replications and lower infections when administered via different inoculation routes. Analysis of the SP variant identified two amino acid substitutions (prM P54S and NS2A V61A) relative to the WT. These mutations were observed to affect the ability of progeny viruses to infect BHK cells which led to attenuated neurovirulence and neuroinvasiveness of the virus [33].

In this study, the wild-type EV-A71 sub-genotype B4 strain 41 was observed to display small and big plaque variants when growing in Rhabdomyosarcoma (RD) and Vero cells. How these phenotypically different plaque variants of EV-A71 could affect virulence have not been previously explored. We report here how the small-plaque (SP) variant selected from the wild-type EV-A71 strain 41 (Enterovirus 5865/sin/00009) showed a different level of virulence compared to the big plaque (BP) variant when tested in vitro through the analysis growth kinetics, plaque formation, RNA copy number and viral binding. Nucleotide sequencing and 3D homology modelling of the VP1 of the EV-A71 were carried out to investigate how changes in amino acid residues in the big and small plaque variants could affect virulence in vitro.

## 2. Materials and Methods 

### 2.1. Cell Lines 

Human Rhabdomyosarcoma RD (ATCC^®^ CCL136™) cells were grown in Dulbecco’s modified minimal essential medium (DMEM) (Gibco, Carlsbad, CA, USA) supplemented with 10% fetal bovine serum (FBS) (Gibco, Carlsbad, CA, USA), 1% non-essential amino acid and 1% penicillin/streptomycin. The cells were grown at 37 °C supplemented with 5% CO2 until desired confluency was achieved (70–90%).

### 2.2. Serial Passages of Virus

All EV-A71 viruses (Enterovirus 5865/sin/00009) were grown in RD cell monolayers in DMEM supplemented with 2% fetal bovine serum 2 MmL-glutamine, and 1% penicillin/streptomycin at 37 °C. The wild type EV-A71 sub-genotype B4 strain 41 at MOI 1 was added to the monolayer of cells and incubated for 1 h. Then, the viral supernatant was discarded, and the cells were washed with 1× PBS and fresh media (DMEM, 2% FBS and 1% PSA) was added. Next day, the virus was harvested by three cycles of freeze-thawing and the viral supernatant was stored at −80 °C. 

### 2.3. Plaque Assay

Approximately 8 × 10^5^ RD cells/mL were seeded into each well of a 6-well plate and maintained in the complete growth medium. Prior to viral infection, the complete growth medium was removed and washed twice with 1× PBS and then infected with the viral inocula prepared at different serial dilutions of virus stock suspension. After viral adsorption for 1 h at 37 °C, the inocula were removed, the cells were washed twice with 1× PBS and then overlaid with 1 mL plaque medium (containing 1.2% carboxymethylcellulose and 2% FBS). After 72 h of incubation, cells were fixed with 3.7% formaldehyde and stained with 0.5% crystal violet. Plaque forming units were counted.

### 2.4. Isolation of Plaque Variants

Approximately 8 × 10^5^ RD cells/mL were seeded into each well of a 6-well plate and maintained in the complete growth medium. Prior to viral infection, the complete growth medium was removed and washed twice with 1× PBS and then infected with viral inoculum prepared at different serial dilutions (10^−1^ to 10^−10^) of the virus stock suspension. After viral adsorption for 1 h at 37 °C, the inocula were removed, the cells were washed twice with 1× PBS and then overlaid with 2 mL of DMEM with 2% FBS and 1%PSA containing 1.5% of agarose as the overlaying media (Hydragene, Xiamen, China). The overlay was allowed to solidify before incubation at 37 °C for 3 days. After 3 days, the plaques were marked with a pen under the microscope. Using a sterile glass pipette tip, an agarose plug was removed directly over the plaque. Each plaque that was picked up was added to separate wells of a 24-well tissue culture plate seeded with 4 × 10^5^ cells per well in 500 mL of fresh DMEM media. The plates were incubated at 37 °C overnight before harvesting the virus plaque variants.

### 2.5. Re-Infection of the Cells by EV-A71/BP and EV-A71/SP Plaque Variants and the Determination of Their Plaque Sizes

Isolated plaques were used to infect RD and Vero cells in 6-well plates at different viral dilutions. After viral adsorption for 1 h at 37 °C, the inocula were removed, the cells were washed twice and then overlaid with 2 mL of DMEM with 2% FBS and 1%PSA containing 1.5% of agarose overlay (Hydragene, Xiamen, China). The overlay was allowed to solidify before incubation at 37 °C for 3 days. After 72 h of incubation, the cells were stained for 6 h with crystal violet stain (Sigma, St. Louis, MO, USA) before plaques were observed. Plaques were examined and images of the whole well were captured with an Immunospot^®^ S6 VERSA Analyzer (Cellular Technology Limited, Shaker Heights, OH, USA). Diameters of plaque variants in monolayers of RD and Vero cells were determined. For each plaque variant, at least 400 plaques were measured using the DS-L3 viewer with the Nikon’s NIS-Elements software and analysed by Graph pad prism 7.04.

### 2.6. Growth Kinetics 

RD and Vero cells at a density of 8 × 10^5^ cells/mL were seeded within the wells of multiple 6-well plates. When the desired confluency was achieved, the cells were washed with 1× PBS and infected with each viral plaque variant in triplicates at a MOI of 0.1 and incubated for 60 min at 37 °C. After this, the virus inoculum was discarded, and the RD and Vero cells were washed with 1× PBS before being replaced with reduced growth media (DMEM supplemented with 2% FBS and 1% PSA). The virus supernatants were harvested at 12 h, 24 h, 48 h and 72 h post-infection (h.p.i) and were subsequently kept at −80 °C for downstream processes such as the determination of RNA replication expressed as viral copy number using RT-qPCR and infectivity in PFU/mL using plaque assay.

### 2.7. Rates of RNA Replication (RT-qPCR)

Primers targeting the VP1 sequence of the EV-A71 were adopted from a previous study [36]. The forward primer employed was 5′-GAGCTCTATAGGAGATAGTGTGAGTAGGG-3′, the reverse primer was 5′-ATGACTGCTCACCTGCGTGTT-3′ and the TaqMan probe used was 5′6-FAM-ACTTACCCA/ZEN/GGCCCTGCCAGCTCC-lowa Black FQ-3′. The viral RNA samples were extracted using QIAamp Viral RNA mini kit (QIAGEN, Hilden, Germany) according to the manufacturer’s instructions. The RT TaqMan real-time PCR assay was performed with the C1000 Touch Thermal Cycler CFX96 Real-time System (BIORAD, Hercules, CA, USA) using TaqMan^®^ Fast virus 1-step master mix (ThermoFisher Scientific, Waltham, MA, USA) with cDNA synthesis by reverse transcription for 5 min at 50 °C and subsequently amplified for 40 cycles at 95 °C for 3 s, 60 °C for 30 s. Three independent experiments were conducted for each sample. The threshold cycle value (C_q_) was determined using default threshold settings, and the mean C_q_ was calculated from the PCR runs in triplicates. A standard graph was plotted based on a series of standard solutions containing the EV-A71 RNA samples from 10^−1^ to 10^−6^.

### 2.8. Amplification of EV-A71 RNA to cDNA 

Viral RNA was isolated from the EV-A71 plaque isolate using QIAmp^®^ Viral RNA Mini Spin Kit (Qiagen, Hilden, Germany). The kit was used according to the manufacturer’s instructions. The purified RNA was reverse transcribed into single-stranded DNA by using the SuperScriptTM IV First Strand Synthesis System (ThermoFisher Scientific, Waltham, MA, USA). Each cDNA synthesis mixture contained 10× annealing buffer, 25 mM MgCl2, SuperScript^®^ IV RT and the viral RNA as template. Viral double-stranded cDNA was amplified with LongAmp^®^ Taq DNA Polymerase (NEB, Ipswich, MA, USA) using designed primer sets. Agarose gel (0.5%) was prepared in 1× Tris-acetate-EDTA (TAE) buffer. SYBR™ Safe DNA Gel Stain (Invitrogen, Carlsbad, CA, USA) was added to the agarose gel at final concentration of 0.5 µg/mL. Gel Loading Dye Purple (6×) (NEB, Ipswich, MA, USA) was mixed with the DNA samples before loading into the wells of the gel. The GeneRuler 1 kb DNA Ladder (ThermoFisher Scientific, Waltham, MA, USA) was used to determine the size of the DNA (dsDNA) fragments in the gel. PCR products were verified as being successfully amplified and they appeared as bright distinct bands on the agarose gel following electrophoresis at 95 V for 90 min.

### 2.9. Genome Sequencing

After the PCR products were confirmed by agarose gel electrophoresis, the PCR products were sequenced using designed primers. Raw sequencing data were assembled to reconstruct the complete EV-A71 genome using the Geneious Software (Biomatters, Auckland, New Zealand) and Clustal Omega (Conway Institute UCD, Dublin, Ireland) See Supplementary Table S1: Primers used for the whole genome sequencing of EV-A71.

### 2.10. Homology Modelling

The VP1 protein of the EV-A71 parental strain, the big and small plaque variants, were modelled using YASARA Structure (“Yet Another Scientific Artificial Reality Application”, http://www.yasara.com) Software. The VP1 three-dimensional (3D) crystal structures of four human enterovirus A71 (PDB ID: 4AED, 6I2K, 4CDQ and 3VBF) with sequence identity of more than 95% were used as the templates by homology search from The National Center for Biotechnology Information (NCBI) and Protein data bank (PDB) database. The predicted model was subjected to energy minimization by YASARA software. Then, the model was validated using pdbsum (http://www.ebi.ac.uk/thornton-srv/databases/pdbsum/Generate.html), Verify 3D (http://servicesn.mbi.ucla.edu/Verify3D/) and ERRAT (http://servicesn.mbi.ucla.edu/ERRAT/) programs. (Supplementary Table S2: PSI-BLAST of the VP1 protein sequence of the EV-A71 parental strain.)

### 2.11. Viral Binding Assay 

In order to examine the virus binding ability by ELISA, 2.0 × 10^4^ cells/mL of RD and Vero cells were seeded into each well of a 96-well plate and maintained in complete growth media for 1 day. Prior to viral infection, the complete growth media was removed, and the cells were washed with 1× PBS and then infected with viral inocula of plaque isolates at MOI of 10 and incubated for 1 h at 4 °C. Unbound viruses were washed away with cold 1× PBS. Cells were then fixed with 80% acetone and stained with monoclonal Enterovirus 71 VP1 antibody (GeneTex, Irvine, CA, USA) at 1:2000 dilution in 1%BSA for 1 h. This was followed by washing with PBS-T (0.05% Tween 20 in PBS) and staining with mouse anti-goat IgG HRP (Santa Cruz Biotechnology, Dallas, TX, USA) at 1:2000 dilution in 1% BSA for 1 h. Once washed with PBS-T, KPL TrueBlue™ Peroxidase Substrate (50 µL) (Sera Care Life Science, Milford, MA, USA) was added to all infected cells within each well and the absorbance was measured using Infinite^®^ 200 PRO (Tecan, Männedorf, Switzerland) at 650 nm.

## 3. Results

### 3.1. Isolation of Big-Plaque and Small-Plaque Variants of EV-A71

**Figure 1:**
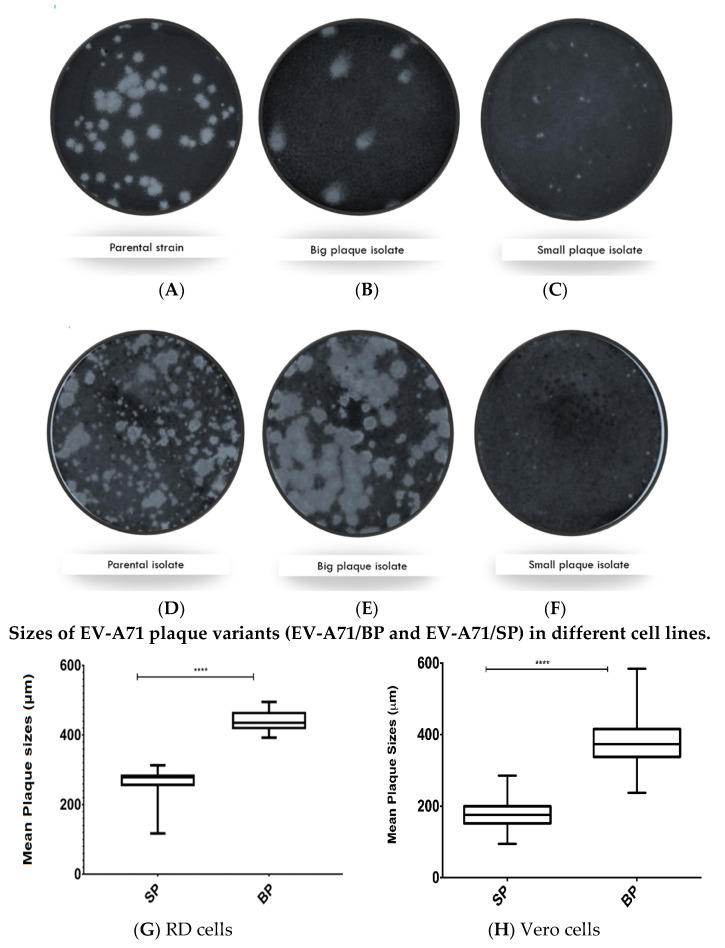
Plaque morphology of the (**A**) Parental WT strain, (**B**) big plaque variant (EV-A71/BP), and (**C**) the small plaque (EV-A71/SP) variant cultured in RD cells. Plaque morphology of the (**D**) Parental WT strain, (**E**) the big plaque (EV-A71/BP) variant, and (**F**) the small plaque (EV-A71/SP) variant when cultured in Vero cells. Cells within the wells of a 6-well plate were infected with the wild type EV-A71 (5865/sin/000009) or the EV-A71/BP and EV-A71/SP. The plaques were visualised 72 hpi (hours post infection) after staining with crystal violet. Plaques were examined and images of the whole well were captured with an Immunospot^®^ S6 VERSA Analyzer (Cellular Technology Limited, Shakers Heights, OH, USA). Diameters of plaque variants in monolayers of RD and Vero cells were determined. For each plaque variant, at least 400 plaques were measured using the DS-L3 viewer with the Nikon’s NIS-Elements software and analysed by Graph pad prism 7.04. The mean plaque size of all isolates exhibiting small plaque morphology were compared against the mean plaque size of all isolates exhibiting big plaque morphology in (**G**) RD and (**H**) Vero cells. Statistical analysis, unpaired *t*-test, **** *p* < 0.0001. See Supplementary Table S3: Unpaired *t*-test analysis of the EV-A71/BP and the EV-A71/SP plaque variant using GraphPad Prism.

In both RD and Vero cells, the wild type (EV-A71/WT) sub-genotype B4 strain 41 (5865/sin/000009) population exhibited mixed-plaque phenotypes represented by both big and small plaque variants designated as EV-A71/BP and EV-A71/SP, respectively (Figure 1A,D). Isolation of these individual plaque variants led to the observation that EV-A71/BP variants displayed uniformly big plaque phenotype in both RD and Vero cells (Figure 1B,E). In contrast, the EV-A71/SP variants produced very tiny, pin-sized plaques compared to the larger plaque variants observed in the EV-A71/BP populations in both cell lines (Figure 1C,F). Subsequently, the diameter of 400 plaques from the EV-A71/BP and the EV-A71/SP populations were measured. The EV-A71/SP observed in RD cells were significantly smaller (mean: 0.293 ± 0.002 mm) than those of the EV-A71/BP (0.519 ± 0.004 mm; *p* < 0.01, *t*-test) (Figure 1G). Furthermore, EV-A71/SP virus variant in Vero cells (mean: 0.177 ± 0.002 mm) were significantly smaller than those of the EV-A71/BP variant (0.380 ± 0.003 mm; *p* < 0.01, *t*-test) (Figure 1H). The results showed that there was a significant size difference amongst all the isolates exhibiting the small plaque morphology when compared to all the isolates exhibiting the big plaque morphology.

### 3.2. In Vitro Assessment of the Big and Small Plaque Variants

**Figure 2:**
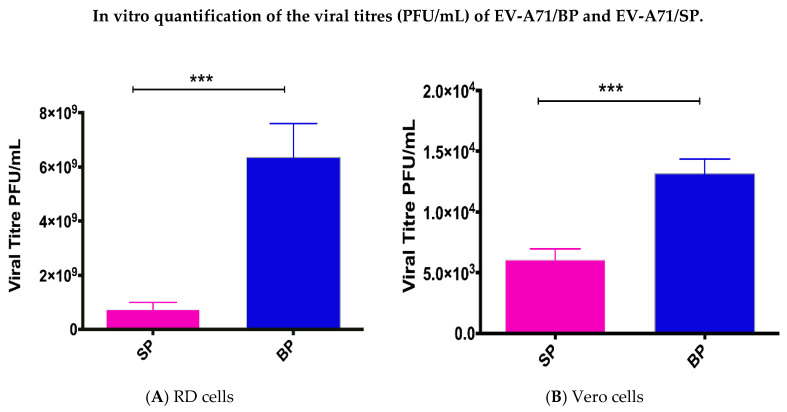
Comparison of the plaque forming ability between the small plaque variant against the big plaque variant was quantified by plaque assays. Quantification of plaque forming ability in PFU/mL was compared between the small and big plaque variants in RD cells (**A**) and (**B**) Vero cells. *p*-values were calculated using two-tailed *t*-test on Graph Pad; *** *p* < 0.001.

The ability of the two EV-A71 variants to form plaques of different morphologies was evaluated in both RD and Vero cells (Figure 2A,B). The big plaque variants produced the highest number of plaques (5.62 × 10^9^ ± 6.40 × 10^8^ PFU/mL) in RD cells but had lower viral titres in Vero cells (1.32 × 10^4^ ± 603.5 PFU/mL) (Table 1). Lesser number of viral plaques (one log less) were observed for the small plaque variants in RD (6.35 × 10^8^ ± 6.25 × 10^8^ PFU/mL) and Vero cells (6.05 × 10^3^ ± 455.5 PFU/mL). Since the ability to form plaques was reduced for the small plaque variant, viral growth was confirmed to be lower. Lower viral growth indicated lower virulence of the small plaque variant in RD and Vero cells.

### 3.3. Rates of RNA Replication and Infectivity of EV-A71/BP and EV-A71/SP Plaque Variants

**Figure 3:**
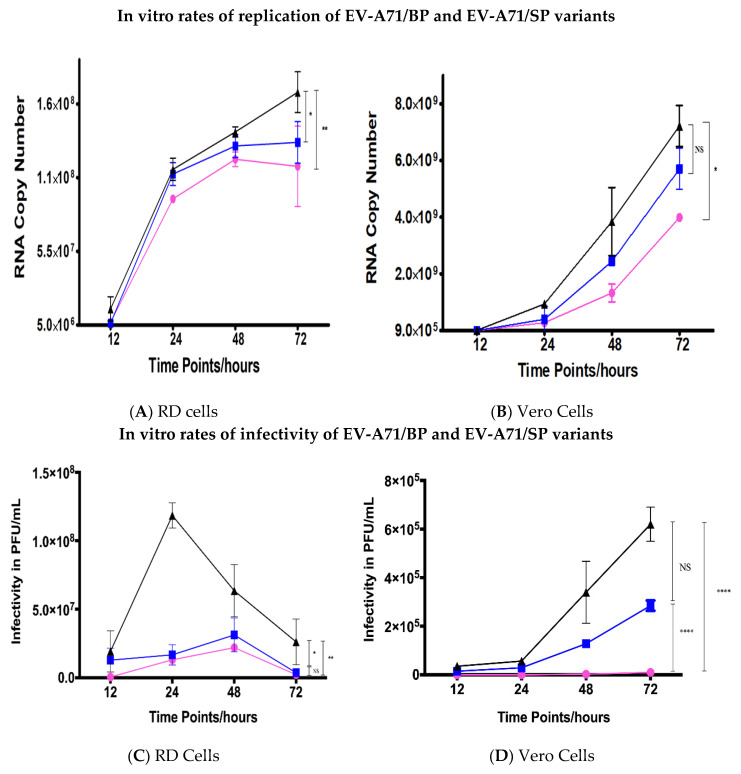
Comparison of rates of RNA replication of wild-type (WT), big plaque (BP) and small plaque (SP) variants in vitro in (**A**) RD and (**B**) Vero cells. RD and Vero cells in wells within the 6-well plates were infected with EV-A71/WT, EV-A71/BP and EV-A71/SP variants in triplicates at a MOI of 0.1 PFU/mL. Viral supernatants were harvested from the medium at 12, 24, 48 and 72 hpi and subjected to qRT-PCR in triplicates to obtain the rates of RNA replication in viral copy number. The EV-A71/WT is shown in black, EV-A71/BP is depicted in blue and EV-A71/SP is coloured magenta. Infectivity of the EV-A71 plaque variants (EV-A71/BP and EV-A71/SP) and the parental strain (EV-A71/WT) in (**C**) RD cells and (**D**) Vero cells. The WT strain is shown in black while the big plaque variant (EV-A71/BP) is displayed in blue and the small plaque variant (EV-A71/SP) is in magenta. Statistical analysis used was unpaired *t*-test; * *p* < 0.05, ** *p* < 0.01, **** *p* < 0.0001 and NS indicates no significance.

Both EV-A71 plaque variants had lower RNA replication rates in RD and Vero cells when compared to the wild type (EV-A71/WT) (Figure 3A,B). Overall, the EV-A71/WT (coloured in black) was observed to display significantly better rates of RNA replication when compared to the EV-A71/BP (coloured in blue) and EV-A71/SP variants (coloured in magenta). Meanwhile, the EV-A71/SP had the lowest rate of RNA replication when compared to both the EV-A71/WT and the EV-A71/BP variant. A sharp increase in the overall RNA rates of replication was observed from 12 hpi onwards in RD cells for the EV-A71/WT and the two plaque variants (Figure 3A). Subsequently, the rate of replication was observed to plateau at 72 hpi for the EV-A71/BP (1.29 × 10^8^ viral copy number) while the replication of the EV-A71/SP (1.127 × 10^8^ viral copy number) variant was observed to decline at 72 hpi. However, the EV-A71/WT continued to increase to 1.67 × 10^8^ viral copy number at 72 hpi. Thus, the most significant difference was observed to occur between the overall viral RNA copy number of the EV-A71/SP variant and the EV-A71/WT (*p* < 0.01, one-way ANOVA).

On the other hand, replication of the EV-A71 in Vero cells was observed to lag by 12 h when compared to the RD cells whereby the rate of replication was only shown to rise sharply after 24 hpi (Figure 3B). There was a slight difference in the RNA copy number between the EV-A71/WT and the two plaque variants at 24 hpi. The difference became greater as the infection progressed to 24, 48 and 72 hpi. The significant difference in RNA copy number between the EV-A71/WT and the EV-A71/SP was 3.225 × 10^9^ viral copy number (*p* < 0.05, one-way ANOVA). Additionally, it was intriguing to observe that the peak RNA copy number in Vero cells for the EV-A71/WT, EV-A71/BP and EV-A71/SP variants was one log higher than the peak RNA copy number of the EV-A71/WT and the EV-A71 plaque variants in RD cells. Overall, these results suggested that the replication kinetics of the wild type parental strain (EV-A71/WT) was significantly higher than the two EV-A71 plaque variants independent of the host cell lines used in this study. 

To further clarify the viral replication rates of the two EV-A71 plaque variants with respect to the wild type, we next compared their kinetics of infectivity at different time points to gain an understanding of the rate of infectivity. The parental strain displayed significantly higher levels of infectivity when compared to both the EV-A71/BP and EV-A71/SP variants (Figure 3C,D). The EV-A71/BP variant displayed higher levels of infectivity compared to the small plaque variant which was consistent with the general pattern observed in RNA replication in the RD cells. However, no significant difference in the levels of infectivity was observed between the EV-A71/BP and EV-A71/SP variant in RD cells. The infectivity of the EV-A71/WT in RD cells peaked at 24 hpi (1.185 × 10^8^ PFU/mL) while the EV-A71/BP and EV-A71/SP variants lagged by 24 h and peaked at 48 hpi (3.125 × 10^7^ PFU/mL and 2.2 × 10^7^ PFU/mL respectively) before decreasing to substantially lower titres (Figure 3C). 

In Vero cells, the rates of infectivity of both plaque variants (EV-A71/BP and EV-A71/SP) were significantly lower than the EV-A71/WT. The maximum infectivity was 6.2 × 10^5^ PFU/mL at 72 hpi for the EV-A71/WT but the EV-A71/BP variant showed much lower titre than the EV-A71/WT with 2.85 × 10^5^ PFU/mL at 72hpi (Figure 3D). The infectivity of the EV-A71/SP variant in Vero cells was undetectable at 12 and 24 hpi and very low titres were detected throughout infection from 48 hpi onwards (1 × 10^4^ PFU/mL at 72 hpi). At 72 hpi, the difference in titres between the EV-A71/WT and the EV-A71/SP variant was one log difference (6.2 × 10^5^ PFU/mL when compared to 1 × 10^4^ PFU/mL). The growth kinetics of the EV-A71/WT in comparison with both plaque variants, EV-A71/BP and EV-A71/SP, in terms of the rate of RNA replication and infectivity showed that the small plaque variant (EV-A71/SP) had lower kinetics of growth than the EV-A71/WT and the EV-A71/BP variants at all time–points of sampling in both RD and Vero cells.

### 3.4. Sequence Comparison of Small-Plaque and Big-Plaque Variants to the WT

**Figure 4:**
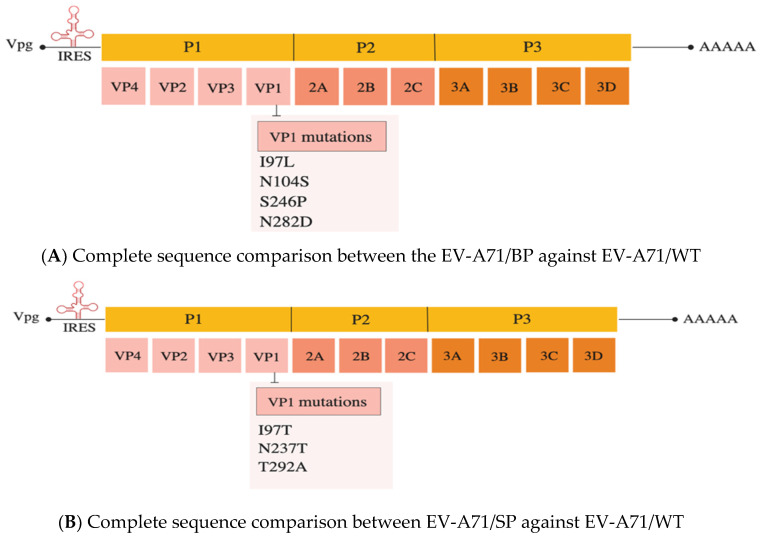
(**A**) The VP1 amino acid sequence of the WT (parental EV-A71 strain) against EV-A71/BP. Significant non-synonymous mutations are: I97L, N104S, S246P and N282D. (**B**) The VP1 amino acid sequence homology of the WT (parental EV-A71 strain) and the EV-A71/SP was performed using Clustal Omega. Significant non-synonymous mutations are: I97T, N237T and T292A in the VP1 of the EV-A71/SP.

The differences in plaque morphology, growth kinetics and in vitro infectivity observed could be due to the presence of mutations present in the genomes of the EV-A71/BP and EV-A71/SP variants. To confirm whether there are mutations present in the EV-A71/BP and EV-A71/SP variants, the entire genome of each EV-A71 plaque variant was amplified and sequenced via Sanger sequencing and compared with the full genome of the sequenced EV-A71/WT. (Supplementary Figure S1: Agarose gel electrophoresis of the full-length genomic cDNA of EV-A71/WT and Supplementary Figure S2: Raw genome sequencing data of EV-A71/BP and EV-A71/SP). Comparison of the EV-A71/WT sequence against the genome of the four EV-A71/BP variants revealed the presence of the same four non-synonymous mutations present in the VP1 region, namely: I97L, N104S, S246P and N282D (Figure 4A). Likewise, analysis of the four EV-A71/SP variants showed three non-synonymous mutations present in the VP1 region, namely: I97T, N237T and T292A (Figure 4B). These results suggest that any of these non-synonymous mutations present in the EV-71/BP and EV-71/SP variants could account for the differences in plaque morphologies and infectivity observed in vitro in RD and Vero cells. See Supplementary Table S4: Amino acid changes observed on the genomes of EV-A71/BP and EV-A71/SP variants.

### 3.5. Analysis of Amino Acid Mutations in the VP1 of the EV-A71 Plaque Variants

To understand how the non-synonymous mutations, present in the big and small plaque variants could affect the interaction of the virus with the RD or Vero cells, the contribution of each mutation towards protein folding and stability was analyzed by 3D modelling. The modelling process involved several steps, such as target template selection and model building. The accuracy of the models increased along with the increase in the number of known, close and high-resolution protein structures. Since the VP1 three-dimensional (3D) structure of the EV-A71 sub-genotype B4 strain 41 was not found in the protein data bank, it was necessary to model the EV-A71 parental strain prior to using it as a reference for analysis of theEV-A71 plaque variants. The model was built using YASARA Structure (17.4.17) software. (Supplementary Figure S3: The 3D structure of the VP1 protein of the EV-A71/WT superposed against the EV-A71/BP and EV-A71/SP variants.)

**Figure 5:**
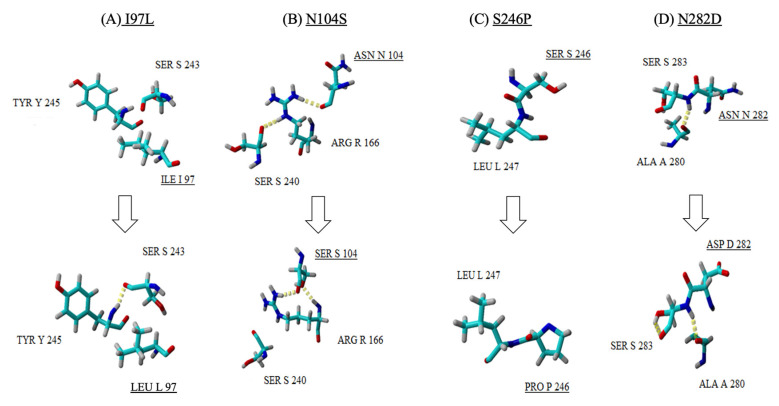
A close up view of the four mutations (**A**–**D**) observed in the EV-A71/BP structure (bottom row) when compared with the EV-A71/WT (top row). Amino acid substitutions are underlined, and the hydrogen bonds are observed as yellow dotted lines.

**Figure 6:**
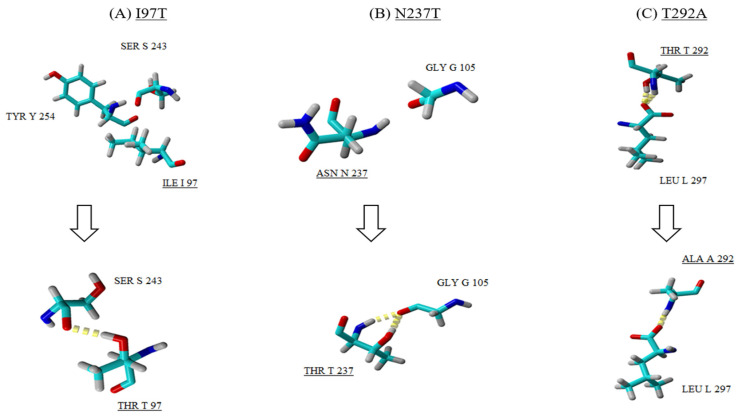
A close up view of the three mutations (**A**–**C**) observed in the EV-A71/SP structure (bottom row) when compared with the EV-A71/WT (top row). Amino acid substitutions are underlined and the hydrogen bonds are observed as yellow dotted lines.

The big plaque variant, EV-A71/BP, was observed to carry four mutations (I97L, N104S, S242P and N282D) in its genome which caused differences in the 3D structure of the VP1 protein compared to the wild-type strain, EV-A71/WT. The mutation found at position 97 (EV-A71/SP: I97T and EV-A71/BP: I97L) is of significant interest as it is found in the BC loop of the VP1 protein and appears to be a recurrent and favoured spot for mutation in all EV-A71/BP and EV-A71/SP variants. In the EV-A71/WT, isoleucine (I) is found at position 97, but mutation to either threonine (T) or leucine (L) in the EV-A7/BP and EV-A71/SP variants, respectively, caused the formation of new hydrogen bonds (Figure 5A and Figure 6A). For instance, changing isoleucine (I) to threonine caused the latter to form a single hydrogen interaction with serine (S) at position 243. In the big plaque variant, a hydrogen bond was formed between serine (S) at position 243 and tyrosine (Y) at position 245. Moreover, despite the change in amino acid at position 104 (asparagine to serine) in the EV-A71/BP variant, no change in the characteristics of the amino acid was observed as both asparagine and serine belong to the same family of amino acids with polar uncharged side chains (Figure 5B). Interestingly in the EV-A71/WT, Asn^104^ (asparagine) initially formed a single hydrogen bond with Arg^166^ (arginine) which in turn interacted with Ser^240^ (serine). However, when asparagine (N) was substituted with Ser^104^ (serine) in the EV-A71/BP variant, it formed two hydrogen bonds with the Arg^166^ (arginine) and no interactions were observed with Ser^240^ (serine). At position 246 of the EV-A71/BP, the mutation induced a change in the characteristics of the amino acid such that a polar uncharged amino acid (serine) was replaced with the proline residue (Figure 5C). Of significant interest is the position at 282 near the N-terminus of the VP1 protein, whereby the polar uncharged asparagine (N) was replaced by aspartic acid (D) which has a negatively charged side chain. When asparagine (N) was found at position 282 in the EV-A71/WT, the neighbouring amino acid residue, Ser^283,^ interacted via a single hydrogen bond with the Ala^280^. However, when mutation induced a change to aspartic acid (Asp^282^), the latter gained an additional hydrogen bond interaction with Ala^280^ (Figure 5D). 

All three mutations (I97T, N237T and T292A) identified in the VP1 of the EV-A71/SP variants contributed to the difference observed in the 3D structure of the VP1 protein when compared to the EV-A71/WT. Besides the mutation I97T, two other mutations (N237T and T292A) were discovered to play a role in affecting the folding of the VP1 protein of the EV-A71/SP variant. For instance, mutation at position 237 caused asparagine (Asn^237^) to change to threonine (T). As a result, this induced the formation of two hydrogen bonds with neighbouring residue glycine (Gly^105^) found in the pocket factor of the VP1 protein (Figure 6B). The last amino acid substitution was observed at position 292 (threonine to alanine) which was present on the surface of the capsid (Figure 6C). In the EV-A71/WT, threonine at position 292 (Thr^292^) interacted with leucine (Leu^297^) via two hydrogen bonds. However, mutation of Thr^292^ to Ala^292^ resulted in the loss of one hydrogen bond in the interaction with Leu^297^. In addition, substitution of threonine, which is a polar uncharged amino acid, with alanine increased the hydrophobicity of the amino acid in the EV-A71/SP. 

It can be observed that these mutations and their characteristics contributed to the structural differences in the VP1 protein of the EV-A71/BP and EV-A71/SP variants by interacting with neighbouring amino acid residues.

### 3.6. Impact of Spontaneous Mutations on VP1 Protein Folding

**Figure 7:**
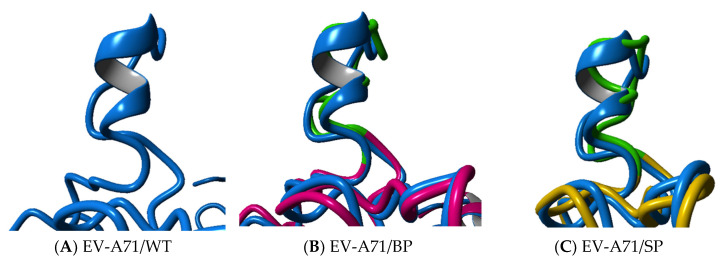
Superposition analysis of the GH loop of EV-A71/BP and EV-A71/SP variants in comparison with the (**A**) EV-A71/WT (blue). (**B**) The EV-A71/BP is depicted in magenta and (**C**) EV-A71/SP is coloured yellow. The GH loop of the plaque variants EV-A71/BP and EV-A71/SP is highlighted in green.

While the overall folding conformation of the VP1 proteins of EV-A71/BP and EV-A71/SP variants appear to remain mostly unaffected, it was observed that there are significant structural variations between the wild type structure and the EV-A71 plaque variants (Table 2). The secondary structure analysis of the EV-A71/WT and the two plaque variants showed that the VP1 protein of the EV-A71/WT contained nine helical structures and several intermediate loops. However, a loss of α-helix was observed in the EV-A71/BP variant, which contained seven helices while the EV-A71/SP had six helices only. The loss of two α-helices seen in the EV-A71/BP was also accompanied by the increase in turn and 3_10_ helices in the structure of the EV-A71/BP variant. On the other hand, the loss of two α-helix structures in the EV-A71/SP was associated with a rise in turn and coil structures (Table 2). Upon closer examination, it was observed that the new mutations induced changes in the cytosolic GH loop (residue 202–222) of the VP1 of the EV-A71/BP and EV-A71/SP variants in contrast with the EV-A71/WT. The helical GH loop in the EV-A71/WT was changed to a coiled structure in the EV-A71/SP variant. However, the EV-A71/BP variant only exhibited partial loss of the helical conformation and displayed within its GH loop a 3_10_ helical structure instead (Figure 7).

Superposition of the EV-A71/BP and EV-A71/SP variants against the EV-A71/WT resulted in calculated root-mean-square deviation (RMSD) values of 1.76Å and 1.35Å, respectively. Due to the presence of four mutations in the EV-A71/BP variant, it can be inferred that the EV-A71/BP variant had more conformational changes and displayed greater structural variations than the EV-A71/SP variants. The EV-A71 plaque variants exhibiting structural variations are also supported by the decrease in hydrogen bonding interactions. For instance, the reference structure EV-A71/WT displays 108 hydrogen bonding interactions. However, the EV-A71/BP variant displays 104 interactions only while the EV-A71/SP variant shows the loss of a single hydrogen bonding interaction from 108 to 107 compared to the EV-A71/WT. The decrease in the hydrogen bonding interactions of the EV-A71/BP together with the increased RMSD value confirms the greater degree of structural variations observed in the EV-A71/BP variant. Together these findings emphasize the important role of the novel spontaneous mutations on the overall conformation of the VP1 structure of the two EV-A71 plaque variants: EV-A71/BP and EV-A71/SP. These findings also suggest the possible loss of function of certain part of the VP1, likely due to the changed conformation of the overall VP1 structure.

### 3.7. Viral Binding Ability

Analysis of the VP1 structure via structural modelling showed that the mutations uncovered through Sanger sequencing were responsible for the alterations of the VP1. The superimposition of the EV-A71/BP and EV-A71/SP variants against the EV-A71/WT showed structural changes in the GH loop. The GH loop region of the VP1 capsid has been previously established to play a crucial role in exhibiting interaction between EV-A71 and its cellular receptor(s). It is therefore likely that the mutations affecting the structure of the GH loop are involved in influencing the virus binding ability of the EV-A71 in RD and Vero cells.

The results demonstrated that the viral binding of the EV-A71 plaque variants (EV-A71/BP and EV-A71/SP) was significantly reduced in both cell lines. In both host cells, the EV-A71/WT exhibited the highest binding ability compared to the EV-A71/BP and EV-A71/SP variants. Statistical analysis used was one-way ANOVA, *p* < 0.05.

**Figure 8:**
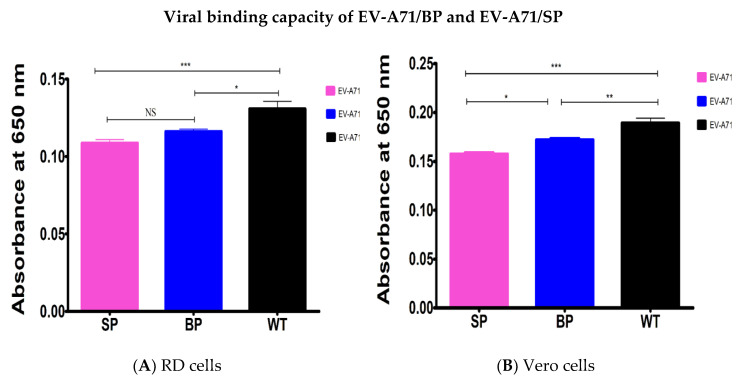
Contribution of mutations of the EV-A71/BP and EV-A71/SP variants to the virus binding ability was determined by ELISA in (**A**) RD cells and (**B**) Vero cells. The EV-A71/WT is displayed as the reference. Data represent the averages from at least three independent experiments ±SD, Statistical analysis used was one-way ANOVA, * *p* < 0.5, ** *p* < 0.01, *** *p* < 0.001 and NS indicates no significance.

In RD cells, the EV-A71/BP demonstrated a significant loss in binding ability of 11% compared to the EV-A71/WT. However, the loss in binding capacity of the EV-A71/SP was significantly higher at 17% compared to the EV-A71/WT (Figure 8A). When Vero cells were used, the binding capacity of the EV-A71/BP variant was higher than the EV-A71/SP variant by 8%. However, the loss in binding ability of the EV-A71/BP compared to the EV-A71/WT was only at 10%. Much like that being observed in RD cells, there was a 17% loss in binding capabilities for the EV-A71/SP in contrast with the EV-A71/WT (Figure 8B).

## 4. Discussion

EV-A71 continues to be a significant public health threat in Asia. Being one of the main etiological agents of HFMD, EV-A71 has the capability to cause severe neurological complications such as acute flaccid paralysis, neurological pulmonary oedema and even deaths, mainly in children [37]. Hence, it is vital to understand the underlying cause of attenuation as well as the selection process of virulent strains.

The evolution of EV-A71 strains within a viral population could arise from recombination and spontaneous mutations [14]. The observation of the viral variants exhibiting big and small plaque morphologies clearly indicate the existence of viral variants carrying different mutations in their genomes. In this study, we showed that the wild-type population of EV-A71 sub-genotype B4 strain 41 contained at least two genotypes: big and small plaque variants which exhibited two different morphologies in terms of their plaque sizes. The persistence of the mutations in the genomes of the big and small plaque variants confirmed the presence of at least two quasi-species in the EV-A71 wild type population. Thus, this study shows for the first time the two EV-A71 plaque variants exhibiting genotypic differences and their in vitro virulence in RD and Vero cells.

The morphology of the EV-A71/SP and EV-A71/BP variants was reflected in the determination of plaque sizes following the selection of the EV-A71 variants. RD cells significantly demonstrated clearer resolution of plaque sizes for both variants when compared to the Vero cells. This difference in plaque sizes across diverse cell lines was demonstrated in a previous study which showed that RD cells were more sensitive and receptive to EV-A71 infections [38]. This could explain why, in general, the plaques observed in the monolayer of RD cells were much bigger and well defined than those observed in the Vero cells. However, the mean measurements of diameters of the two selected plaques (big and small) in the Box and Whisker plot did not overlap which indicated the successful isolation of two distinct populations. Recently, a study characterised three isolates of Zika Virus (ZIKV) which exhibited three plaque sizes, namely: big, small and medium plaques [39]. Additionally, an African horse sickness virus (AFHS) population was characterised and was also shown to harbour three distinct plaque size variants [40]. As such, it is very likely that the EV-A71/WT population could be harbouring a variant of medium plaque size. which would be expected to exhibit a mean diameter lying between the non-overlapping diameter sizes of the EV-A71/BP and EV-A71/SP recorded in this study. 

In the current investigation, it was shown that both variants displayed different growth kinetics in the RD and Vero cells. The susceptible nature of the RD cells to EV-A71 infection [38] coupled with the time frame of the infection (72 h) gave the EV-A71 plaque variants an advantage in infecting RD cells and propagating to a greater extent. It caused greater cell death (CPE) in a shorter interval of time when compared to the EV-A71 plaque variants in Vero cells. The longer adaptation to Vero cells could have led to the disparity in the growth kinetics and viral titres observed between RD and Vero cells. It is likely that full cytopathic effects (CPE) have occurred in the RD cells significantly before Vero cells. Plaque sizes have previously been presented as an effective indicator of virulence [41]. More specifically within the picornaviruses, a correlation was found to exist between virulence and plaque sizes whereby large plaque variants were found to be more virulent than small plaque variants [42]. Hence, it is postulated that the homogeneous populations of both the EV-A71/BP and EV-A71/SP variants would also exhibit differences in their individual replicative and infective abilities. 

The most striking element of the growth kinetics study was that the EV-A71/WT displayed higher growth kinetics when compared to the EV-A71/BP and EV-A71/SP variants. It was suspected that the EV-A71/WT population exhibited better viral fitness which might have accounted for the elevated rates of replication and infectivity in both RD and Vero cells. As such, increased viral fitness of an RNA virus such as EV-A71 has been previously shown to be related to the phenomenon of quasi-species [43]. The phenomenon of quasi-species refers to an RNA population consisting of an ensemble of genetically related genomes often constituting a broad spectrum of mutants [44,45]. Mutants with varying levels of infectivity were generated from the wild-type population through the high mutation rates brought about by the low replication fidelity of the RNA polymerase [46]. Recently, Huang et al. (2017) provided the first indication of the occurrence of quasi-species of EV-A71 whereby the parental population comprised different strains which coexisted, and they displayed high viral growth and fitness in neuronal cells [43]. Therefore, it would be plausible that a similar scenario was at play in this study whereby the EV-A71/WT sub-genotype B4 strain 41 harboured plaque variants which exhibited cooperative behaviour for survival. A more in-depth analysis would be required to verify the existence of how many quasi-species were present in the EV-A71 sub-genotype B4 strain 41 population and the extent of their cooperative behaviour in both RD and Vero cells.

The in vitro rates of RNA replication also confirmed that the EV-A71/SP variant displayed the lowest ability to infect and replicate in RD and Vero cells at all time points of sampling. This could be due to the EV-A71/SP variant itself, whereby it might have exhibited a defective nature of viral binding and cell-to-cell spread which would explain the lower replication kinetics and rate of infectivity recorded in this study. Similar cases of defective small plaques were previously reported for other RNA viruses such as West Nile virus (WNV) and Chikungunya (CHIKV), whereby it was postulated that the small plaque variant existed as a minor fraction of the quasi-species population which exhibited lower viral fitness when isolated and characterized in vitro and in vivo [33,35]. Additionally, the sudden rise at 24 hpi of the viral copy number in Vero cells could be associated with the slow growth rate of the EV-A71 virus in the latter. Since the original isolation of all the plaque variants in this study was initially performed in a monolayer of RD cells followed by their subsequent infection in Vero cells, it is suspected that a degree of in vitro adaptation occurred in the EV-A71/WT and the two plaque variants in Vero cells before the RNA copy number peaked after 24 hpi. Similar observations were made in another study where alternating growth and isolation of ZIKV plaque variants in mosquito cell lines (C6/36 cells) and their subsequent infections in the mammalian cell line (Vero cells) caused the ZIKV isolates to undergo a period of in vitro adaptation before peak replicative and infective titres were achieved [39].

Interestingly, it is suspected that the disparity observed between RD and Vero cells reflected the physiological differences of the host cells [39]. A previous study showed that RD cells exhibited higher levels of the SCARB-2 cellular receptor [47]. Indeed, it is a well-known fact that cell lines of human origins such as RD cells are rich in expressions of receptors that enable EV-A71 to enter cells [48,49]. However, contrary to RD cells which utilise SCARB2 as a major receptor for EV-A71 infection, Vero cells have been shown to utilise other receptors [50]. Hence, the replication of EV-A71 might have been hindered due to the cellular receptor restrictions on the viral spread, hence accounting for the lower replication efficiency in the Vero cells. The recent elucidation of the structure of SCARB2 through cryo-electron microscopy revealed that the main sites of contact with the virion of EV-A71 were in the GH loop of VP1 and EF loop of VP2 [51]. This suggested that spontaneous mutations in the EV-A71/SP isolated from RD cells had to adapt to different cell receptors on the Vero cells and this might have been responsible for the difference observed in the growth kinetics and viral binding assays conducted. However, to strengthen this hypothesis, further sequencing data of the EV-A71 plaque variants isolated in Vero cells would be required in future studies. Alternative receptor requirements of the Vero cells might have also been responsible for the discrepancy reported between the rates of replication (10^9^ viral copy number) and infectivity (10^6^ PFU/mL), indicating that there might be a higher ratio of viruses undergoing replication but they were unable to attach to effective receptors in Vero cells, hence leading to a reduction of infectious viral particles.

The initial observation of different plaque forming abilities as reflected by different rates of replication and the rates of infectivity of EV-A71/BP and EV-A71/SP variants indicated that there were significant differences between them. Thus, in order to further verify the veracity of these observations, three additional EV-A71/BP and EV-A71/SP variants were isolated from RD cells, tested in vitro and their full genomes were sequenced. It is believed that the number of isolates in the present study were sufficient to draw conclusions pertaining to the in vitro virulence and amino acid differences observed in the two plaque variants. The presence of novel mutations in the VP1 which were unique and consistent in the EV-A71/BP and EV-A71/SP variants were observed.

Previous studies by Wang et al. (2004) showed that passages of an EV-A71 4643 strain multiple times in mice produced a mouse adapted infectious clone MP4. This strain MP4 exhibited a larger plaque size that grew quickly and was more cytotoxic in vitro than strain 4643 [52]. However, Huang et al. (2012) demonstrated that the introduction of a single mutation VP1-145E of glutamine to glutamic acid or VP2-149M of lysine to methionine in the 4643 strain could lead to the production of enlarged plaques similar to those observed on the MP4 in RD cells. In contrast, the 4643 strain bearing the VP1-283S led to the production of small plaques which suggested that VP1-145E and VP2-149M encouraged the spread of the MP4 virus strain. The simultaneous introduction of both mutations (VP1-145E and VP2-149M) was reported to increase the growth kinetics and spread of the two plaque variants in RD cells. Moreover, the combination of two mutations was also observed to cooperatively increase the viral infectivity which led to cytotoxicity in vitro and lethality in mice in vivo. This was speculated to be due to a rise in viral binding ability and accumulation of EV-A71 RNA in infected Neuro-2a cells incurred by a synergistic effect of both mutations. In our study, the EV-A71/BP and EV-A71/SP carried different mutations in their respective genomes and thus would need reverse genetics studies to investigate the impact of each of these mutations on the plaque morphology, as well as the effects of these combined mutations on virulence of the EV-A71 plaque variants [53].

Other studies focusing on poliovirus revealed some striking similarities to EV-A71. For instance, eight mutations identified in the poliovirus conferring resistance to neutralization were located in the VP1 region. It was suggested that these regions constituted a major antigenic site involving neutralization [54]. Hence, the presence of mutations in the VP1 of the poliovirus and the EV-A71/BP and EV-A71/SP plaque variants indicated that they could impact the antigenicity of the EV-A71 in the same way as the poliovirus. This deduction follows the rationale that EV-A71 has a genome structure much like poliovirus. However, further in vitro and in vivo studies would be required to confirm this claim. Two different Mahoney type I polioviruses were also observed to carry mutations in their VP1 (VP1-101 and VP1-102) and they had an impact on the late stage virus entry into the cell and the release of viral RNA from the capsid. Moreover, both mutant poliovirus strains formed small plaques in HeLa and CV1 cells. In fact, the plaque size differences between these two mutant strains and the wild type poliovirus proved useful in demonstrating a primary defect for both mutants in cell entry. Indeed, the first mutation, VP1-102, was suggested to affect RNA encapsidation as well as RNA release while the other mutation, VP1-101, only affected the release of RNA from the viral capsid. Further analysis revealed solid evidence of connections between RNA release in the host cell and RNA encapsidations of the poliovirus. The location of the mutations in the VP1 (VP1-101 and VP1-102) suggested that they played a direct role in the viral RNA release during cell entry and virion morphogenesis [55]. The striking similarities observed in terms of plaque sizes and lower replication rates of RNA between the poliovirus and the EV-A71 suggested that the EV-A71 might be exhibiting similar behavioural patterns as the poliovirus. Indeed, further investigations pertaining to the release of RNA is warranted in the EV-A71 to confirm whether the mutations observed in the EV-A71/BP and EV-A71/SP variants affected the release of the RNA from the capsid and its subsequent encapsidation prior to viral release and transmission.

Homology modelling was important in this study as it showed how each of the mutations could have affected the VP1 viral protein folding and how this might have influenced the interaction with neighbouring amino acid residues. By analyzing the VP1 structure of both the EV-A71/BP and EV-A71/SP variants, it can be inferred that these mutations have the ability to affect the folding capacity of the virus. The analysis of the 3D structure also revealed that the VP1 interaction was not just dependent on the amino acid sequence, but a tertiary structure-based interaction was necessary for proper self-association. The most striking feature reported from the homology modelling data was that all of the mutations present in the EV-A71/BP and the EV-A71/SP variants were located on the capsid surface of the VP1 which was important in virus interactions with the host cells. 

Like other RNA viruses, EV-A71 has been shown to exist as a quasi-species population due to the low fidelity of the RNA-dependent RNA polymerase [23,45]. Thus, the high genetic plasticity allowed EV-A71 to mutate in response to the changes in the environment [56]. In the present study, four mutations were present in the EV-A71/BP variant and three mutations in the EV-A71/SP variant. 

The mutation initially discovered at position 97 in the BC loop was postulated to be a hotspot due to its recurring nature in the EV-A71/BP and EV-A71/SP variants. The BC loop is known to be a dominant immunogenic region (residue 96–102) for enteroviruses such as poliovirus, EV-A71 and coxsackie viruses [57,58]. Besides its immunogenic role, the BC loop region of VP1 was also identified as a determinant of host adaptation in the poliovirus [59,60]. As such, a previous in vitro study has successfully demonstrated that substitution at position 97 (97^R^) in the VP1 conferred a significant advantage to EV-A71 to grow in neural-derived cells independent of the virus lineage [61].

In addition, N104S observed in the EV-A71/BP variant is also of particular interest due to its location near the 5-fold axis on the surface of the capsid. Lyu et al. (2015) reported that amino residues of the EV-A71 genotype C4 (AH08/06; GenBank accession no. HQ611148) with residues at position 95 to 105 were located on the surface of the capsid protein near the 5-fold axis [62]. The “canyon” is a depression that encircled the 5-fold axes on the surface of EV-A71 that harboured the receptor binding site [63]. This is of relative importance as the virus binding to cell receptors occurred in the canyon-like depression surrounding the 5-fold axis area [64]. Subsequently, this allowed conformational changes in the virus, leading to externalization of the VP1 N-terminus and VP4 which allowed the extrusion of the viral genome in the cytoplasm [65]. Thus, the N104S might exert an impact on the EV-A71/BP variant binding to the host cells due to its proximity to the canyon.

Nishimura et al. (2013) previously presented evidence that the virus-receptor interaction was dependent on amino acid residues VP1-145 and VP1-244. Binding of the EV-A71 to the cell receptor, PSGL-1, was dependent on VP1-145 which was found in the DE loop of the VP1 and it functioned by influencing the orientation of the lysine, Lys^244^_,_ which was located within the HI loop on the viral surface. It was proposed that VP1-145 mediated virus tropism by altering the availability of the positively charged lysine side chain to bind to the negatively charged sulphated N-terminus of PSGL-1, thereby affecting the binding capacity of EV-A71 [66].

Furthermore, the amino acid residue at position 237 was observed to interact with a neighbouring glycine residue at position 105, Gly^105^. Substitution of Asn^237^ with Thr^237^ caused the Gly^105^ residue to be oriented in such a way that hydrogen bonding became possible. Interestingly, Gly^105^ is one of the residues that made up the hydrophobic β-barrel core of the VP1. The pocket factor binding site is located inside the β-barrel core region of the VP1 that holds the fatty acid molecule and sits below the canyon harbouring the receptor binding site [67,68]. Hence, it is possible that the hydrogen bonding with Gly^105^ altered the hydrophobic state of the β-barrel core and ultimately affected the viral binding capability. Indeed, analysis of the 3D structure of the EV-A71 revealed that the core structure of the β-barrel core was significantly smaller in the EV-A71/SP variant than the EV-A71/WT.

The mutation exhibiting the most significant change in the EV-A71/BP variant was N282D whereby the substitution resulted in the presence of a negatively charged aspartic acid at position 282 (Asp^282^) of the C-terminus of the VP1 of EV-A71/BP variant. Meanwhile, the EV-A71/SP variant was also observed to display the substitution Ala^292^ at the C-terminus of the VP1. Indeed, several other viruses have been reported to accumulate mutations on the surface of major surface proteins which resulted in antigenic drift. The sequential antigenic drift of influenza virus via the evolution of the hemagglutinin (HA) protein is a well-illustrated example of antigenic drift [69]. While enteroviruses such as poliovirus (PV) and EV-A71 do not often display antigenic drift in order to escape immune mechanisms, they do exhibit evolving antigenic properties [70]. For instance, the oral polio vaccine (OPV) strain has been shown to mutate and gave rise to pathogenic vaccine-derived poliovirus (VDPV) strains which acquired amino acid substitutions as an evolutionary response to immunological pressure [71]. It is likely that mutations such as N282D might exhibit altered antigenic properties due to its exposed location on the VP1 protein. As antigenic changes in EV-A71 are believed to occur very often in naturally occurring RNA viruses, these mutations highlighted the importance of possible alterations in antigenic sites which are responsible for viral antigenicity. The mutations that could lead to possible altered antigenicity would require further investigations.

The extent of the changes in the secondary structure were also observed to affect the GH loop of the EV-A71 VP1 structure in both the EV-A71/BP and EV-A71/SP variants. Studies have reported that the GH loop is typically a small α-helix [72]. However, a loss of the α-helix was observed as it was altered to a 3_10_-helix structure in the EV-A71/BP variant and a coiled structure in the EV-A71/SP variant. This loss of α-helix is of particular importance as any changes in the VP1 that led to alterations of the GH loop might have an impact on the viral binding ability of the virus as well as affecting its viral uncoating process. The VP1 is the main constituent of the canyon and hydrophobic pocket. Variations in the amino acids of the VP1 especially on the inner surface of the canyon would have a strong impact on the binding of the virus to the receptor and thus influence the viral load and infectivity in the host [73]. More specifically, the role of the GH loop in the VP1 was identified as an adaptor-sensor for cellular receptor attachment which preceded uncoating mechanisms [68].

Lyu et al. (2015) reported that the mutations in the VP1 of the EV-A71 genotype C4 led to changes in the GH loop from 3_10_-helix conformation to a loop structure from amino acid residues 218–221. In other capsid proteins such as VP0, amino acid residues 218–221 also changed from helical structure to a loop conformation following conformational changes within the protein structure [74]. These findings are very similar to what was observed in the VP1 of the EV-A71/BP variant of this study. Hence, it is not uncommon for mutations in the VP1 to impact the conformation of the VP1 protein and in particular, the GH loop. It is interesting to note that the Pro^213^ mutation led to the alteration of the GH loop of CV-A10 as it formed a loop structure rather than the typical α-helix conformation observed in enteroviruses such as EV-A71 and CV-A16 [72]. In turn, this led to the shaping of a different canyon environment in the CV-A10 virus. In the current investigation, similar findings were also observed in the EV-A71/SP variant which formed a coiled GH loop rather than displaying an α-helix. It would be interesting to further explore whether the coiled GH loop structure of the EV-A71/SP variant led to the altered canyon environment as observed by Chen et al. (2019).

On the other hand, there is a possibility that the changes in the secondary structure of the VP1 by the hydrogen bonding interactions due to the mutations might have affected the viral uncoating of the EV-A71 after viral binding and prior to RNA replication. However, the extent to which viral uncoating was hindered as a result of the mutations still remained to be assessed. This claim is in agreement with a previous study which pointed out that mutations affected the capacity of the viral genome to be released after a series of conformational re-arrangements. Indeed, the release of the RNA genome by poliovirus was preceded by the externalization of the VP1 N-terminus and the VP4 which formed a channel to enable the safe transfer of the viral genome [65,75,76]. Lyu et al. (2014) also suggested that the BC, EF and HI loops played an important role in the externalization of the VP1 protein for viral uncoating [62]. Therefore, it could be suggested that alterations in the VP1 structure of the EV-A71/BP and EV-A71/SP variants might have affected the viral uncoating process.

Other studies have also reported that modifications in the VP1 through the replacement of amino acids within the GH loop might have influenced how other capsid proteins such as VP2, VP3 and VP4 might associate to form the T = 3 arrangement. For instance, Lyu et al. (2015) reported that the replacement of residues at VP1 GH loop was able to cause the re-arrangement of local interactions which was propagated to other capsid regions and led to conformational changes locally within the VP1 and remotely in other capsid proteins such as VP0 and VP3 [74].

By analysis of the VP1 structure of both the EV-A71/BP and EV-A71/SP variants and the impact of spontaneous mutations on viral folding, the data suggested that these mutations had the ability to affect the binding capacity of the virus to the RD and Vero cell receptors due to an altered GH loop or smaller hydrophobic pocket. The EV-A71/SP variant exhibited the least binding capability (reduction of 17%) due to its smaller hydrophobic pocket and the altered GH loop. 

Huang et al. (2015) identified substitutions of amino acids at VP1-98K, VP1-145Q and VP1-164E which influenced the antigenic properties of EV-A71 synergistically [70]. Similar trends were also observed in different picornaviruses whereby target mutations introduced in the VP1 led to defective viral binding with susceptible cells [77,78]. There is a possibility that the mutations identified in the EV-A71/SP variant could be adopting similar behaviour to affect the viral binding ability of the virus.

However, there were lesser binding of the EV-A71/BP and EV-A71/SP variants in Vero when compared to RD cells. This would imply a lower frequency of viral entry of the EV-A71/WT, EV-A71/BP and EV-A71/SP variants into Vero cells as well as reduced infectivity in contrast to the RD cells. Cordey et al. (2012) suggested that such an observation implied that compensatory events might have occurred in subsequent stages of the virus life cycle such as RNA replication or virion assembly [61]. It appears that the mutations located in the VP1 of the EV-A71/SP variant affected its binding capability. However, it cannot be ruled out that these substitutions might also affect other stages prior to RNA replication such as viral uncoating or later stages such as virion assembly [61]. 

### Future Directions

To further complement this study, it would be interesting to observe the plaque morphology of the EV-A71/WT and the plaque variants in additional cell lines such as human neuroblastoma cells, SK-N-SH and SH-SY5Y, in which EV-A71 was shown to aggressively replicate and display efficient production of viruses [38,79]. The exceedingly large population size of EV-A71 (10^8^ to 10^9^) in RD cells would affect its evolutionary outcome [80]. Therefore, to fully understand and explain the behaviour and growth properties of the EV-A71 virus in a population, knowledge of a larger amount of genetic information is required [81]. Hence, there is a need to further strengthen the observation of the role of these consistent mutations on the in vitro behaviour of the EV-A71/BP and EV-A71/SP variants. The course of action would be to sequence more EV-A71/BP and EV-A71/SP variants that were passaged at length in both RD and Vero cells and sequence them so as to assess whether there are any more mutations that might have arisen as a result of selective environmental pressure or cell-specific mutations in the plaque variants. This would effectively narrow the search to find specific mutation(s) being the molecular determinant of plaque size or virulence of the small plaque variant. Another approach to explore would be through next generation sequencing (NGS) which provides an in-depth analysis of the frequency of occurrence and the nucleotide sequence similarity of the variants within a population. An NGS approach would give an idea of the overall genetic coverage of the mutations by analysing the entire population of the EV-A71/WT sub-genotype B4 strain 41. Since the presence of two quasi-species in the WT population (EV-A71/BP and EV-A71/SP) were displaying different mutations and the genotypes were stable upon passages, identifying the frequency of occurrence of additional variants and identifying the amino acid substitutions in the other variants present in the population would help steer the study in the right direction.

## 5. Conclusions

In conclusion, this study shows the successful isolation of at least two EV-A71 plaque variants of different sizes from the EV-A71/WT population of sub-genotype B4 strain 41. Successful characterization of the plaque variants in two cell lines showed that the EV-A71/BP and EV-A71/SP variants have different levels of virulence in RD and Vero cells. Furthermore, the results supported the hypothesis that there were novel spontaneous mutations in the VP1 which were unique and consistent in the EV-A71/BP and EV-A71/SP variants. These mutations were responsible for differences in the levels of in vitro virulence observed in this study. Homology modelling analysis indicated that the spontaneous mutations observed in the EV-A71 plaque variants affected the folding capacity of the VP1 capsid protein. The mutations present in the EV-A71/SP variant have led to the alteration of its GH loop and gave rise to a smaller hydrophobic pocket which could potentially affect the viral binding ability to host cell receptors. Indeed, the viral binding assays revealed that the EV-A71/SP variant showed the lowest binding capability in both RD and Vero cells when compared to the EV-A71/BP variant and the EV-A71/WT.

Despite altered structural differences observed in both the EV-A71/BP and EV-A71/SP variants, the EV-A71/BP did not show drastic changes in the in vitro virulence and binding capability as compared to the EV-A71/SP variant. Indeed, this study indicated that the three mutations present in the EV-A71/SP had a more significant impact on the in vitro virulence and binding ability to the cell receptors when compared to both the EV-A71/WT and the EV-A71/BP variant. As such, the mutations of the EV-A71/SP variant should be significant and investigated individually via site-directed mutagenesis (SDM) to further clarify whether they possess significant attenuation of virulence or could alter antigenicity. Further studies should be carried out to quantify the viral attenuation effect of each of the three spontaneous mutations uncovered in this variant via SDM. In view of the rising concerns over EV-A71 infections which had caused fatal large-scale outbreaks in the Asia Pacific region, the results obtained in this current investigation could pave the way towards the rational design of a genetically stable LAV vaccine against EV-A71 infections.

## Figures and Tables

**Table 1:** Plaque forming ability of EV-A71 big plaque variants and small plaque variants in RD and Vero cells.

Plaque Variants of EV-A71 *	Viral Titre in RD Cells (PFU/mL)	Viral Titre in Vero Cells (PFU/mL)
SP1	3.7 × 10^8^	6.40 × 10^3^
SP2	8.7 × 10^8^	6.70 × 10^3^
SP3	9.9 × 10^8^	6.40 × 10^3^
SP4	7.0 × 10^8^	4.70 × 10^3^
BP1	5.4 × 10^9^	1.39 × 10^4^
BP2	5.5 × 10^9^	1.18 × 10^4^
BP3	6.4 × 10^9^	1.44 × 10^4^
BP4	8.1 × 10^9^	1.25 × 10^4^

* SP1–SP4 denotes all small plaque variants and BP1–BP4 denotes all big plaque variants used in this study. Viral titres were quantified in Rhabdomyosarcoma (RD) and Vero cells, respectively.

**Table 2:** Comparison of structural analysis of the EV-A71 plaque variants compared to the wild-type.

EV-A71 Variants	RMSD/Å	Hydrogen Bonds Interactions	Secondary Structure Content
EV-A71/WT	0.00	108	9.4% helix, 23.9% sheet, 17.5% turn, 47.8% coil, 1.3% 3_10_ helix.
EV-A71/BP	1.76	104	6.7% helix, 23.2% sheet, 20.9% turn, 47.5% coil, 1.7% 3_10_ helix.
EV-A71/SP	1.35	107	7.4% helix, 23.2% sheet, 19.9% turn, 48.1% coil, 1.3% 3_10_ helix.

## Supplementary Materials

The following are available online at https://www.mdpi.com/1999-4915/12/6/651/s1, Table S1: Primers used for the whole genome sequencing of the EV-A71 genome., Table S2: PSI-BLAST of the VP1 protein sequence of the EV-A71 parental strain., Table S3: Unpaired t-test analysis of the EV-A71/BP and the EV-A71/SP plaque variant using GraphPad Prism., Figure S1: Agarose gel electrophoresis of the full-length genomic cDNA of EV-A71/WT., Table S4: Amino acid changes observed on the genomes of EV-A71/BP and EV-A71/SP variants., Figure S2: Raw genome sequencing data of EV-A71/BP and EV-A71/SP., Figure S3: The 3D structure of the VP1 protein of the EV-A71/WT superposed against the EV-A71/BP and EV-A71/SP variants.

## References

[1] Schmidt N.J., Lennette E.H., Ho H.H. (1974). An apparently new enterovirus isolated from patients with disease of the central nervous system. J. Infect. Dis..

[2] Brown B.A., Oberste M.S., Alexander J.P., Kennett M.L., Pallansch M.A. (1999). Molecular epidemiology and evolution of enterovirus 71 strains isolated from 1970 to 1998. J. Virol..

[3] Chumakov M., Voroshilova M., Shindarov L., Lavrova I., Gracheva L., Koroleva G., Vasilenko S., Brodvarova I., Nikolova M., Gyurova S. (1979). Enterovirus 71 isolated from cases of epidemic poliomyelitis-like disease in Bulgaria. Arch. Virol..

[4] Nagy G., Takatsy S., Kukan E., Mihaly I., Domok I. (1982). Virological diagnosis of enterovirus type 71 infections: Experiences gained during an epidemic of acute CNS diseases in Hungary in 1978. Arch. Virol..

[5] Van der Sanden S.M.G., Koopmans M.P.G., Verduyn-Lunel F., van der Avoort H., Galama J.M.D. (2009). Epidemiology of enterovirus 71 in the Netherlands, 1963–2007: Change of dominant subgenogroup in time. J. Clin. Virol..

[6] Xing W., Liao Q., Viboud C., Zhang J., Sun J., Wu J.T., Chang Z., Liu F., Fang V.J., Zheng Y. (2014). Hand, foot, and mouth disease in China, 2008–2012: An epidemiological study. Lancet Infect. Dis.

[7] Chan L.G., Parashar U.D., Lye M.S., Ong F.G.L., Zaki S.R., Alexander J.P., Ho K.K., Han L.L., Pallansch M.A., Abu Bakar S. (2000). Deaths of children during an outbreak of hand, foot, and mouth disease in Sarawak, Malaysia: Clinical and pathological characteristics of the disease. Clin. Infect. Dis..

[8] Wang J.R., Tuan Y.C., Tsai H.P., Yan J.J., Liu C.C., Su I.J. (2002). Change of major genotype of enterovirus 71 in outbreaks of hand-foot-and-mouth disease in Taiwan between 1998 and 2000. J. Clin. Microbiol..

[9] Cardosa M.J., Krishnan S., Tio P.H., Perera D., Wong S.C. (1999). Isolation of subgenus B adenovirus during a fatal outbreak of enterovirus 71-associated hand, foot, and mouth disease in Sibu, Sarawak. Lancet.

[10] Brown B.A., Pallansch M.A. (1995). Complete nucleotide sequence of Enterovirus 71 is distinct from poliovirus. Virus Res..

[11] Bessaud M., Razafindratsimandresy R., Nougairede A., Joffret M.L., Deshpande J.M., Dubot-Peres A., Heraud J.M., de Lamballerie X., Delpeyroux F., Bailly J.L. (2014). Molecular comparison and evolutionary analyses of VP1 nucleotide sequences of new African human enterovirus 71 isolates reveal a wide genetic diversity. PLoS ONE.

[12] Solomon T., Lewthwaite P., Perera D., Cardosa M.J., McMinn P., Ooi M.H. (2010). Virology, epidemiology, pathogenesis, and control of Enterovirus 71. Lancet Infect. Dis..

[13] Volle R., Razafindratsimandresy R., Joffret M.L., Bessaud M., Rabemanantsoa S., Andriamamonjy S., Raharinantoanina J., Blondel B., Heraud J.M., Bailly J.L. (2019). High permissiveness for genetic exchanges between enteroviruses of species A, including enterovirus 71, favors evolution through intertypic recombination in Madagascar. J. Virol..

[14] Mandary M.B., Poh C.L. (2018). Changes in the EV-A71 genome through recombination and spontaneous mutations: Impact on virulence. Viruses.

[15] Huang S.W., Kiang D., Smith D.J., Wang J.R. (2011). Evolution of re-emergent virus and its impact on enterovirus 71 epidemics. Exp. Biol. Med..

[16] AbuBakar S., Chee H.Y., Al-Kobaisi M.F., Xiaoshan J., Chua K.B., Lam S.K. (1999). Identification of enterovirus 71 isolates from an outbreak of hand, foot and mouth disease (HFMD) with fatal cases of encephalomyelitis in Malaysia. Virus Res..

[17] Podin Y., Gias E.L.M., Ong F., Leong Y.W., Yee S.F., Yusof M.A., Perera D., Teo B., Wee T.Y., Yao S.C. (2006). Sentinel surveillance for human enterovirus 71 in Sarawak, Malaysia: Lessons from the first 7 years. BMC Public Health.

[18] Caine E.A., Moncla L.H., Ronderos M.D., Friedrich T.C., Osorio J.E. (2016). A single mutation in the VP1 of enterovirus 71 is responsible for increased virulence and neurotropism in adult interferon-deficient mice. J. Virol..

[19] Li P., Yue Y., Song N., Li B., Meng H., Yang G., Li Z., An L., Qin L. (2016). Genome analysis of enterovirus 71 strains differing in mouse pathogenicity. Virus Genes.

[20] Singh S., Poh C.L., Chow V.T. (2002). Complete sequence analyses of enterovirus 71 strains from fatal and non-fatal cases of the hand, foot and mouth disease outbreak in Singapore. Microbiol. Immunol..

[21] Yeh M.T., Wang S.W., Yu C.K., Lin K.H., Lei H.Y., Su I.J., Wang J.R. (2011). A single nucleotide in stem loop II of 5′-untranslated region contributes to virulence of enterovirus 71 in mice. PLoS ONE.

[22] Yuan S., Li G., Wang Y., Gao Q., Wang Y., Cui R., Altmeyer R., Zou G. (2015). Identification of positively charged residues in enterovirus 71 capsid protein VP1 essential for production of infectious particles. J. Virol..

[23] Domingo E., Holland J.J. (1997). RNA virus mutations and fitness for survival. Annu. Rev. Microbiol..

[24] Eckels K.H., Brandt W.E., Harrison V.R., McCown J.M., Russell P.K. (1976). Isolation of a temperature-sensitive dengue-2 virus under conditions suitable for vaccine development. Infect. Immun..

[25] Goh K.C.M., Tang C.K., Norton D.C., Gan E.S., Tan H.C., Sun B., Syenina A., Yousuf A., Ong X.M., Kamaraj U.S. (2016). Molecular determinants of plaque size as an indicator of dengue virus attenuation. Sci. Rep..

[26] Wu S.-C., Lian W.-C., Hsu L.-C., Liau M.-Y. (1997). Japanese encephalitis virus antigenic variants with characteristic differences in neutralization resistance and mouse virulence. Virus Res..

[27] Blaney J.E., Johnson D.H., Firestone C.-Y., Hanson C.T., Murphy B.R., Whitehead S.S. (2001). Chemical mutagenesis of dengue virus type 4 yields mutant viruses which are temperature sensitive in Vero cells or human liver cells and attenuated in mice. J. Virol..

[28] Blaney J.E., Manipon G.G., Murphy B.R., Whitehead S.S. (2003). Temperature sensitive mutations in the genes encoding the NS1, NS2A, NS3, and NS5 nonstructural proteins of dengue virus type 4 restrict replication in the brains of mice. Arch. Virol..

[29] Blaney J.E., Johnson D.H., Manipon G.G., Firestone C.-Y., Hanson C.T., Murphy B.R., Whitehead S.S. (2002). Genetic basis of attenuation of dengue virus type 4 small plaque mutants with restricted replication in suckling mice and in SCID mice transplanted with human liver cells. Virology.

[30] Chambers T.J., Nickells M. (2001). Neuroadapted yellow fever virus 17D: Genetic and biological characterization of a highly mouse-neurovirulent virus and its infectious molecular clone. J. Virol..

[31] Nickells M., Chambers T.J. (2003). Neuroadapted yellow fever virus 17D: Determinants in the envelope protein govern neuroinvasiveness for SCID mice. J. Virol..

[32] Burgon T.B., Jenkins J.A., Deitz S.B., Spagnolo J.F., Kirkegaard K. (2009). Bypass suppression of small-plaque phenotypes by a mutation in poliovirus 2A that enhances apoptosis. J. Virol..

[33] Jia Y., Moudy R.M., Dupuis A.P., Ngo K.A., Maffei J.G., Jerzak G.V., Franke M.A., Kauffman E.B., Kramer L.D. (2007). Characterization of a small plaque variant of West Nile virus isolated in New York in 2000. Virology.

[34] Kato F., Tajima S., Nakayama E., Kawai Y., Taniguchi S., Shibasaki K., Taira M., Maeki T., Lim C.K., Takasaki T. (2017). Characterization of large and small-plaque variants in the Zika virus clinical isolate ZIKV/Hu/S36/Chiba/2016. Sci. Rep..

[35] Jaimipak T., Yoksan S., Ubol S., Pulmanausahakul R. (2018). Small plaque size variant of Chikungunya primary isolate showed reduced virulence in mice. Asian Pac. J. Allergy Immunol..

[36] Tan C.W., Chan Y.F., Sim K.M., Tan E.L., Poh C.L. (2012). Inhibition of Enterovirus 71 (EV-71) infections by a novel antiviral peptide derived from EV-71 capsid protein VP1. PLoS ONE.

[37] Alexander J., James P., Baden L., Pallansch M.A., Anderson L.J. (1994). Enterovirus 71 infections and neurologic disease—United States, 1977–1991. J. Infect. Dis..

[38] Wen Y.Y., Chang T.Y., Chen S.T., Li C., Liu H.S. (2003). Comparative study of enterovirus 71 infection of human cell lines. J. Med. Virol..

[39] Moser L.A., Boylan B.T., Moreira F.R., Myers L.J., Svenson E.L., Fedorova N.B., Pickett B.E., Bernard K.A. (2018). Growth and adaptation of Zika virus in mammalian and mosquito cells. PLoS Negl. Trop. Dis..

[40] Schade-Weskott M.L., van Schalkwyk A., Koekemoer J.J.O. (2018). A correlation between capsid protein VP2 and the plaque morphology of African horse sickness virus in cell culture. Virus Genes.

[41] Anderson B.D., Barr K.L., Heil G.L., Friary J.A., Gray G.C. (2012). A comparison of viral fitness and virulence between emergent adenovirus 14p1 and prototype adenovirus 14p strains. J. Clin. Virol..

[42] Kanno T., Mackay D., Inoue T., Wilsden G., Yamakawa M., Yamazoe R., Yamaguchi S., Shirai J., Kitching P., Murakami Y. (1999). Mapping the genetic determinants of pathogenicity and plaque phenotype in swine vesicular disease virus. J. Virol..

[43] Huang S.W., Huang Y.H., Tsai H.P., Kuo P.H., Wang S.M., Liu C.C., Wang J.R. (2017). A selective bottleneck shapes the evolutionary mutant spectra of enterovirus A71 during viral dissemination in humans. J. Virol..

[44] Eigen M., Schuster P. (1978). The hypercycle. Naturwissenschaften.

[45] Vignuzzi M., Stone J.K., Arnold J.J., Cameron C.E., Andino R. (2006). Quasispecies diversity determines pathogenesis through cooperative interactions in a viral population. Nature.

[46] Holland J., Spindler K., Horodyski F., Grabau E., Nichol S., VandePol S. (1982). Rapid evolution of RNA genomes. Science.

[47] Li X., Fan P., Jin J., Su W., An D., Xu L., Sun S., Zhang Y., Meng X., Gao F. (2013). Establishment of cell lines with increased susceptibility to EV71/CA16 by stable overexpression of SCARB2. Virol. J..

[48] Li Z.H., Yue Y.Y., Li P., Song N.N., Li B., Zhang Y., Meng H., Jiang G.S., Qin L. (2015). MA104 Cell line presents characteristics suitable for enterovirus A71 isolation and proliferation. Microbiol. Immunol..

[49] Yamayoshi S., Yamashita Y., Li J., Hanagata N., Minowa T., Takemura T., Koike S. (2009). Scavenger receptor B2 is a cellular receptor for enterovirus 71. Nat. Med..

[50] Lin Y.W., Lin H.Y., Tsou Y.L., Chitra E., Hsiao K.N., Shao H.Y., Liu C.C., Sia C., Chong P., Chow Y.H. (2012). Human SCARB2-mediated entry and endocytosis of EV71. PLoS ONE.

[51] Zhou D., Zhao Y., Kotecha A., Fry E.E., Kelly J.T., Wang X., Rao Z., Rowlands D.J., Ren J., Stuart D.I. (2019). Unexpected mode of engagement between enterovirus 71 and its receptor SCARB2. Nat. Microb..

[52] Wang Y.F., Chou C.T., Lei H.Y., Liu C.C., Wang S.M., Yan J.J., Su I.J., Wang J.R., Yeh T.M., Chen S.H. (2004). A mouse-adapted enterovirus 71 strain causes neurological disease in mice after oral infection. J. Virol..

[53] Huang S.W., Wang Y.F., Yu C.K., Su I.J., Wang J.R. (2012). Mutations in VP2 and VP1 capsid proteins increase infectivity and mouse lethality of enterovirus 71 by virus binding and RNA accumulation enhancement. Virology.

[54] Evans D.M., Minor P.D., Schild G.S., Almond J.W. (1983). Critical role of an eight-amino acid sequence of VP1 in neutralization of poliovirus type 3. Nature.

[55] Kirkegaard K. (1990). Mutations in VP1 of poliovirus specifically affect both encapsidation and release of viral RNA. J. Virol..

[56] Tan C.W., Sam I.C., Lee V.S., Wong H.V., Chan Y.F. (2017). VP1 residues around the five-fold axis of enterovirus A71 mediate heparan sulfate interaction. Virology.

[57] Graham S., Wang E.C., Jenkins O., Borysiewicz L.K. (1993). Analysis of the human T-cell response to picornaviruses: Identification of T-cell epitopes close to B-cell epitopes in poliovirus. J. Virol..

[58] Reimann B.Y., Zell R., Kandolf R. (1991). Mapping of a neutralizing antigenic site of coxsackievirus B4 by construction of an antigen chimera. J. Virol..

[59] La Monica N., Kupsky W.J., Racaniello V.R. (1987). Reduced mouse neurovirulence of poliovirus type 2 Lansing antigenic variants selected with monoclonal antibodies. Virology.

[60] Martin A., Wychowski C., Couderc T., Crainic R., Hogle J., Girard M. (1988). Engineering a poliovirus type 2 antigenic site on a type 1 capsid results in a chimaeric virus which is neurovirulent for mice. EMBO J..

[61] Cordey S., Petty T.J., Schibler M., Martinez Y., Gerlach D., van Belle S., Turin L., Zdobnov E., Kaiser L., Tapparel C. (2012). Identification of site-specific adaptations conferring increased neural cell tropism during human enterovirus 71 infection. PLoS Pathog..

[62] Lyu K., Ding J., Han J.F., Zhang Y., Wu X.Y., He Y.L., Qin C.F., Chen R. (2014). Human enterovirus 71 uncoating captured at atomic resolution. J. Virol..

[63] Olson N.H., Kolatkar P.R., Oliveira M.A., Cheng R.H., Greve J.M., McClelland A., Baker T.S., Rossmann M.G. (1993). Structure of a human rhinovirus complexed with its receptor molecule. Proc. Natl. Acad. Sci. USA.

[64] Zhang P., Mueller S., Morais M.C., Bator C.M., Bowman V.D., Hafenstein S., Wimmer E., Rossmann M.G. (2008). Crystal structure of CD155 and electron microscopic studies of its complexes with polioviruses. Proc. Natl. Acad. Sci. USA.

[65] Fricks C.E., Hogle J.M. (1990). Cell-induced conformational change in poliovirus: Externalization of the amino terminus of VP1 is responsible for liposome binding. J. Virol..

[66] Nishimura Y., Lee H., Hafenstein S., Kataoka C., Wakita T., Bergelson J.M., Shimizu H. (2013). Enterovirus 71 binding to PSGL-1 on leukocytes: VP1-145 acts as a molecular switch to control receptor interaction. PLoS Pathog..

[67] Ren J., Wang X., Zhu L., Hu Z., Gao Q., Yang P., Li X., Wang J., Shen X., Fry E.E.R. (2015). Structures of coxsackievirus A16 capsids with native antigenicity: Implications for particle expansion, receptor binding, and immunogenicity. J. Virol..

[68] Wang X., Zhu C., Bao W., Zhao K., Niu J., Yu X.F., Zhang W. (2012). Characterization of full-length enterovirus 71 strains from severe and mild disease patients in northeastern China. PLoS ONE.

[69] Barr I.G., McCauley J., Cox N., Daniels R., Engelhardt O.G., Fukuda K., Grohmann G., Hay A., Kelso A., Klimov A. (2010). Epidemiological, antigenic and genetic characteristics of seasonal influenza A(H1N1), A(H3N2) and B influenza viruses: Basis for the WHO recommendation on the composition of influenza vaccines for use in the 2009-2010 northern hemisphere season. Vaccine.

[70] Huang S.W., Tai C.H., Fonville J.M., Lin C.H., Wang S.M., Liu C.C., Su I.J., Smith D.J., Wang J.R. (2015). Mapping enterovirus A71 antigenic determinants from viral evolution. J. Virol..

[71] Chow M., Yabrov R., Bittle J., Hogle J., Baltimore D. (1985). Synthetic peptides from four separate regions of the poliovirus type 1 capsid protein VP1 induce neutralizing antibodies. Proc. Natl. Acad. Sci. USA.

[72] Chen J., Ye X., Zhang X.Y., Zhu Z., Zhang X., Xu Z., Ding Z., Zou G., Liu Q., Kong L. (2019). Coxsackievirus A10 atomic structure facilitating the discovery of a broad-spectrum inhibitor against human enteroviruses. Cell Discov..

[73] Li R., Zou Q., Chen L., Zhang H., Wang Y. (2011). Molecular analysis of virulent determinants of enterovirus 71. PLoS ONE.

[74] Lyu K., He Y.L., Li H.Y., Chen R. (2015). Crystal structures of yeast-produced enterovirus 71 and enterovirus 71/coxsackievirus A16 chimeric virus-like particles provide the structural basis for novel vaccine design against hand-foot-and-mouth disease. J. Virol..

[75] Wang X., Peng W., Ren J., Hu Z., Xu J., Lou Z., Li X., Yin W., Shen X., Porta C. (2012). A sensor-adaptor mechanism for enterovirus uncoating from structures of EV71. Nat. Struct. Mol. Biol..

[76] Tuthill T.J., Groppelli E., Hogle J.M., Rowlands D.J. (2010). Picornaviruses. Curr. Top. Microbiol. Immunol..

[77] Colonno R.J., Condra J.H., Mizutani S., Callahan P.L., Davies M.E., Murcko M.A. (1988). Evidence for the direct involvement of the rhinovirus canyon in receptor binding. Proc. Natl. Acad. Sci. USA.

[78] Rossmann M.G., Arnold E., Erickson J.W., Frankenberger E.A., Griffith J.P., Hecht H.J., Johnson J.E., Kamer G., Luo M., Mosser A.G. (1985). Structure of a human common cold virus and functional relationship to other picornaviruses. Nature.

[79] Yan J.J., Wang J.R., Liu C.C., Yang H.B., Su I.J. (2000). An outbreak of enterovirus 71 infection in Taiwan 1998: A comprehensive pathological, virological, and molecular study on a case of fulminant encephalitis. J. Clin. Virol..

[80] Domingo E., Sheldon J., Perales C. (2012). Viral quasispecies evolution. Microbiol. Mol. Biol. Rev. MMBR.

[81] Gong L., Han Y., Chen L., Liu F., Hao P., Sheng J., Li X.H., Yu D.M., Gong Q.M., Tian F. (2013). Comparison of next-generation sequencing and clone-based sequencing in analysis of hepatitis B virus reverse transcriptase quasispecies heterogeneity. J. Clin. Microbiol..

