# Supplementary material for: Characterization of Plaque Variants and the Involvement of Quasi-Species in a Population of EV-A71
Source: Viruses. 2020 Jun 17;12(6):651. doi: 10.3390/v12060651 (PMC7354493; doi:10.3390/v12060651)
Supplement: Supplementary file 1 [file viruses-12-00651-s001.pdf]

# Supplementary Materials

Supplementary Table S1: Primers used for the whole genome sequencing of the EV-A71 genome

| Name of Primer | Sequences (5→ 3) |
|---|---|
| 5'-UTR Forward | TTAAAACAGCTGTGGGTTGTAC |
| 5'-UTR Reverse | GTGGAGCCTTCTGTAGCTGAAT |
| VP4_VP2 Forward | CAATCAAACATGGGCTCACAGG |
| VP4_VP2 Reverse | TGATTCGTTCCTGGTTTTGGCT |
| VP3 Forward | GATTTTGACCAAGGGGCAACTC |
| VP3 Reverse | GCTGGAACTTCACCAGTGTCTA |
| VP1 Forward | GCTTACATAATAGCACTAGCGGC |
| VP1 Reverse | GTAGCGAGGTGACGATTAACCA |
| 2A_2B Forward | ACTGTGGGTTCATCATCAAAGTCCA |
| 2A_2B Reverse | CTGCTGGAACAATCTTCTCCCT |
| 2C Forward | AACCTGATTTCAGCGCTAGTGA |
| 2C Reverse | AATCCAGCCTTGTTCCCTACAG |
| 3A_3B_3C Forward | GTCGCAGGTTCTACATGGATTG |
| 3A_3B_3C Reverse | AAACAGGGCTTGTTCAAAGTCG |
| 3D_3'-UTR Forward | AAGAGGAGCTACTTTGCGAG |
| 3D_3'-UTR Reverse | GCTATTCCGGTTATAACAAATTTAACC |

Supplementary Table S2: PSI-BLAST of the VP1 protein sequence of the EV-A71 parental strain.

[1] The templates were chosen by protein blast of the VP1 protein sequence of the EV-A71 parental

| No. | PDB ID | Percentage Identity (%) | Query Cover (%) | Resolution (Å) |
|---|---|---|---|---|
| 1 | 4AED_A | 99.33 | 100 | 3.80 |
| 2 | 612K_A | 97.64 | 100 | 3.40 |
| 3 | 4CDQ-_A | 95.96 | 100 | 2.65 |
| 4 | 3VBF_A | 95.62 | 100 | 2.60 |

strain in NCBI using PDB database and PSI-BLAST (Position-Specific Iterated BLAST) algorithm.

Supplementary Table S3: Unpaired t-test analysis of the EV-A71/BP and the EV-A71/SP plaque variant using GraphPad Prism.

| Unpaired t-test analysis: | MEAN PLAQUE SIZES RD | MEAN PLAQUE SIZES VERO |
|---|---|---|
| | | |
| Column B | BP | BP |
| vs. | vs. | vs. |
| Column A | SP | SP |
| Unpaired t test | | |
| P value | <0.0001 | <0.0001 |
| P value summary | **** | **** |
| Significantly different (P < 0.05)? | Yes | Yes |
| One- or two-tailed P value? | Two-tailed | Two-tailed |
| t, df | t=48.1 df=794 | t=62.85 df=780 |
| How big is the difference? | | |
| Mean ± SEM of column A | 293 ± 2.021, n=399 | 176.7 ± 1.68, n=408 |
| Mean ± SEM of column B | 518.6 ± 4.239, n=397 | 380.6 ± 2.851, n=394 |
| Difference between means | 225.6 ± 4.689 | 203.9 ± 3.245 |
| 95% confidence interval | 216.4 to 234.8 | 197.6 to 210.3 |
| R squared (eta squared) | 0.7445 | 0.8351 |
| | | |
| F test to compare variances | | |
| F, DFn, Dfd | 4.376, 396, 398 | 2.639, 373, 407 |
| P value | <0.0001 | <0.0001 |
| P value summary | **** | **** |
| Significantly different (P < 0.05)? | Yes | Yes |

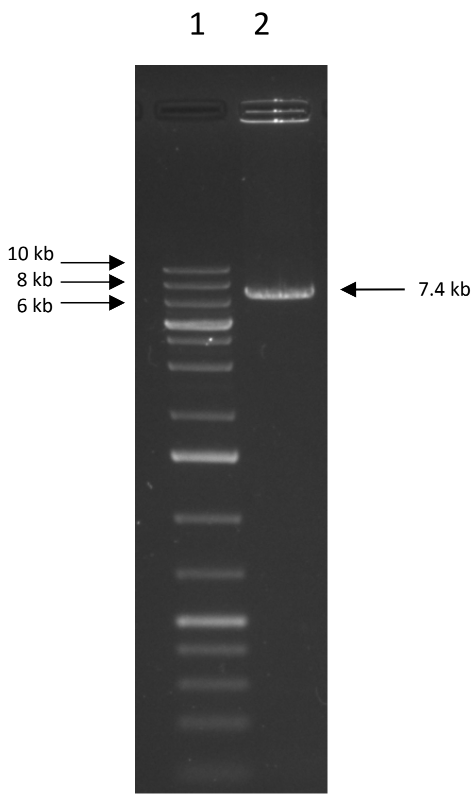


Supplementary Figure S1: Agarose gel electrophoresis of the full-length genomic cDNA of EV-A71/WT. The cDNA was produced with the LongAmp DNA polymerase. (1) Lane 1: DNA ladder (2) Lane 2: cDNA of the full-length EV71 genome, 7400 bp in length.

Supplementary Table S4: Amino acid changes observed on the genomes of EV-A71/BP and EV-A71/SP variants.

| Position of amino acid | EV-A71 Variant | Change in amino acids | Type of mutation |
|---|---|---|---|
| $VP1^{97}$ | EV-A71/BP | Isoleucine (I) → Leucine (L) | Non-synonymous |
| $VP1^{104}$ | EV-A71/BP | Asparagine (N) → Serine (S) | Non-synonymous |
| $VP1^{246}$ | EV-A71/BP | Serine (S) → Proline (P) | Non-synonymous |
| $VP1^{282}$ | EV-A71/BP | Asparagine (N) → Aspartic Acid (D) | Non-synonymous |
| $VP1^{97}$ | EV-A71/SP | Isoleucine (I) → Threonine (T) | Non-synonymous |
| $VP1^{237}$ | EV-A71/SP | Asparagine (N) →Threonine (T) | Non-synonymous |
| $VP1^{292}$ | EV-A71/SP | Threonine (T) → Alanine (A) | Non-synonymous |

*The amino acid sequence of both the EV-A71/BP and EV-A71/SP variants were compared against the genome of the EV-A71/WT.

The amino acid sequence of the EV-A71/WT (parental EV-A71 strain) against EV-A71/BP and EV-A71/SP. Analysis was performed via Geneious Software and Clustal Omega and significant mutations of EV-A71/BP are: I97L, N104S, S246P and N282D. Significant mutations of EV-A71/SP are: I97T, N237T and T292A.

```
BP1     GDRVADVIESSIGDSVSRALTQALPAPTGQNTQVSSHRLDTGEVPALQAAEIGASSNTSD     60
BP2     GDRVADVIESSIGDSVSRALTQALPAPTGQNTQVSSHRLDTGEVPALQAAEIGASSNTSD     60
BP3     GDRVADVIESSIGDSVSRALTQALPAPTGQNTQVSSHRLDTGEVPALQAAEIGASSNTSD     60
BP4     GDRVADVIESSIGDSVSRALTQALPAPTGQNTQVSSHRLDTGEVPALQAAEIGASSNTSD     60
WT      GDRVADVIESSIGDSVSRALTQALPAPTGQNTQVSSHRLDTGEVPALQAAEIGASSNTSD     60
SP1     GDRVADVIESSIGDSVSRALTQALPAPTGQNTQVSSHRLDTGEVPALQAAEIGASSNTSD     60
SP2     GDRVADVIESSIGDSVSRALTQALPAPTGQNTQVSSHRLDTGEVPALQAAEIGASSNTSD     60
SP3     GDRVADVIESSIGDSVSRALTQALPAPTGQNTQVSSHRLDTGEVPALQAAEIGASSNTSD     60
SP4     GDRVADVIESSIGDSVSRALTQALPAPTGQNTQVSSHRLDTGEVPALQAAEIGASSNTSD     60
        ************************************************************

                                           97/97  104
BP1     ESMIETRCVLNSHSTAETTLDSFFSRAGLVGEIDLPLEGTTNPSGYANWDIDITGYAQMR     120
BP2     ESMIETRCVLNSHSTAETTLDSFFSRAGLVGEIDLPLEGTTNPSGYANWDIDITGYAQMR     120
BP3     ESMIETRCVLNSHSTAETTLDSFFSRAGLVGEIDLPLEGTTNPSGYANWDIDITGYAQMR     120
BP4     ESMIETRCVLNSHSTAETTLDSFFSRAGLVGEIDLPLEGTTNPSGYANWDIDITGYAQMR     120
WT      ESMIETRCVLNSHSTAETTLDSFFSRAGLVGEIDLPIEGTTNPNGYANWDIDITGYAQMR     120
SP1     ESMIETRCVLNSHSTAETTLDSFFSRAGLVGEIDLPTEGTTNPNGYANWDIDITGYAQMR     120
SP2     ESMIETRCVLNSHSTAETTLDSFFSRAGLVGEIDLPTEGTTNPNGYANWDIDITGYAQMR     120
SP3     ESMIETRCVLNSHSTAETTLDSFFSRAGLVGEIDLPTEGTTNPNGYANWDIDITGYAQMR     120
SP4     ESMIETRCVLNSHSTAETTLDSFFSRAGLVGEIDLPTEGTTNPNGYANWDIDITGYAQMR     120
        ************************************ ******.****************

BP1     RKVELFTYMRFDAEFTFVACTPTGQVVPQLLQYMFVPPGAPKPESRESLAWQTATNPSVF     180
BP2     RKVELFTYMRFDAEFTFVACTPTGQVVPQLLQYMFVPPGAPKPESRESLAWQTATNPSVF     180
BP3     RKVELFTYMRFDAEFTFVACTPTGQVVPQLLQYMFVPPGAPKPESRESLAWQTATNPSVF     180
BP4     RKVELFTYMRFDAEFTFVACTPTGQVVPQLLQYMFVPPGAPKPESRESLAWQTATNPSVF     180
WT      RKVELFTYMRFDAEFTFVACTPTGQVVPQLLQYMFVPPGAPKPESRESLAWQTATNPSVF     180
SP1     RKVELFTYMRFDAEFTFVACTPTGQVVPQLLQYMFVPPGAPKPESRESLAWQTATNPSVF     180
SP2     RKVELFTYMRFDAEFTFVACTPTGQVVPQLLQYMFVPPGAPKPESRESLAWQTATNPSVF     180
SP3     RKVELFTYMRFDAEFTFVACTPTGQVVPQLLQYMFVPPGAPKPESRESLAWQTATNPSVF     180
SP4     RKVELFTYMRFDAEFTFVACTPTGQVVPQLLQYMFVPPGAPKPESRESLAWQTATNPSVF     180
        ************************************************************

                                                                237
BP1     VKLTDPPAQVSVPFMSPASAYQWFYDGYPTFGEHKQEKDLEYGACPNNMMGTFSVRNVGS     240
BP2     VKLTDPPAQVSVPFMSPASAYQWFYDGYPTFGEHKQEKDLEYGACPNNMMGTFSVRNVGS     240
BP3     VKLTDPPAQVSVPFMSPASAYQWFYDGYPTFGEHKQEKDLEYGACPNNMMGTFSVRNVGS     240
BP4     VKLTDPPAQVSVPFMSPASAYQWFYDGYPTFGEHKQEKDLEYGACPNNMMGTFSVRNVGS     240
WT      VKLTDPPAQVSVPFMSPASAYQWFYDGYPTFGEHKQEKDLEYGACPNNMMGTFSVRNVGS     240
SP1     VKLTDPPAQVSVPFMSPASAYQWFYDGYPTFGEHKQEKDLEYGACPNNMMGTFSVRTVGS     240
SP2     VKLTDPPAQVSVPFMSPASAYQWFYDGYPTFGEHKQEKDLEYGACPNNMMGTFSVRTVGS     240
SP3     VKLTDPPAQVSVPFMSPASAYQWFYDGYPTFGEHKQEKDLEYGACPNNMMGTFSVRTVGS     240
SP4     VKLTDPPAQVSVPFMSPASAYQWFYDGYPTFGEHKQEKDLEYGACPNNMMGTFSVRTVGS     240
        ********************************************************.***

          246                                      282          292
BP1     SKSKYPLVVRIYMRMKHVRAWIPRPMRNQNYLFKANPNYAGDSIKPTGTSRTAITTL   297
BP2     SKSKYPLVVRIYMRMKHVRAWIPRPMRNQNYLFKANPNYAGDSIKPTGTSRTAITTL   297
BP3     SKSKYPLVVRIYMRMKHVRAWIPRPMRNQNYLFKANPNYAGDSIKPTGTSRTAITTL   297
BP4     SKSKYPLVVRIYMRMKHVRAWIPRPMRNQNYLFKANPNYAGDSIKPTGTSRTAITTL   297
WT      SKSKYSLVVRIYMRMKHVRAWIPRPMRNQNYLFKANPNYAGNSIKPTGTSRTAITTL   297
SP1     SKSKYSLVVRIYMRMKHVRAWIPRPMRNQNYLFKANPNYAGNSIKPTGTSRAAITTL   297
SP2     SKSKYSLVVRIYMRMKHVRAWIPRPMRNQNYLFKANPNYAGNSIKPTGTSRAAITTL   297
SP3     SKSKYSLVVRIYMRMKHVRAWIPRPMRNQNYLFKANPNYAGNSIKPTGTSRAAITTL   297
SP4     SKSKYSLVVRIYMRMKHVRAWIPRPMRNQNYLFKANPNYAGNSIKPTGTSRAAITTL   297
        ***** ***********************************:*********:*****
```

SMALL PLAQUE: , BIG PLAQUE: AND WILD TYPE:

Alignment of sequencing results for the EV-A71/BP variant:

**VP4 alignment for big plaque isolates**

```
WT      MGSQVSTQRSGSHENSNSATEGSTINYTTINYYKDSYAATAGKQSLKQDPDKFANPVKDI   60
BP1     MGSQVSTQRSGSHENSNSATEGSTINYTTINYYKDSYAATAGKQSLKQDPDKFANPVKDI   60
BP2     MGSQVSTQRSGSHENSNSATEGSTINYTTINYYKDSYAATAGKQSLKQDPDKFANPVKDI   60
BP3     MGSQVSTQRSGSHENSNSATEGSTINYTTINYYKDSYAATAGKQSLKQDPDKFANPVKDI   60
BP4     MGSQVSTQRSGSHENSNSATEGSTINYTTINYYKDSYAATAGKQSLKQDPDKFANPVKDI   60
        ************************************************************

WT      FTEMAAPLK     69
BP1     FTEMAAPLK     69
BP2     FTEMAAPLK     69
BP3     FTEMAAPLK     69
BP4     FTEMAAPLK     69
        *********
```

**VP2 alignment for the big plaque isolates:**

```
WT      SPSAEACGYSDRVAQLTIGNSTITTQEAANIIVGYGEWPSYCSDDDATAVDKPTRPDVSV      60
BP1     SPSAEACGYSDRVAQLTIGNSTITTQEAANIIVGYGEWPSYCSDDDATAVDKPTRPDVSV      60
BP2     SPSAEACGYSDRVAQLTIGNSTITTQEAANIIVGYGEWPSYCSDDDATAVDKPTRPDVSV      60
BP3     SPSAEACGYSDRVAQLTIGNSTITTQEAANIIVGYGEWPSYCSDDDATAVDKPTRPDVSV      60
BP4     SPSAEACGYSDRVAQLTIGNSTITTQEAANIIVGYGEWPSYCSDDDATAVDKPTRPDVSV      60
        ************************************************************

WT      NRFYTLDTKLWEKSSKGWYWKFPDVLTETGVFGQNAQFHYLYRSGFCIHVQCNASKFHQG      120
BP1     NRFYTLDTKLWEKSSKGWYWKFPDVLTETGVFGQNAQFHYLYRSGFCIHVQCNASKFHQG      120
BP2     NRFYTLDTKLWEKSSKGWYWKFPDVLTETGVFGQNAQFHYLYRSGFCIHVQCNASKFHQG      120
BP3     NRFYTLDTKLWEKSSKGWYWKFPDVLTETGVFGQNAQFHYLYRSGFCIHVQCNASKFHQG      120
BP4     NRFYTLDTKLWEKSSKGWYWKFPDVLTETGVFGQNAQFHYLYRSGFCIHVQCNASKFHQG      120
        ************************************************************

WT      ALLVAILPEYVIGTVAGGTGTEDSHPPYKQTQPGADGFELQHPYVLDAGIPISQLTVCPH      180
BP1     ALLVAILPEYVIGTVAGGTGTEDSHPPYKQTQPGADGFELQHPYVLDAGIPISQLTVCPH      180
BP2     ALLVAILPEYVIGTVAGGTGTEDSHPPYKQTQPGADGFELQHPYVLDAGIPISQLTVCPH      180
BP3     ALLVAILPEYVIGTVAGGTGTEDSHPPYKQTQPGADGFELQHPYVLDAGIPISQLTVCPH      180
BP4     ALLVAILPEYVIGTVAGGTGTEDSHPPYKQTQPGADGFELQHPYVLDAGIPISQLTVCPH      180
        ************************************************************

WT      QWINLRTNNCATIIVPYMNTLPFDSALNHCNFGLLVVPISPLDFDQGATPVIPITITLAP      240
BP1     QWINLRTNNCATIIVPYMNTLPFDSALNHCNFGLLVVPISPLDFDQGATPVIPITITLAP      240
BP2     QWINLRTNNCATIIVPYMNTLPFDSALNHCNFGLLVVPISPLDFDQGATPVIPITITLAP      240
BP3     QWINLRTNNCATIIVPYMNTLPFDSALNHCNFGLLVVPISPLDFDQGATPVIPITITLAP      240
BP4     QWINLRTNNCATIIVPYMNTLPFDSALNHCNFGLLVVPISPLDFDQGATPVIPITITLAP      240
        ************************************************************

WT      MCSEFAGLRQAVTQ   254
BP1     MCSEFAGLRQAVTQ   254
BP2     MCSEFAGLRQAVTQ   254
BP3     MCSEFAGLRQAVTQ   254
BP4     MCSEFAGLRQAVTQ   254
        **************
```

**VP3 alignment for big plaque isolates:**

```
WT      GFPTEPKPGTNQFLTTDDGVSAPILPNFHPTPCIHIPGEVRNLLELCQVETILEVNNVPT    60
BP1     GFPTEPKPGTNQFLTTDDGVSAPILPNFHPTPCIHIPGEVRNLLELCQVETILEVNNVPT    60
BP2     GFPTEPKPGTNQFLTTDDGVSAPILPNFHPTPCIHIPGEVRNLLELCQVETILEVNNVPT    60
BP3     GFPTEPKPGTNQFLTTDDGVSAPILPNFHPTPCIHIPGEVRNLLELCQVETILEVNNVPT    60
BP4     GFPTEPKPGTNQFLTTDDGVSAPILPNFHPTPCIHIPGEVRNLLELCQVETILEVNNVPT    60
        ************************************************************

WT      NATSLMERLRFPVSAQAGKGELCAVFRADPGRDGPWQSTMLGQLCGYYTQWSGSLEVTFM    120
BP1     NATSLMERLRFPVSAQAGKGELCAVFRADPGRDGPWQSTMLGQLCGYYTQWSGSLEVTFM    120
BP2     NATSLMERLRFPVSAQAGKGELCAVFRADPGRDGPWQSTMLGQLCGYYTQWSGSLEVTFM    120
BP3     NATSLMERLRFPVSAQAGKGELCAVFRADPGRDGPWQSTMLGQLCGYYTQWSGSLEVTFM    120
BP4     NATSLMERLRFPVSAQAGKGELCAVFRADPGRDGPWQSTMLGQLCGYYTQWSGSLEVTFM    120
        ************************************************************

WT      FTGSFMATGKMLIAYTPPGGPLPKDRATAMLGTHVIWDFGLQSSVTLVIPWISNTHYRAH    180
BP1     FTGSFMATGKMLIAYTPPGGPLPKDRATAMLGTHVIWDFGLQSSVTLVIPWISNTHYRAH    180
BP2     FTGSFMATGKMLIAYTPPGGPLPKDRATAMLGTHVIWDFGLQSSVTLVIPWISNTHYRAH    180
BP3     FTGSFMATGKMLIAYTPPGGPLPKDRATAMLGTHVIWDFGLQSSVTLVIPWISNTHYRAH    180
BP4     FTGSFMATGKMLIAYTPPGGPLPKDRATAMLGTHVIWDFGLQSSVTLVIPWISNTHYRAH    180
        ************************************************************

WT      ARDGVFDYYTTGLVSIWYQTNYVVPIGAPNTAYIIALAAAQKNFTMKLCKDTSHILQTAS    240
BP1     ARDGVFDYYTTGLVSIWYQTNYVVPIGAPNTAYIIALAAAQKNFTMKLCKDTSHILQTAS    240
BP2     ARDGVFDYYTTGLVSIWYQTNYVVPIGAPNTAYIIALAAAQKNFTMKLCKDTSHILQTAS    240
BP3     ARDGVFDYYTTGLVSIWYQTNYVVPIGAPNTAYIIALAAAQKNFTMKLCKDTSHILQTAS    240
BP4     ARDGVFDYYTTGLVSIWYQTNYVVPIGAPNTAYIIALAAAQKNFTMKLCKDTSHILQTAS    240
        ************************************************************

WT      IQ      242
BP1     IQ      242
BP2     IQ      242
BP3     IQ      242
BP4     IQ      242
        **
```

Alignment of sequencing results for the EV-A71/SP variant:

**VP4 alignment for all small plaque isolates:**

```
WT      MGSQVSTQRSGSHENSNSATEGSTINYTTINYYKDSYAATAGKQSLKQDPDKFANPVKDI    60
SP1     MGSQVSTQRSGSHENSNSATEGSTINYTTINYYKDSYAATAGKQSLKQDPDKFANPVKDI    60
SP2     MGSQVSTQRSGSHENSNSATEGSTINYTTINYYKDSYAATAGKQSLKQDPDKFANPVKDI    60
SP3     MGSQVSTQRSGSHENSNSATEGSTINYTTINYYKDSYAATAGKQSLKQDPDKFANPVKDI    60
SP4     MGSQVSTQRSGSHENSNSATEGSTINYTTINYYKDSYAATAGKQSLKQDPDKFANPVKDI    60
        ************************************************************

WT      FTEMAAPLK       69
SP1     FTEMAAPLK       69
SP2     FTEMAAPLK       69
SP3     FTEMAAPLK       69
SP4     FTEMAAPLK       69
        *********
```

**VP2 alignment for all small plaque isolates:**

```
WT       SPSAEACGYSDRVAQLTIGNSTITTQEAANIIVGYGEWPSYCSDDDATAVDKPTRPDVSV     60
SP1      SPSAEACGYSDRVAQLTIGNSTITTQEAANIIVGYGEWPSYCSDDDATAVDKPTRPDVSV     60
SP2      SPSAEACGYSDRVAQLTIGNSTITTQEAANIIVGYGEWPSYCSDDDATAVDKPTRPDVSV     60
SP3      SPSAEACGYSDRVAQLTIGNSTITTQEAANIIVGYGEWPSYCSDDDATAVDKPTRPDVSV     60
SP4      SPSAEACGYSDRVAQLTIGNSTITTQEAANIIVGYGEWPSYCSDDDATAVDKPTRPDVSV     60
         ************************************************************

WT       NRFYTLDTKLWEKSSKGWYWKFPDVLTETGVFGQNAQFHYLYRSGFCIHVQCNASKFHQG     120
SP1      NRFYTLDTKLWEKSSKGWYWKFPDVLTETGVFGQNAQFHYLYRSGFCIHVQCNASKFHQG     120
SP2      NRFYTLDTKLWEKSSKGWYWKFPDVLTETGVFGQNAQFHYLYRSGFCIHVQCNASKFHQG     120
SP3      NRFYTLDTKLWEKSSKGWYWKFPDVLTETGVFGQNAQFHYLYRSGFCIHVQCNASKFHQG     120
SP4      NRFYTLDTKLWEKSSKGWYWKFPDVLTETGVFGQNAQFHYLYRSGFCIHVQCNASKFHQG     120
         ************************************************************

WT       ALLVAILPEYVIGTVAGGTGTEDSHPPYKQTQPGADGFELQHPYVLDAGIPISQLTVCPH     180
SP1      ALLVAILPEYVIGTVAGGTGTEDSHPPYKQTQPGADGFELQHPYVLDAGIPISQLTVCPH     180
SP2      ALLVAILPEYVIGTVAGGTGTEDSHPPYKQTQPGADGFELQHPYVLDAGIPISQLTVCPH     180
SP3      ALLVAILPEYVIGTVAGGTGTEDSHPPYKQTQPGADGFELQHPYVLDAGIPISQLTVCPH     180
SP4      ALLVAILPEYVIGTVAGGTGTEDSHPPYKQTQPGADGFELQHPYVLDAGIPISQLTVCPH     180
         ************************************************************

WT       QWINLRTNNCATIIVPYMNTLPFDSALNHCNFGLLVVPISPLDFDQGATPVIPITITLAP     240
SP1      QWINLRTNNCATIIVPYMNTLPFDSALNHCNFGLLVVPISPLDFDQGATPVIPITITLAP     240
SP2      QWINLRTNNCATIIVPYMNTLPFDSALNHCNFGLLVVPISPLDFDQGATPVIPITITLAP     240
SP3      QWINLRTNNCATIIVPYMNTLPFDSALNHCNFGLLVVPISPLDFDQGATPVIPITITLAP     240
SP4      QWINLRTNNCATIIVPYMNTLPFDSALNHCNFGLLVVPISPLDFDQGATPVIPITITLAP     240
         ************************************************************

WT       MCSEFAGLRQAVTQ   254
SP1      MCSEFAGLRQAVTQ   254
SP2      MCSEFAGLRQAVTQ   254
SP3      MCSEFAGLRQAVTQ   254
SP4      MCSEFAGLRQAVTQ   254
         **************
```

**VP3 alignment for all small plaque isolates:**

```
WT       GFPTEPKPGTNQFLTTDDGVSAPILPNFHPTPCIHIPGEVRNLLELCQVETILEVNNVPT     60
SP1      GFPTEPKPGTNQFLTTDDGVSAPILPNFHPTPCIHIPGEVRNLLELCQVETILEVNNVPT     60
SP2      GFPTEPKPGTNQFLTTDDGVSAPILPNFHPTPCIHIPGEVRNLLELCQVETILEVNNVPT     60
SP3      GFPTEPKPGTNQFLTTDDGVSAPILPNFHPTPCIHIPGEVRNLLELCQVETILEVNNVPT     60
SP4      GFPTEPKPGTNQFLTTDDGVSAPILPNFHPTPCIHIPGEVRNLLELCQVETILEVNNVPT     60
         ************************************************************

WT       NATSLMERLRFPVSAQAGKGELCAVFRADPGRDGPWQSTMLGQLCGYYTQWSGSLEVTFM     120
SP1      NATSLMERLRFPVSAQAGKGELCAVFRADPGRDGPWQSTMLGQLCGYYTQWSGSLEVTFM     120
SP2      NATSLMERLRFPVSAQAGKGELCAVFRADPGRDGPWQSTMLGQLCGYYTQWSGSLEVTFM     120
SP3      NATSLMERLRFPVSAQAGKGELCAVFRADPGRDGPWQSTMLGQLCGYYTQWSGSLEVTFM     120
SP4      NATSLMERLRFPVSAQAGKGELCAVFRADPGRDGPWQSTMLGQLCGYYTQWSGSLEVTFM     120
         ************************************************************

WT       FTGSFMATGKMLIAYTPPGGPLPKDRATAMLGTHVIWDFGLQSSVTLVIPWISNTHYRAH     180
SP1      FTGSFMATGKMLIAYTPPGGPLPKDRATAMLGTHVIWDFGLQSSVTLVIPWISNTHYRAH     180
SP2      FTGSFMATGKMLIAYTPPGGPLPKDRATAMLGTHVIWDFGLQSSVTLVIPWISNTHYRAH     180
SP3      FTGSFMATGKMLIAYTPPGGPLPKDRATAMLGTHVIWDFGLQSSVTLVIPWISNTHYRAH     180
SP4      FTGSFMATGKMLIAYTPPGGPLPKDRATAMLGTHVIWDFGLQSSVTLVIPWISNTHYRAH     180
         ************************************************************

WT       ARDGVFDYYTTGLVSIWYQTNYVVPIGAPNTAYIIALAAAQKNFTMKLCKDTSHILQTAS     240
SP1      ARDGVFDYYTTGLVSIWYQTNYVVPIGAPNTAYIIALAAAQKNFTMKLCKDTSHILQTAS     240
SP2      ARDGVFDYYTTGLVSIWYQTNYVVPIGAPNTAYIIALAAAQKNFTMKLCKDTSHILQTAS     240
SP3      ARDGVFDYYTTGLVSIWYQTNYVVPIGAPNTAYIIALAAAQKNFTMKLCKDTSHILQTAS     240
SP4      ARDGVFDYYTTGLVSIWYQTNYVVPIGAPNTAYIIALAAAQKNFTMKLCKDTSHILQTAS     240
         ************************************************************

WT       IQ       242
SP1      IQ       242
SP2      IQ       242
SP3      IQ       242
SP4      IQ       242
         **
```

Supplementary Figure S2: Raw genome sequencing data of EV-A71/BP and EV-A71/SP.

**>Wild type parental sequence 15 reads de novo assembled using Geneious Software**

GGCTGTGGGTTGTACCCACTCACAGGGCCCACGTGGCGCTAGCACTCTGGTTCTGCGGAACCTTTGTGCGCCTGTTTTACGCCCCCCCCCCAATTTGCAA
CTTAGAAGCAATACACAACACTGATCAACAGCAGGCATGGCGCACCAGCTATGTCTTGATCAAGCACTTCTGTTTCCCCGGGCCGAGTATCAATAGACTG
TTCACGCGGTTGAAGGAGAAAGCGCCCGTTATCCGGCTAACTACTTCGAGAAACCTAGTAGCACCATTGAAGCTGCAGAGTGTTTCGCTCGGCACTTCCC
CCGTGTAGATCAGGTCGATGAGTCACTGCAATCCCCACGGGCGACCGTGGCAGTGGCTGCGCTGGCGGCCTGCCTATGGGGCAACCCATAGGACGCTCTA
ATGTGGACATGGTGCGAAGAGTCTATTGAGCTAGTTAGTAGTCCTCCGGCCCCTGAATGCGGCTAATCCTAACTGTGGAGCACATGCCTTCAATCCAGAG
GGTAGTGTGTCGTAATGGGCAACTCTGCAGCGGAACCGACTACTTTGGGTGTCCGTGTTTCCTTTTATCTTTACATTGGCTGCTTATGGTGACGATTATA
GAATTGTTACCATATAGCTATTGGATTGGCCATCCGGTGTGCAATAGAGCTATTATATACCTGTTTGTTGGCTTTGTACCACTAACCTTAAAATCTATAA
CCACCCTCGATTTTATATTAACCCTCAATACAATCAAACATGGGCTCACAGGTGTCTACTCAGCGATCCGGCTCCCACGAGAACTCCAATTCAGCTACAG
AAGGCTCCACCATTAATTACACTACCATCAACTATTACAAAGACTCCTATGCTGCGACAGCGGGCAAACAGAGCCTCAAGCAAGACCCTGATAAATTTGC
TAACCCTGTCAAGGACATTTTCACTGAAATGGCTGCACCACTGAAGTCTCCATCCGCTGAGGCTTGTGGTTACAGTGATCGCGTGGCACAACTCACCATT
GGAAACTCCACCATCACTACACAGGAGGCGGCGAATATCATAGTCGGTTATGGTGAGTGGCCCTCATACTGCTCTGATGACGATGCTACAGCGGTGGACA
AGCCAACGCGCCCAGATGTTTCAGTGAATAGGTTTTATACGTTGGATACTAAATTGTGGGAAAAGTCATCCAAGGGGTGGTATTGGAAGTTTCCTGATGT
ACTGACTGAGACCGGAGTCTTTGGCCAGAATGCACAGTTTCACTATTTATATAGGTCAGGATTTTGCATTCATGTGCAATGTAATGCTAGCAAGTTCCAT
CAAGGAGCGTTGTTAGTCGCCATACTTCCAGAGTATGTTATAGGGACAGTGGCAGGCGGCACAGGAACTGAGGACAGCCACCCTCCTTACAAACAAACAC
AACCTGGCGCCGATGGTTTTGAGTTGCAGCACCCGTACGTACTCGATGCTGGGATTCCTATATCACAATTAACAGTGTGCCCCCACCAATGGATTAACCT
ACGGACCAATAACTGTGCCACAATAATAGTGCCGTATATGAACACACTGCCTTTCGACTCTGCCCTGAACCATTGCAACTTTGGGCTGTTGGTGGTGCCC
ATTAGCCCACTAGATTTTGACCAAGGGGCAACTCCGGTTATCCCTATTACAATCACTCTAGCTCCAATGTGCTCTGAGTTTGCAGGTCTCAGACAGGCGG
TCACTCAAGGTTTTCCCACCGAGCCAAAACCAGGAACGAATCAATTTTTGACCACCGATGACGGTGTCTCAGCACCCATTTTACCAAATTTCCACCCCAC
ACCATGTATTCACATACCCGGTGAAGTCAGAAACCTGCTTGAGTTGTGTCAAGTGGAGACCATTCTTGAGGTTAACAATGTACCCACCAATGCCACCAGT
CTGATGGAAAGGCTACGATTCCCGGTGTCCGCGCAAGCGGGAAAAGGTGAATTGTGTGCCGTGTTTAGGGCCGACCCTGGAAGAGACGGTCCATGGCAAT
CAACAATGCTGGGCCAGTTGTGTGGATATTACACCCAGTGGTCAGGATCACTGGAGGTTACTTTTATGTTCACCGGGTCTTTCATGGCCACGGGTAAAAT
GCTCATAGCTTATACACCTCCTGGTGGCCCCTTACCCAAAGATCGGGCCACAGCAATGCTGGGCACACATGTTATCTGGGATTTTGGGCTACAATCATCT
GTCACCCTTGTAATACCATGGATTAGCAACACCCACTACAGAGCGCATGCCCGGGATGGAGTGTTCGATTACTATACCACAGGACTGGTTAGTATCTGGT
ATCAAACAAACTACGTGGTTCCAATTGGGGCACCCAACACAGCTTACATAATAGCACTAGCGGCAGCCCAGAAGAATTTTACCATGAAACTGTGTAAAGA
CACCAGTCACATATTACAGACAGCCTCTATTCAGGGAGATAGAGTGGCAGATGTGATAGAGAGCTCTATAGGAGATAGTGTGAGTAGGGCACTTACCCAG
GCCCTGCCAGCTCCAACAGGTCAGAACACGCAGGTGAGCAGTCATCGACTAGACACTGGTGAAGTTCCAGCGCTCCAAGCTGCTGAAATAGGGGCATCGT
CAAATACTAGTGATGAGAGTATGATTGAGACACGATGCGTTCTTAATTCACACAGTACGGCAGAGACCACCCTGGACAGCTTCTTCAGTAGGGCAGGCTT
GGTAGGAGAGATAGATCTCCCTATTGAGGGTACCACTAATCCAAATGGTTATGCTAATTGGGATATAGACATAACTGGTTACGCACAAATGCGCAGGAAA
GTGGAGCTGTTCACCTACATGCGCTTTGATGCGGAATTCACTTTTGTTGCGTGCACTCCTACTGGTCAGGTTGTCCCACAATTACTTCAGTATATGTTTG
TTCCCCCTGGTGCTCCCAAACCAGAGTCTAGAGAATCACTTGCTTGGCAGACAGCCACAAACCCCTCAGTTTTTGTCAAGTTGACTGATCCCCCGGCACA
GGTCTCAGTTCCGTTCATGTCACCCGCGAGCGCTTACCAGTGGTTTTACGACGGGTACCCCACGTTTGGAGAACACAAACAGGAGAAAGACCTTGAGTAT
GGAGCGTGCCCTAATAATATGATGGGCACTTTCTCGGTGCGAAATGTGGGTTCATCAAAGTCCAAGTATYCTTTGGTTGTCAGGATATATATGAGAATGA
AGCATGTCAGGGCGTGGATACCTCGCCCGATGCGCAACCAAAACTACCTGTTTAAAGCCAATCCAAACTATGCCGGTAACTCCATCAAACCGACCGGCAC
TAGTCGTACTGCCATTACTACCCTTGGAAAGTTCGGCCAGCAATCTGGGGCCATCTACGTGGGCAACTTCAGAGTGGTTAATCGTCACCTCGCTACTCAT
AATGACTGGGCGAACCTCGTCTGGGAAGATAGCTCCCGCGACCTATTAGTGTCGTCTACCACCGCCCAGGGCTGTGATACAATTGCACGTTGTGACTGTC
AAACAGGAGTGTACTATTGTAATTCCAAAAGAAAGCACTATCCAGTCAGCTTCTCCAAACCCAGCCTCATATATGTGGAGGCTAGCGAGTATTACCCTGC
TAGATACCAATCGCACCTGATGCTTGCAGCAGGCCACTCTGAGCCCGGCGACTGCGGGGGCATCTTAAGGTGTCAACATGGTGTAGTTGGTATAGTGTCC
ACGGGTGGCAACGGGCTCGTTGGTTTTGCTGATGTGAGGGATCTCTTGTGGTTGGATGAAGAGGCCATGGAGCAAGGTGTGTCTGACTACATTAAGGGGC
TCGGTGACGCATTTGGAACAGGTTTCACTGATGCTGTATCCAGGGAAGTTGAAGCCCTCAGGAACCACCTCATAGGATCTGATGGAGCAGTGGAAAAAAT
CCTAAAGAACCTTATTAAGCTGATTTCAGCGTTAGTAATTGTGATTAGGAGCGATTATGATATGGTCACCCTCACAGCAACTTTAGCCCTGATTGGTTGT
CATGGAAGTCCCTGGGCTTGGATTAAAGCCAAAACAGCATCCATTTTAGGTATCCCCATCGCCCAGAAGCAGAGCGCTTCTTGGCTAAAGAAATTTAATG
ATATGGCGAGTGCTGCCAAGGGTTTAGAATGGATATCCAACAAAATTAGTAAGTTCATTGACTGGCTCAGGGAGAAGATTGTTCCAGCAGCTAAAGAGAA
AGCAGAATTTTTAACCAATTTGAAGCAATTACCACTATTAGAGAACCAGATCACGAACTTGGAGCAGTCCGCTGCCTCGCAAGAGGACCTTGAAGCTATG
TTTGGGAATGTGTCATACCTCGCCCATTTCTGTCGCAAGTTCCAACCATTATACGCCACGGAGGCCAAGCGAGTCTATGTTCTAGAGAAGAGAATGAACA
ATTACATGCAGTTCAAGAGCAAACACCGTATTGAACCTGTATGTCTCATCATTAGAGGCTCACCAGGCACTGGAAAGTCCCTTGCGACCGGCATCATTGC
CCGGGCCATAGCAGACAAGTACCACTCTAGTGTGTACTCACTCCCACCGGATCCTGACCATTTTGACGGGTACAAACAGCAAGTGGTTACAGTTATGGAT
GACCTGTGCCAGAATCCTGACGGCAAAGACATGTCATTATTTTGCCAGATGGTATCCACCGTGGATTTTATTCCACCAATGGCTTCTCTCGAAGAAAAGG
GAGTTTCTTTCACATCTAAATTTGTTATCGCATCCACCAACGCCAGCAACATTATAGTGCCCACAGTGTCTGACTCTGACGCCATTCGTCGCAGGTTCTA
CATGGATTGCGACATTGAGGTCACAGACTCATACAAAACAGACTTGGGTAGACTAGACGCTGGGCGGGCTGCTAAGTTATGCTCTGAAAACAACACCGCA
AATTTCAAACGATGCAGCCCACTAGTGTGTGGGAAAGCTATTCAACTTAGAGACAGGAAATCCAAGGTCAGGTATAGCGTGGACACAGTGGTCTCTGAAC
TTATTAGAGAATACAATAGCAGATCCGCTATTGGTAACACAATTGAAGCATTATTCCAAGGCCCACCCAAGTTCAGGCCAATAAGGATCAGTCTTGAGGA
GAAGCCAGCCCCAGACGCTATTAGCGATCTCCTTGCTAGTGTGGATAGCGAGGAAGTGCGCCAATACTGTAGGGAACAAGGCTGGATTATCCCTGAAACT
CCCACCAATGTTGAACGACATCTTAATAGAGCAGTGCTAGTCGTGCAATCCATCACTACTGTGGTGGCAGTCGTCTCACTGGTGTACGTCATTTACAAGC
TCTTTGCGGGGTTTCAAGGTGCGTATTCTGGAGCTCCCAAGCAAGTGCTCAAGAAGCCTGTCCTCCGCACGGCAACAGTGCAGGGTCCAAGCCTTGATTT
TGCCCTATCCTTGCTGAGGAGGAACATCAGGCAAGTCCAAACAGACCAAGGGCATTTTACCATGTTGGGTGTCAGGGATCGCCTGGCTGTTCTCCCGCGG
CACTCACAGCCCGGGAAGACTATTTGGGTGGAACACAAACTTGTGAACATCCTTGATGCAGTCGAGCTGGTGGACGAGCAGGGCGTTAATTTGGAACTCA
CATTGGTGACACTAGATATTAATGAAAAATTTAGAGATATCACCAAGTTCATTCCAGAGACCATTAGCGGCGCTAGTGATGCAACTCTAGTGATCAACAC
AGAACATATGCCGTCAATGTTTGTCCCTGTGGGGGACGTCGTGCAGTACGGGTTCTTGAACCTCAGTGGAAAGCCAACACATAGGACCATGATGTACAAT
TTCCCTACAAAAGCAGGACAGTGTGGAGGCGTGGTTACATCAGTCGGTAAGATTGTTGGTATTCACATTGGTGGCAACGGGCGCCAAGGGTTCTGCGCTG
GTTTGAAGAGGAGCTACTTTGCGAGTATGCAAGGTGAGATCCAATGGGTGAAGCCTAACAAGGAAACTGGCAGACTAAACATCAATGGACCAACTCGCAC
TAAGTTGGAGCCTAGTGTATTTCATGATGTGTTTGAAGGCAACAAGGAACCAGCAGTTTTAACAAGTAAAGACCCTAGATTGGAGGTCGACTTTGAACAA
GCCCTGTTTTCCAAGTATGTGGGCAATGTTTTACACGAGCCCGATGAATATGTGACTCAAGCTGCCCTCCACTATGCGAATCAACTTAAACAATTGGACA
TAAACACTAGCAAGATGAGCATGGAGGAAGCGTGCTATGGCACTGAAAACCTGGAAGCAATAGACCTCTGCACTAGTGCTGGGTATCCATACAGTGCCCT
TGGTATCAAGAAAAGAGACATTCTCGACCCCATAACCAGGGATGTGTCTAAGATGAAATTCTACATGGATAAATACGGACTAGATCTGCCATACTCTACC
TATGTGAAGGATGAACTTAGATCTCTGGATAAAATCAAGAAAGGAAAGTCACGCCTGATAGAGGCCAGCAGCTTGAATGACTCTGTCTACCTCAGAATGA
CTTTTGGGCACCTTTACGAGGTGTTTCATGCTAACCCTGGTACTGTGACTGGCTCAGCAGTAGGTTGCAACCCAGACGTGTTTTGGAGTAAACTACCGAT
TCTGCTGCCTGGGTCACTCTTTGCCTTTGACTACTCAGGATATGATGCTAGTCTCAGCCCGGTATGGTTCAGGGCTCTAGAAGTTGTGTTACGGGAGATT
GGGTATTCAGAGGAGGCCGTGTCCCTAATAGAAGGAATCAACCACACCCACCATGTGTACCGGAATAAAACATACTGTGTACTTGGTGGGATGCCCTCAG
GGTGCTCTGGTACTTCCATCTTCAATTCAATGATCAACAACATCATCATTAGAACCCTTTTGATCAAAACCTTTAAGGGAATAGACCTGGATGAGTTGAA
CATGGTGGCCTATGGGGACGATGTGCTGGCCAGTTACCCTTTTCCTATTGATTGCCTTGAATTGGCTAAGACTGGCAAAGAGTATGGTTTGACCATGACT
CCTGCAGACAAATCACCCTGTTTCAATGAAGTAACATGGGAGAATGCTACCTTCCTGAAGAGAGGGTTCTTGCCAGACCACCAATTTCCATTCTTAATTC
ACCCTACGATGCCCATGAGAGAGATCCATGAGTCCATTCGATGGACTAAGGACGCGCGTAACACCCAGGATCACGTGCGCTCCCTGTGTCTATTGGCATG
GCACAATGGTAAGGATGAATATGAAAAGTTTGTGAGTGCAATTAGATCAGTTCCAGTTGGAAAAGCGTTGGCCATTCCTAACTTTGAGAATCTGAGAAGA
AATTGGCTYGAATTGTTTTAATATTACAGYTTAAAGCTGAACCCCACTAGRARKSTGKTYKTTTTTTTCCCCCCAAAAAAAACACA

## >EV-A71_small plaque isolate (SP1) DE NOVO ASSEMBLY

GCGTGGGTTGTACCCACTCACAGGGCCCACGTGGCGCTAGCACTCTGGTTCTGCGGAACCTTTGTGCGCCTGTTTTACGCCCCCCCCCCAATTTGCAACT
TAGAAGCAATACACAACACTGATCAACAGCAGGCATGGCGCACCAGCTATGTCTTGATCAAGCACTTCTGTTTCCCCGGGCCGAGTATCAATAGACTGTT
CACGCGGTTGAAGGAGAAAGCGCCCGTTATCCGGCTAACTACTTCGAGAAACCTAGTAGCACCATTGAAGCTGCAGAGTGTTTCGCTCGGCACTTCCCCC
GTGTAGATCAGGTCGATGAGTCACTGCAATCCCCACGGGCGACCGTGGCAGTGGCTGCGCTGGCGGCCTGCCTATGGGGCAACCCATAGGACGCTCTAAT
GTGGACATGGTGCGAAGAGTCTATTGAGCTAGTTAGTAGTCCTCCGGCCCCTGAATGCGGCTAATCCTAACTGTGGAGCACATGCCTTCAATCCAGAGGG
TAGTGTGTCGTAATGGGCAACTCTGCAGCGGAACCGACTACTTTGGGTGTCCGTGTTTCCTTTTATCTTTACATTGGCTGCTTATGGTGACGATTATAGA
ATTGTTACCATATAGCTATTGGATTGGCCATCCGGTGTGCAATAGAGCTATTATATACCTGTTTGTTGGCTTTGTACCACTAACCTTAAAATCTATAACC
ACCCTCGATTTTATATTAACCCTCAATACAATCAAACATGGGCTCACAGGTGTCTACTCAGCGATCCGGCTCCCACGAGAACTCCAATTCGGCTACAGAA
GGCTCCACCATTAATTACACTACCATCAACTATTACAAAGACTCCTATGCTGCGACAGCGGGCAAACAGAGCCTCAAGCAAGACCCTGATAAATTTGCTA
ACCCTGTCAAGGACATTTTCACTGAAATGGCTGCACCACTGAAGTCTCCATCCGCTGAGGCTTGTGGTTACAGTGATCGCGTGGCACAACTCACCATTGG
AAACTCCACCATCACTACACAGGAGGCGGCGAATATCATAGTCGGTTATGGTGAGTGGCCCTCATACTGCTCTGATGACGATGCTACAGCGGTGGACAAG
CCAACGCGCCCAGACGTTTCAGTGAATAGGTTTTATACGTTGGATACTAAATTGTGGGAAAAGTCATCCAAGGGGTGGTATTGGAAGTTTCCTGATGTAC
TGACTGAGACCGGAGTCTTTGGCCAGAATGCACAGTTTCACTATTTATATAGGTCAGGATTTTGCATTCATGTGCAATGTAATGCTAGCAAGTTCCATCA
AGGAGCGTTGTTAGTCGCCATACTTCCAGAGTATGTTATAGGGACAGTGGCAGGCGGCACAGGAACTGAGGACAGCCACCCTCCTTACAAACAAACACAA
CCTGGCGCCGATGGTTTTGAGTTGCAGCACCCGTACGTACTCGATGCTGGGATTCCTATATCACAATTAACAGTGTGCCCCCACCAATGGATTAACCTAC
GGACCAATAACTGTGCCACAATAATAGTGCCGTATATGAACACACTGCCTTTCGACTCTGCCCTGAACCATTGCAACTTTGGGCTGTTGGTGGTGCCCAT
TAGCCCACTAGATTTTGACCAAGGGGCAACTCCGGTTATCCCTATTACAATCACTCTAGCTCCAATGTGCTCTGAGTTTGCAGGTCTCAGACAGGCGGTC
ACTCAAGGTTTTCCCACCGAGCCAAAACCAGGAACGAATCAATTTTTGACCACCGATGACGGTGTCTCAGCACCCATTTTACCAAATTTCCACCCCACAC
CATGTATTCACATACCCGGTGAAGTCAGAAACCTGCTTGAGTTGTGTCAAGTGGAGACCATTCTTGAGGTTAACAATGTACCCACCAATGCCACCAGTCT
GATGGAAAGGCTACGATTCCCGGTGTCCGCGCAAGCGGGAAAAGGTGAATTGTGTGCCGTGTTTAGGGCCGACCCTGGAAGAGACGGTCCATGGCAATCA
ACAATGCTGGGCCAGTTGTGTGGATATTACACCCAGTGGTCAGGATCACTGGAGGTTACTTTTATGTTCACCGGGTCTTTCATGGCCACGGGTAAAATGC
TCATAGCTTATACACCTCCTGGTGGCCCCTTACCCAAAGATCGGGCCACAGCAATGCTGGGCACACATGTTATCTGGGATTTTGGGCTACAATCATCTGT
CACCCTTGTAATACCATGGATTAGCAACACCCACTACAGAGCGCATGCCCGGGATGGAGTGTTCGATTACTATACCACAGGACTGGTTAGTATCTGGTAT
CAAACAAACTACGTGGTTCCAATTGGGGCACCCAACACAGCTTACATAATAGCACTAGCGGCAGCCCAGAAGAATTTTACCATGAAACTGTGTAAAGACA
CCAGTCACATATTACAGACAGCCTCTATTCAGGGAGATAGAGTGGCAGATGTGATAGAGAGCTCTATAGGAGATAGTGTGAGTAGGGCACTTACCCAGGC
CCTGCCAGCTCCAACAGGTCAGAACACGCAGGTGAGCAGTCATCGACTAGACACTGGTGAAGTTCCAGCGCTCCAAGCTGCTGAAATAGGGGCATCGTCA
AATACTAGTGATGAGAGTATGATTGAGACACGATGCGTTCTTAATTCACACAGTACGGCAGAGACCACCCTGGACAGCTTCTTCAGTAGGGCAGGCTTGG
TAGGAGAGATAGATCTCCCTACTGAGGGTACCACTAATCCAAATGGTTATGCTAATTGGGATATAGACATAACTGGTTACGCACAAATGCGCAGGAAAGT
GGAGCTGTTCACCTACATGCGCTTTGATGCGGAATTCACTTTTGTTGCGTGCACTCCTACTGGTCAGGTTGTCCCACAATTACTTCAGTATATGTTTGTT
CCCCCTGGTGCTCCCAAACCAGAGTCTAGAGAATCACTTGCTTGGCAGACAGCCACAAACCCCTCAGTTTTTGTCAAGTTGACTGATCCCCCGGCACAGG
TCTCAGTTCCGTTCATGTCACCCGCGAGCGCTTACCAGTGGTTTTACGACGGGTACCCCACGTTTGGAGAACACAAACAGGAGAAAGACCTTGAGTATGG
AGCGTGCCCTAATAATATGATGGGCACTTTCTCGGTGCGAACTGTGGGTTCATCAAAGTCCAAGTATTCTTTGGTTGTCAGGATATATATGAGAATGAAG
CATGTCAGGGCGTGGATACCTCGCCCGATGCGCAACCAAAACTACCTGTTTAAAGCCAATCCAAACTATGCCGGTAACTCCATCAAACCGACCGGCACTA
GTCGTGCTGCCATTACTACCCTTGGAAAGTTCGGCCAGCAATCTGGGGCCATCTACGTGGGCAACTTCAGAGTGGTTAATCGTCACCTCGCTACTCATAA
TGACTGGGCGAACCTCGTCTGGGAAGATAGCTCCCGCGACCTATTAGTGTCGTCTACCACCGCCCAGGGCTGTGATACAATTGCACGTTGTGACTGTCAA
ACAGGAGTGTACTATTGTAATTCCAAAAGAAAGCACTATCCAGTCAGCTTCTCCAAACCCAGCCTCATATATGTGGAGGCTAGCGAGTATTACCCTGCTA
GATACCAATCGCACCTGATGCTTGCAGCAGGCCACTCTGAGCCCGGCGACTGCGGGGGCATCTTAAGGTGTCAACATGGTGTAGTTGGTATAGTGTCCAC
GGGTGGCAACGGGCTCGTTGGTTTTGCTGATGTGAGGGATCTCTTGTGGTTGGATGAAGAGGCCATGGAGCAAGGTGTGTCTGACTACATTAAGGGGCTC
GGTGACGCATTTGGAACAGGTTTCACTGATGCTGTATCCAGGGAAGTTGAAGCCCTCAGGAACCACCTCATAGGATCTGATGGAGCAGTGGAAAAAATCC
TAAAGAACCTTATTAAGCTGATTTCAGCGTTAGTAATTGTGATTAGGAGCGATTATGATATGGTCACCCTCACAGCAACTTTAGCCCTGATTGGTTGTCA
TGGAAGTCCCTGGGCTTGGATTAAAGCCAAAACAGCATCCATTTTAGGTATCCCCATCGCCCAGAAGCAGAGCGCTTCTTGGCTAAAGAAATTTAATGAT
ATGGCGAGTGCTGCCAAGGGTTTAGAATGGATATCCAACAAAATTAGTAAGTTCATTGACTGGCTCAGGGAGAAGATTGTTCCAGCAGCTAAAGAGAAAG
CAGAATTTTTAACCAATTTGAAGCAATTACCACTATTAGAGAACCAGATCACGAACTTGGAGCAGTCCGCTGCCTCGCAAGAGGACCTTGAAGCTATGTT
TGGGAATGTGTCATACCTCGCCCATTTCTGTCGCAAGTTCCAACCATTATACGCCACGGAGGCCAAGCGAGTCTATGTTCTAGAGAAGAGAATGAACAAT
TACATGCAGTTCAAGAGCAAACACCGTATTGAACCTGTATGTCTCATCATTAGAGGCTCACCAGGCACTGGAAAGTCCCTTGCGACCGGCATCATTGCCC
GGGCCATAGCAGACAAGTACCACTCTAGTGTGTACTCACTCCCACCGGATCCTGACCATTTTGACGGGTACAAACAGCAAGTGGTTACAGTTATGGATGA
CCTGTGCCAGAATCCTGACGGCAAAGACATGTCATTATTTTGCCAGATGGTATCCACCGTGGATTTTATTCCACCAATGGCTTCTCTCGAAGAAAAGGGA
GTTTCTTTCACATCTAAATTTGTTATCGCATCCACCAACGCCAGCAACATTATAGTGCCCACAGTGTCTGACTCTGACGCCATTCGTCGCAGGTTCTACA
TGGATTGCGACATTGAGGTCACAGACTCATACAAAACAGACTTGGGTAGACTAGACGCTGGGCGGGCTGCTAAGTTATGCTCTGAAAACAACACCGCAAA
TTTCAAACGATGCAGCCCACTAGTGTGTGGGAAAGCTATTCAACTTAGAGACAGGAAATCCAAGGTCAGGTATAGCGTGGACACAGTGGTCTCTGAACTT
ATTAGAGAATACAATAGCAGATCCGCTATTGGTAACACAATTGAAGCATTATTCCAAGGCCCACCCAAGTTCAGGCCAATAAGGATCAGTCTTGAGGAGA
AGCCAGCCCCAGACGCTATTAGCGATCTCCTTGCTAGTGTGGATAGCGAGGAAGTGCGCCAATACTGTAGGGAACAAGGCTGGATTATCCCTGAAACTCC
CACCAATGTTGAACGACATCTTAATAGAGCAGTGCTAGTCGTGCAATCCATCACTACTGTGGTGGCAGTCGTCTCACTGGTGTACGTCATTTACAAGCTC
TTTGCGGGGTTTCAAGGTGCGTATTCTGGAGCTCCCAAGCAAGTGCTCAAGAAGCCTGTCCTCCGCACGGCAACAGTGCAGGGTCCAAGCCTTGATTTTG
CCCTATCCTTGCTGAGGAGGAACATCAGGCAAGTCCAAACAGACCAAGGGCATTTTACCATGTTGGGTGTCAGGGATCGCCTGGCTGTTCTCCCGCGGCA
CTCACAGCCCGGGAAGACTATTTGGGTGGAACACAAACTTGTGAACATCCTTGATGCAGTCGAGCTGGTGGACGAGCAGGGCGTTAATTTGGAACTCACA
TTGGTGACACTAGATATTAATGAAAAATTTAGAGATATCACCAAGTTCATTCCAGAGACCATTAGCGGCGCTAGTGATGCAACTCTAGTGATCAACACAG
AACATATGCCGTCAATGTTTGTCCCTGTGGGGGACGTCGTGCAGTACGGGTTCTTGAACCTCAGTGGAAAGCCAACACATAGGACCATGATGTACAATTT
CCCTACAAAAGCAGGACAGTGTGGAGGCGTGGTTACATCAGTCGGTAAGATTGTTGGTATTCACATTGGTGGCAACGGGCGCCAAGGGTTCTGCGCTGGT
TTGAAGAGGAGCTACTTTGCGAGTATGCAGGGTGAGATCCAATGGGTGAAGCCTAACAAGGAAACTGGCAGACTAAACATCAATGGACCAACTCGCACTA
AGTTGGAGCCTAGTGTATTTCATGATGTGTTTGAAGGCAACAAGGAACCAGCAGTTTTAACAAGTAAAGACCCTAGATTGGAGGTCGACTTTGAACAAGC
CCTGTTTTCCAAGTATGTGGGCAATGTTTTACACGAGCCCGATGAATATGTGACTCAAGCTGCCCTCCACTATGCGAATCAACTTAAACAATTGGACATA
AACACTAGCAAGATGAGCATGGAGGAAGCGTGCTATGGCACTGAAAACCTGGAAGCAATAGACCTCTGCACTAGTGCTGGGTATCCATACAGTGCCCTTG
GTATCAAGAAAAGAGACATTCTCGACCCCATAACCAGGGATGTGTCTAAGATGAAATTCTACATGGATAAATACGGACTAGATCTGCCATACTCTACCTA
TGTGAAGGATGAACTTAGATCTCTGGATAAAATCAAGAAAGGAAAGTCACGCCTGATAGAGGCCAGCAGCTTGAATGACTCTGTCTACCTCAGAATGACT
TTTGGGCACCTTTACGAGGTGTTTCATGCTAACCCTGGTACTGTGACTGGCTCAGCAGTAGGTTGCAACCCAGACGTGTTTTGGAGTAAACTACCGATTC
TGCTGCCTGGGTCACTCTTTGCCTTTGACTACTCAGGATATGATGCTAGTCTCAGCCCGGTATGGTTCAGGGCTCTAGAAGTTGTGTTACGGGAGATTGG
GTATTCGGAGGAGGCCGTGTCCCTAATAGAAGGAATCAACCACACCCACCATGTGTACCGGAATAAAACATACTGTGTACTTGGTGGGATGCCCTCAGGG
TGCTCTGGTACTTCCATCTTCAATTCAATGATCAACAACATCATCATTAGAACCCTTTTGATCAAAACCTTTAAGGGAATAGACCTGGATGAGTTGAACA
TGGTGGCCTATGGGGACGATGTGCTGGCCAGTTACCCTTTTCCTATTGATTGCCTTGAATTGGCTAAGACTGGCAAAGAGTATGGTTTGACCATGACTCC
TGCAGACAAATCACCCTGTTTCAATGAAGTAACATGGGAGAATGCTACCTTCCTGAAGAGAGGGTTCTTGCCAGACCACCAATTTCCATTCTTAATTCAC
CCTACGATGCCCATGAGAGAGATCCATGAGTCCATTCGATGGACTAAGGACGCGCGTAACACCCAGGATCACGTGCGCTCCCTGTGTCTATTGGCATGGC
ACAATGGTAAGGATGAATATGAAAAGTTTGTGAGTGCAATTAGATCAGTTCCAGTTGGAAAAGCGTTGGCCATTCCTAACTTTGAGAATCTGAGAAGAAA
TTGGCTCGAATTGTTTTAATATTACAGTTTAAAGCTGAACCCCACTAGAAATCTGGTCG

## >EV-A71_small plaque isolate (SP2) DE NOVO ASSEMBLY

GCTGTGGGTTGTACCCACTCACAGGGCCCACGTGGCGCTAGCACTCTGGTTCTGCGGAACCTTTGTGCGCCTGTTTTACGCCCCCCCCCCAATTTGCAAC
TTAGAAGCAATACACAACACTGATCAACAGCAGGCATGGCGCACCAGCTATGTCTTGATCAAGCACTTCTGTTTCCCCGGGCCGAGTATCAATAGACTGT
TCACGCGGTTGAAGGAGAAAGCGCCCGTTATCCGGCTAACTACTTCGAGAAACCTAGTAGCACCATTGAAGCTGCAGAGTGTTTCGCTCGGCACTTCCCC
CGTGTAGATCAGGTCGATGAGTCACTGCAATCCCCACGGGCGACCGTGGCAGTGGCTGCGCTGGCGGCCTGCCTATGGGGCAACCCATAGGACGCTCTAA
TGTGGACATGGTGCGAAGAGTCTATTGAGCTAGTTAGTAGTCCTCCGGCCCCTGAATGCGGCTAATCCTAACTGTGGAGCACATGCCTTCAATCCAGAGG
GTAGTGTGTCGTAATGGGCAACTCTGCAGCGGAACCGACTACTTTGGGTGTCCGTGTTTCCTTTTATCTTTACATTGGCTGCTTATGGTGACGATTATAG
AATTGTTACCATATAGCTATTGGATTGGCCATCCGGTGTGCAATAGAGCTATTATATACCTGTTTGTTGGCTTTGTACCACTAACCTTAAAATCTATAAC
CACCCTCGATTTTATATTAACCCTCAATACAATCAAACATGGGCTCACAGGTGTCTACTCAGCGATCCGGCTCCCACGAGAACTCCAATTCGGCTACAGA
AGGCTCCACCATTAATTACACTACCATCAACTATTACAAAGACTCCTATGCTGCGACAGCGGGCAAACAGAGCCTCAAGCAAGACCCTGATAAATTTGCT
AACCCTGTCAAGGACATTTTCACTGAAATGGCTGCACCACTGAAGTCTCCATCCGCTGAGGCTTGTGGTTACAGTGATCGCGTGGCACAACTCACCATTG
GAAACTCCACCATCACTACACAGGAGGCGGCGAATATCATAGTCGGTTATGGTGAGTGGCCCTCATACTGCTCTGATGACGATGCTACAGCGGTGGACAA
GCCAACGCGCCCAGATGTTTCAGTGAATAGGTTTTATACGTTGGATACTAAATTGTGGGAAAAGTCATCCAAGGGGTGGTATTGGAAGTTTCCTGATGTA
CTGACTGAGACCGGAGTCTTTGGCCAGAATGCACAGTTTCACTATTTATATAGGTCAGGATTTTGCATTCATGTGCAATGTAATGCTAGCAAGTTCCATC
AAGGAGCGTTGTTAGTCGCCATACTTCCAGAGTATGTTATAGGGACAGTGGCAGGCGGCACAGGAACTGAGGACAGCCACCCTCCTTACAAACAAACACA
ACCTGGCGCCGATGGTTTTGAGTTGCAGCACCCGTACGTACTCGATGCTGGGATTCCTATATCACAATTAACAGTGTGCCCCCACCAATGGATTAACCTA
CGGACCAATAACTGTGCCACAATAATAGTGCCGTATATGAACACACTGCCTTTCGACTCTGCCCTGAACCATTGCAACTTTGGGCTGTTGGTGGTGCCCA
TTAGCCCACTAGATTTTGACCAAGGGGCAACTCCGGTTATCCCTATTACAATCACTCTAGCTCCAATGTGCTCTGAGTTTGCAGGTCTCAGACAGGCGGT
CACTCAAGGTTTTCCCACCGAGCCAAAACCAGGAACGAATCAATTTTTGACCACCGATGACGGTGTCTCAGCACCCATTTTACCAAATTTCCACCCCACA
CCATGTATTCACATACCCGGTGAAGTCAGAAACCTGCTTGAGTTGTGTCAAGTGGAGACCATTCTTGAGGTTAACAATGTACCCACCAATGCCACCAGTC
TGATGGAAAGGCTACGATTCCCGGTGTCCGCGCAAGCGGGAAAAGGTGAATTGTGTGCCGTGTTTAGGGCCGACCCTGGAAGAGACGGTCCATGGCAATC
AACAATGCTGGGCCAGTTGTGTGGATATTACACCCAGTGGTCAGGATCACTGGAGGTTACTTTTATGTTCACCGGGTCTTTCATGGCCACGGGTAAAATG
CTCATAGCTTATACACCTCCTGGTGGCCCCTTACCCAAAGATCGGGCCACAGCAATGCTGGGCACACATGTTATCTGGGATTTTGGGCTACAATCATCTG
TCACCCTTGTAATACCATGGATTAGCAACACCCACTACAGAGCGCATGCCCGGGATGGAGTGTTCGATTACTATACCACAGGACTGGTTAGTATCTGGTA
TCAAACAAACTACGTGGTTCCAATTGGGGCACCCAACACAGCTTACATAATAGCACTAGCGGCAGCCCAGAAGAATTTTACCATGAAACTGTGTAAAGAC
ACCAGTCACATATTACAGACAGCCTCTATTCAGGGAGATAGAGTGGCAGATGTGATAGAGAGCTCTATAGGAGATAGTGTGAGTAGGGCACTTACCCAGG
CCCTGCCAGCTCCAACAGGTCAGAACACGCAGGTGAGCAGTCATCGACTAGACACTGGTGAAGTTCCAGCGCTCCAAGCTGCTGAAATAGGGGCATCGTC
AAATACTAGTGATGAGAGTATGATTGAGACACGATGCGTTCTTAATTCACACAGTACGGCAGAGACCACCCTGGACAGCTTCTTCAGTAGGGCAGGCTTG
GTAGGAGAGATAGATCTCCCTACTGAGGGTACCACTAATCCAAATGGTTATGCTAATTGGGATATAGACATAACTGGTTACGCACAAATGCGCAGGAAAG
TGGAGCTGTTCACCTACATGCGCTTTGATGCGGAATTCACTTTTGTTGCGTGCACTCCTACTGGTCAGGTTGTCCCACAATTACTTCAGTATATGTTTGT
TCCCCCTGGTGCTCCCAAACCAGAGTCTAGAGAATCACTTGCTTGGCAGACAGCCACAAACCCCTCAGTTTTTGTCAAGTTGACTGATCCCCCGGCACAG
GTCTCAGTTCCGTTCATGTCACCCGCGAGCGCTTACCAGTGGTTTTACGACGGGTACCCCACGTTTGGAGAACACAAACAGGAGAAAGACCTTGAGTATG
GAGCGTGCCCTAATAATATGATGGGCACTTTCTCGGTGCGAACTGTGGGTTCATCAAAGTCCAAGTATTCTTTGGTTGTCAGGATATATATGAGAATGAA
GCATGTCAGGGCGTGGATACCTCGCCCGATGCGCAACCAAAACTACCTGTTTAAAGCCAATCCAAACTATGCCGGTAACTCCATCAAACCGACCGGCACT
AGTCGTGCTGCCATTACTACCCTTGGAAAGTTCGGCCAGCAATCTGGGGCCATCTACGTGGGCAACTTCAGAGTGGTTAATCGTCACCTCGCTACTCATA
ATGACTGGGCGAACCTCGTCTGGGAAGATAGCTCCCGCGACCTATTAGTGTCGTCTACCACCGCCCAGGGCTGTGATACAATTGCACGTTGTGACTGTCA
AACAGGAGTGTACTATTGTAATTCCAAAAGAAAGCACTATCCAGTCAGCTTCTCCAAACCCAGCCTCATATATGTGGAGGCTAGCGAGTATTACCCTGCT
AGATACCAATCGCACCTGATGCTTGCAGCAGGCCACTCTGAGCCCGGCGACTGCGGGGGCATCTTAAGGTGTCAACATGGTGTAGTTGGTATAGTGTCCA
CGGGTGGCAACGGGCTCGTTGGTTTTGCTGATGTGAGGGATCTCTTGTGGTTGGATGAAGAGGCCATGGAGCAAGGTGTGTCTGACTACATTAAGGGGCT
CGGTGACGCATTTGGAACAGGTTTCACTGATGCTGTATCCAGGGAAGTTGAAGCCCTCAGGAACCACCTCATAGGATCTGATGGAGCAGTGGAAAAAATC
CTAAAGAACCTTATTAAGCTGATTTCAGCGTTAGTAATTGTGATTAGGAGCGATTATGATATGGTCACCCTCACAGCAACTTTAGCCCTGATTGGTTGTC
ATGGAAGTCCCTGGGCTTGGATTAAAGCCAAAACAGCATCCATTTTAGGTATCCCCATCGCCCAGAAGCAGAGCGCTTCTTGGCTAAAGAAATTTAATGA
TATGGCGAGTGCTGCCAAGGGTTTAGAATGGATATCCAACAAAATTAGTAAGTTCATTGACTGGCTCAGGGAGAAGATTGTTCCAGCAGCTAAAGAGAAA
GCAGAATTTTTAACCAATTTGAAGCAATTACCACTATTAGAGAACCAGATCACGAACTTGGAGCAGTCCGCTGCCTCGCAAGAGGACCTTGAAGCTATGT
TTGGGAATGTGTCATACCTCGCCCATTTCTGTCGCAAGTTCCAACCATTATACGCCACGGAGGCCAAGCGAGTCTATGTTCTAGAGAAGAGAATGAACAA
TTACATGCAGTTCAAGAGCAAACACCGTATTGAACCTGTATGTCTCATCATTAGAGGCTCACCAGGCACTGGAAAGTCCCTTGCGACCGGCATCATTGCC
CGGGCCATAGCAGACAAGTACCACTCTAGTGTGTACTCACTCCCACCGGATCCTGACCATTTTGACGGGTACAAACAGCAAGTGGTTACAGTTATGGATG
ACCTGTGCCAGAATCCTGACGGCAAAGACATGTCATTATTTTGCCAGATGGTATCCACCGTGGATTTTATTCCACCAATGGCTTCTCTCGAAGAAAAGGG
AGTTTCTTTCACATCTAAATTTGTTATCGCATCCACCAACGCCAGCAACATTATAGTGCCCACAGTGTCTGACTCTGACGCCATTCGTCGCAGGTTCTAC
ATGGATTGCGACATTGAGGTCACAGACTCATACAAAACAGACTTGGGTAGACTAGACGCTGGGCGGGCTGCTAAGTTATGCTCTGAAAACAACACCGCAA
ATTTCAAACGATGCAGCCCACTAGTGTGTGGGAAAGCTATTCAACTTAGAGACAGGAAATCCAAGGTCAGGTATAGCGTGGACACAGTGGTCTCTGAACT
TATTAGAGAATACAATAGCAGATCCGCTATTGGTAACACAATTGAAGCATTATTCCAAGGCCCACCCAAGTTCAGGCCAATAAGGATCAGTCTTGAGGAG
AAGCCAGCCCCAGACGCTATTAGCGATCTCCTTGCTAGTGTGGATAGCGAGGAAGTGCGCCAATACTGTAGGGAACAAGGCTGGATTATCCCTGAAACTC
CCACCAATGTTGAACGACATCTTAATAGAGCAGTGCTAGTCGTGCAATCCATCACTACTGTGGTGGCAGTCGTCTCACTGGTGTACGTCATTTACAAGCT
CTTTGCGGGGTTTCAAGGTGCGTATTCTGGAGCTCCCAAGCAAGTGCTCAAGAAGCCTGTCCTCCGCACGGCAACAGTGCAGGGTCCAAGCCTTGATTTT
GCCCTATCCTTGCTGAGGAGGAACATCAGGCAAGTCCAAACAGACCAAGGGCATTTTACCATGTTGGGTGTCAGGGATCGCCTGGCTGTTCTCCCGCGGC
ACTCACAGCCCGGGAAGACTATTTGGGTGGAACACAAACTTGTGAACATCCTTGATGCAGTCGAGCTGGTGGACGAGCAGGGCGTTAATTTGGAACTCAC
ATTGGTGACACTAGATATTAATGAAAAATTTAGAGATATCACCAAGTTCATTCCAGAGACCATTAGCGGCGCTAGTGATGCAACTCTAGTGATCAACACA
GAACATATGCCGTCAATGTTTGTCCCTGTGGGGGACGTCGTGCAGTACGGGTTCTTGAACCTCAGTGGAAAGCCAACACATAGGACCATGATGTACAATT
TCCCTACAAAAGCAGGACAGTGTGGAGGCGTGGTTACATCAGTCGGTAAGATTGTTGGTATTCACATTGGTGGCAACGGGCGCCAAGGGTTCTGCGCTGG
TTTGAAGAGGAGCTACTTTGCGAGTATGCAGGGTGAGATCCAATGGGTGAAGCCTAACAAGGAAACTGGCAGACTAAACATCAATGGACCAACTCGCACT
AAGTTGGAGCCTAGTGTATTTCATGATGTGTTTGAAGGCAACAAGGAACCAGCAGTTTTAACAAGTAAAGACCCTAGATTGGAGGTCGACTTTGAACAAG
CCCTGTTTTCCAAGTATGTGGGCAATGTTTTACACGAGCCCGATGAATATGTGACTCAAGCTGCCCTCCACTATGCGAATCAACTTAAACAATTGGACAT
AAACACTAGCAAGATGAGCATGGAGGAAGCGTGCTATGGCACTGAAAACCTGGAAGCAATAGACCTCTGCACTAGTGCTGGGTATCCATACAGTGCCCTT
GGTATCAAGAAAAGAGACATTCTCGACCCCATAACCAGGGATGTGTCTAAGATGAAATTCTACATGGATAAATACGGACTAGATCTGCCATACTCTACCT
ATGTGAAGGATGAACTTAGATCTCTGGATAAAATCAAGAAAGGAAAGTCACGCCTGATAGAGGCCAGCAGCTTGAATGACTCTGTCTACCTCAGAATGAC
TTTTGGGCACCTTTACGAGGTGTTTCATGCTAACCCTGGTACTGTGACTGGCTCAGCAGTAGGTTGCAACCCAGACGTGTTTTGGAGTAAACTACCGATT
CTGCTGCCTGGGTCACTCTTTGCCTTTGACTACTCAGGATATGATGCTAGTCTCAGCCCGGTATGGTTCAGGGCTCTAGAAGTTGTGTTACGGGAGATTG
GGTATTCAGAGGAGGCCGTGTCCCTAATAGAAGGAATCAACCACACCCACCATGTGTACCGGAATAAAACATACTGTGTACTTGGTGGGATGCCCTCAGG
GTGCTCTGGTACTTCCATCTTCAATTCAATGATCAACAACATCATCATTAGAACCCTTTTGATCAAAACCTTTAAGGGAATAGACCTGGATGAGTTGAAC
ATGGTGGCCTATGGGGACGATGTGCTGGCCAGTTACCCTTTTCCTATTGATTGCCTTGAATTGGCTAAGACTGGCAAAGAGTATGGTTTGACCATGACTC
CTGCAGACAAATCACCCTGTTTCAATGAAGTAACATGGGAGAATGCTACCTTCCTGAAGARAGGGTTCTTGCCAGACCACCAATTTCCATTCTTAATTCA
CCCTACGATGCCCATGAGAGAGATCCATGAGTCCATTCGATGGACTAAGGACGCGCGTAACACCCAGGATCACGTGCGCTCCCTGTGTCTATTGGCATGG
CACAATGGTAAGGATGAATATGAAAAGTTTGTGAGTGCAATTAGATCAGTTCCAGTTGGAAAAGCGTTGGCCATTCCTAACTTTGAGAATCTGAGAAGAA
ATTGGCTCGAATTGTTTTAATA

## >EV-A71_small plaque isolate (SP3) DE NOVO ASSEMBLY

TGTGGGTTGTACCCACTCACAGGGCCCACGTGGCGCTAGCACTCTGGTTTTGCGGAACCTTTGTGCGCCTGTTTTATGCCCCCCCCCCCAATTTGCAACTT
AGAAGCAATACACAACACTGATCAACAGCAGGCATGGCGCACCAGCTATGTCTTGATCAAGCACTTCTGTTTCCCCGGGCCGAGTATCAATAGACTGTTC
ACGCGGTTGAAGGAGAAAGCGCCCGTTATCCGGCTAACTACTTCGAGAAACCTAGTAGCACCATTGAAGCTGCAGAGTGTTTCGCTCGGCACTTCCCCCG
TGTAGATCAGGTCGATGAGTCACTGCAATCCCCACGGGCGACCGTGGCAGTGGCTGCGCTGGCGGCCTGCCTATGGGGCAACCCATAGGACGCTCTAATG
TGGACATGGTGCGAAGAGTCTATTGAGCTAGTTAGTAGTCCTCCGGCCCCTGAATGCGGCTAATCCTAACTGTGGAGCACATGCCTTCAATCCAGAGGGT
AGTGTGTCGTAATGGGCAACTCTGCAGCGGAACCGACTACTTTGGGTGTCCGTGTTTCCTTTTATCTTTACATTGGCTGCTTATGGTGACGATTATAGAA
TTGTTACCATATAGCTATTGGATTGGCCATCCGGTGTGCAATAGAGCTATTATATACCTGTTTGTTGGCTTTGTACCACTAACCTTAAAATCTATAACCA
CCCTCGATTTTATATTAACCCTCAATACAATCAAACATGGGCTCACAGGTGTCTACTCAGCGATCCGGCTCCCACGAGAACTCCAATTCAGCTACAGAAG
GCTCCACCATTAATTACACTACCATCAACTATTACAAAGACTCCTATGCTGCGACAGCGGGCAAACAGAGCCTCAAGCAAGACCCTGATAAATTTGCTAA
CCCTGTCAAGGACATTTTCACTGAAATGGCTGCACCACTGAAGTCTCCATCCGCTGAGGCTTGTGGTTACAGTGATCGCGTGGCACAACTCACCATTGGA
AACTCCACCATCACTACACAGGAGGCGGCGAATATCATAGTCGGTTATGGTGAGTGGCCCTCATACTGCTCTGATGACGATGCTACAGCGGTGGACAAGC
CAACGCGCCCAGATGTTTCAGTGAATAGGTTTTATACGTTGGATACTAAATTGTGGGAAAAGTCATCCAAGGGGTGGTATTGGAAGTTTCCTGATGTACT
GACTGAGACCGGAGTCTTTGGCCAGAATGCACAGTTTCACTATTTATATAGGTCAGGATTTTGCATTCATGTGCAATGTAATGCTAGCAAGTTCCATCAA
GGAGCGTTGTTAGTCGCCATACTTCCAGAGTATGTTATAGGGACAGTGGCAGGCGGCACAGGAACTGAGGACAGCCACCCTCCCTACAAACAAACACAAC
CTGGCGCCGATGGTTTTGAGTTGCAGCACCCGTACGTACTCGATGCTGGGATTCCTATATCACAATTAACAGTGTGCCCCCACCAATGGATTAACCTACG
GACCAATAACTGTGCCACAATAATAGTGCCGTATATGAACACACTGCCTTTCGACTCTGCCCTGAACCATTGCAACTTTGGGCTGTTGGTGGTGCCCATT
AGCCCACTAGATTTTGACCAAGGGGCAACTCCGGTTATCCCTATTACAATCACTCTAGCTCCAATGTGCTCTGAGTTTGCAGGTCTCAGACAGGCGGTCA
CTCAAGGTTTTCCCACCGAGCCAAAACCAGGAACGAATCAATTTTTGACCACCGATGACGGTGTCTCAGCACCCATTTTACCAAATTTCCACCCCCACACC
ATGTATTCACATACCCGGTGAAGTCAGAAACCTGCTTGAGTTGTGTCAAGTGGAGACCATTCTTGAGGTTAACAATGTACCCACCAATGCCACCAGTCTG
ATGGAAAGGCTACGATTCCCGGTGTCCGCGCAAGCGGGAAAAGGTGAATTGTGTGCCGTGTTTAGGGCCGACCCTGGAAGAGACGGTCCATGGCAATCAA
CAATGCTGGGCCAGTTGTGTGGATATTACACCCAGTGGTCAGGATCACTGGAGGTTACTTTTATGTTCACCGGGTCTTTCATGGCCACGGGTAAAATGCT
CATAGCTTATACACCTCCTGGTGGCCCCTTACCCAAAGATCGGGCCACAGCAATGCTGGGCACACATGTTATCTGGGATTTTGGGCTACAATCATCTGTC
ACCCTTGTAATACCATGGATTAGCAACACCCACTACAGAGCGCATGCCCGGGATGGAGTGTTCGATTACTATACCACAGGACTGGTTAGTATCTGGTATC
AAACAAACTACGTGGTTCCAATTGGGGCACCCAACACAGCTTACATAATAGCACTAGCGGCAGCCCAGAAGAATTTTACCATGAAACTGTGTAAAGACAC
CAGTCACATATTACAGACAGCCTCTATTCAGGGAGATAGAGTGGCAGATGTGATAGAGAGCTCTATAGGAGATAGTGTGAGTAGGGCACTTACCCAGGCC
CTGCCAGCTCCAACAGGTCAGAACACGCAGGTGAGCAGTCATCGACTAGACACTGGTGAAGTTCCAGCGCTCCAAGCTGCTGAAATAGGGGCATCGTCAA
ATACTAGTGATGAGAGTATGATTGAGACACGATGCGTTCTTAATTCACACAGTACGGCAGAGACCACCCTGGACAGCTTCTTCAGTAGGGCAGGCTTGGT
AGGAGAGATAGATCTCCCTACTGAGGGTACCACTAATCCAAATGGTTATGCTAATTGGGATATAGACATAACTGGTTACGCACAAATGCGCAGGAAAGTG
GAGCTGTTCACCTACATGCGCTTTGATGCGGAATTCACTTTTGTTGCGTGCACTCCTACTGGTCAGGTTGTCCCACAATTACTTCAGTATATGTTTGTTC
CCCCTGGTGCTCCCAAACCAGAGTCTAGAGAATCACTTGCTTGGCAGACAGCCACAAACCCCTCAGTTTTTGTCAAGTTGACTGATCCCCCGGCACAGGT
CTCAGTTCCGTTCATGTCACCCGCGAGCGCTTACCAGTGGTTTTACGACGGGTACCCCACGTTTGGAGAACACAAACAGGAGAAAGACCTTGAGTATGGA
GCGTGCCCTAATAATATGATGGGCACTTTCTCGGTGCGAACTGTGGGTTCATCAAAGTCCAAGTATTCTTTGGTTGTCAGGATATATATGAGAATGAAGC
ATGTCAGGGCGTGGATACCTCGCCCGATGCGCAACCAAAACTACCTGTTTAAAGCCAATCCAAACTATGCCGGTAACTCCATCAAACCGACCGGCACTAG
TCGTACTGCCATTACTACCCTTGGAAAGTTCGGCCAGCAATCTGGGGCCATCTACGTGGGCAACTTCAGAGTGGTTAATCGTCACCTCGCTACTCATAAT
GACTGGGCGAACCTCGTCTGGGAAGATAGCTCCCGCGACCTATTAGTGTCGTCTACCACCGCCCAGGGCTGTGATACAATTGCACGTTGTGACTGTCAAA
CAGGAGTGTACTATTGTAATTCCAAAAGAAAGCACTATCCAGTCAGCTTCTCCAAACCCAGCCTCATATATGTGGAGGCTAGCGAGTATTACCCTGCTAG
ATACCAATCGCACCTGATGCTTGCAGCAGGCCACTCTGAGCCCGGCGACTGCGGGGGCATCTTAAGGTGTCAACATGGTGTAGTTGGTATAGTGTCCACG
GGTGGCAACGGGCTCGTTGGTTTTGCTGATGTGAGGGATCTCTTGTGGTTGGATGAAGAGGCCATGGAGCAAGGTGTGTCTGACTACATTAAGGGGCTCG
GTGACGCATTTGGAACAGGTTTCACTGATGCTGTATCCAGGGAAGTTGAAGCCCTCAGGAACCACCTCATAGGATCTGATGGAGCAGTGGAAAAAATCCT
AAAGAACCTTATTAAGCTGATTTCAGCGTTAGTAATTGTGATTAGGAGCGATTATGATATGGTCACCCTCACAGCAACTTTAGCCCTGATTGGTTGTCAT
GGAAGTCCCTGGGCTTGGATTAAAGCCAAAACAGCATCCATTTTAGGTATCCCCATCGCCCAGAAGCAGAGCGCTTCTTGGCTAAAGAAATTTAATGATA
TGGCGAGTGCTGCCAAGGGTTTAGAATGGATATCCAACAAAATTAGTAAGTTCATTGACTGGCTCAGGGAGAAGATTGTTCCAGCAGCTAAAGAGAAAGC
AGAATTTTTAACCAATTTGAAGCAATTACCACTATTAGAGAACCAGATCACGAACTTGGAGCAGTCCGCTGCCTCGCAAGAGGACCTTGAAGCTATGTTT
GGGAATGTGTCATACCTCGCCCATTTCTGTCGCAAGTTCCAACCATTATACGCCACGGAGGCCAAGCGAGTCTATGTTCTAGAGAAGAGAATGAACAATT
ACATGCAGTTCAAGAGCAAACACCGTATTGAACCTGTATGTCTCATCATTAGAGGCTCACCAGGCACTGGAAAGTCCCTTGCGACCGGCATCATTGCCCG
GGCCATAGCAGACAAGTACCACTCTAGTGTGTACTCACTCCCACCGGATCCTGACCATTTTGACGGGTACAAACAGCAAGTGGTTACAGTTATGGATGAC
CTGTGCCAGAATCCTGACGGCAAAGACATGTCATTATTTTGCCAGATGGTATCCACCGTGGATTTTATTCCACCAATGGCTTCTCTCGAAGAAAAGGGAG
TTTCTTTCACATCTAAATTTGTTATCGCATCCACCAACGCCAGCAACATTATAGTGCCCACAGTGTCTGACTCTGACGCCATTCGTCGCAGGTTCTACAT
GGATTGCGACATTGAGGTCACAGACTCATACAAAACAGACTTGGGTAGACTAGACGCTGGGCGGGCTGCTAAGTTATGCTCTGAAAACAACACCGCAAAT
TTCAAACGATGCAGCCCACTAGTGTGTGGGAAAGCTATTCAACTTAGAGACAGGAAATCCAAGGTCAGGTATAGCGTGGACACAGTGGTCTCTGAACTTA
TTAGAGAATACAATAGCAGATCCGCTATTGGTAACACAATTGAAGCATTATTCCAAGGCCCACCCAAGTTCAGGCCAATAAGGATCAGTCTTGAGGAGAA
GCCAGCCCCAGACGCTATTAGCGATCTCCTTGCTAGTGTGGATAGCGAGGAAGTGCGCCAATACTGTAGGGAACAAGGCTGGATTATCCCTGAAACTCCC
ACCAATGTTGAACGACATCTTAATAGAGCAGTGCTAGTCGTGCAATCCATCACTACTGTGGTGGCAGTCGTCTCACTGGTGTACGTCATTTACAAGCTCT
TTGCGGGGTTTCAAGGTGCGTATTCTGGAGCTCCCAAGCAAGTGCTCAAGAAGCCTGTCCTCCGCACGGCAACAGTGCAGGGTCCAAGCCTTGATTTGC
CCTATCCTTGCTGAGGAGGAACATCAGGCAAGTCCAAACAGACCAAGGGCATTTTACCATGTTGGGTGTCAGGGATCGCCTGGCTGTTCTCCCGCGGCAC
TCACAGCCCGGGAAGACTATTTGGGTGGAACACAAACTTGTGAACATCCTTGATGCAGTCGAGCTGGTGGACGAGCAGGGCGTTAATTTGGAACTCACAT
TGGTGACACTAGATATTAATGAAAAATTTAGAGATATCACCAAGTTCATTCCAGAGACCATTAGCGGCGCTAGTGATGCAACTCTAGTGATCAACACAGA
ACATATGCCGTCAATGTTTGTCCCTGTGGGGGACGTCGTGCAGTACGGGTTCTTGAACCTCAGTGGAAAGCCAACACATAGGACCATGATGTACAATTTC
CCTACAAAAGCAGGACAGTGTGGAGGCGTGGTTACATCAGTCGGTAAGATTGTTGGTATTCACATTGGTGGCAACGGGCGCCAAGGGTTCTGCGCTGGTT
TGAAGAGGAGCTACTTTGCGAGTATGCAAGGTGAGATCCAATGGGTGAAGCCTAACAAGGAAACTGGCAGACTAAACATCAATGGACCAACTCGCACTAA
GTTGGAGCCTAGCGTATTTCATGATGTGTTTGAAGGCAACAAGGAACCAGCAGTTTTAACAAGTAAAGACCCTAGATTGGAGGTCGACTTTGAACAAGCC
CTGTTTTCCAAGTATGTGGGCAATGTTTTACACGAGCCCGATGAATATGTGACTCAAGCTGCCCTCCACTATGCGAATCAACTTAAACAATTGGACATAA
ACACTAGCAAGATGAGCATGGAGGAAGCGTGCTATGGCACTGAAAACCTGGAAGCAATAGACCTCTGCACTAGTGCTGGGTATCCATACAGTGCCCTTGG
TATCAAGAAAAGAGACATTCTCGACCCCATAACCAGGGATGTGTCTAAGATGAAATTCTACATGGATAAATACGGACTAGATCTGCCATACTCTACCTAT
GTGAAGGATGAACTTAGATCTCTGGATAAAATCAAGAAAGGAAAGTCACGCCTGATAGAGGCCAGCAGCTTGAATGACTCTGTCTACCTCAGAATGACTT
TTGGGCACCTTTACGAGGTGTTTCATGCTAACCCTGGTACTGTGACTGGCTCAGCAGTAGGTTGCAACCCAGACGTGTTTTGGAGTAAACTACCGATTCT
GCTGCCTGGGTCACTCTTTGCCTTTGACTACTCAGGATATGATGCTAGTCTCAGCCCGGTATGGTTCAGGGCTCTAGAAGTTGTGTTACGGGAGATTGGG
TATTCAGAGGAGGCCGTGTCCCTAATAGAAGGAATCAACCACACCCACCATGTGTACCGGAATAAAACATACTGTGTACTTGGTGGGATGCCCTCAGGGT
GCTCTGGTACTTCCATCTTCAATTCAATGATCAACAACATCATCATTAGAACCCTTTTGATCAAAACCTTTAAGGGAATAGACCTGGATGAGTTGAACAT
GGTGGCCTATGGGGACGATGTGCTGGCCAGTTACCCTTTTCCTATTGATTGTCTTGAATTGGCTAAGACTGGCAAAGAGTATGGTTTGACCATGACTCCT
GCAGACAAATCACCCTGTTTCAATGAAGTAACATGGGAGAATGCTACCTTCCTGAAGAGAGGGTTCTTGCCAGACCACCAATTTCCATTCTTAATTCACC
CTACGATGCCCATGAGAGAGATCCATGAGTCCATTCGATGGACTAAGGACGCGCGTAACACCCAGGATCACGTGCGCTCCCTGTGTCTATTGGCATGGCA
CAATGGTAAGGATGAATATGAAAAGTTTGTGAGTGCAATTAGATCAGTTCCAGTTGGAAAAGCGTTGGCCATTCCTAACTTTGAGAATCTGAGAAGAAAT
TGGCTNGAATTGTTTTAATATTACAGTTTAAAGNTGAACCCCACTAGAAAT

# >EV-A71_small plaque isolate (SP4) DE NOVO ASSEMBLY

---TTTTAAAACA--
AGCTGTGGGTTGTACCCACTCACAGGGCCCACGTGGCGCTAGCACTCTGGTTCTGCGGAACCTTTGTGCGCCTGTTTTACGCCCCCCCCCCAATTTGCAA
CTTAGAAGCAATACACAACACTGATCAACAGCAGGCATGGCGCACCAGCTATGTCTTGATCAAGCACTTCTGTTTCCCCGGGCCGAGTATCAATAGACTG
TTCACGCGGTTGAAGGAGAAAGCGCCCGTTATCCGGCTAACTACTTCGAGAAACCTAGTAGCACCATTGAAGCTGCAGAGTGTTTCGCTCGGCACTTCCC
CCGTGTAGATCAGGTCGATGAGTCACTGCAATCCCCACGGGCGACCGTGGCAGTGGCTGCGCTGGCGGCCTGCCTATGGGGCAACCCATAGGACGCTCTA
ATGTGGACATGGTGCGAAGAGTCTATTGAGCTAGTTAGTAGTCCTCCGGCCCCTGAATGCGGCTAATCCTAACTGTGGAGCACATGCCTTCAATCCAGAG
GGTAGTGTGTCGTAATGGGCAACTCTGCAGCGGAACCGACTACTTTGGGTGTCCGTGTTTCCTTTTATCTTTACATTGGCTGCTTATGGTGACGATTATA
GAATTGTTACCATATAGCTATTGGATTGGCCATCCGGTGTGCAATAGAGCTATTATATACCTGTTTGTTGGCTTTGTACCACTAACCTTAAAATCTATAA
CCACCCTCGATTTTATATTAACCCTCAATACAATCAAACATGGGCTCACAGGTGTCTACTCAGCGATCCGGCTCCCACGAGAACTCCAATTCGGCTACAG
AAGGCTCCACCATTAATTACACTACCATCAACTATTACAAAGACTCCTATGCTGCGACAGCGGGCAAACAGAGCCTCAAGCAAGACCCTGATAAATTTGC
TAACCCTGTCAAGGACATTTTCACTGAAATGGCTGCACCACTGAAGTCTCCATCCGCCGAGGCTTGTGGTTACAGTGATCGCGTGGCACAACTCACCATT
GGAAACTCCACCATCACTACACAGGAGGCGGCGAATATCATAGTCGGTTATGGTGAGTGGCCCTCATACTGCTCTGATGACGATGCTACAGCGGTGGACA
AGCCAACGCGCCCAGATGTTTCAGTGAATAGGTTTTATACGTTGGATACTAAATTGTGGGAAAAGTCATCCAAGGGGTGGTATTGGAAGTTTCCTGATGT
ACTGACTGAGACCGGAGTCTTTGGCCAGAATGCACAGTTTCACTATTTATATAGGTCAGGATTTTGCATTCATGTGCAATGTAATGCTAGCAAGTTCCAT
CAAGGAGCGTTGTTAGTCGCCATACTTCCAGAGTATGTTATAGGGACAGTGGCAGGCGGCACAGGAACTGAGGACAGCCACCCTCCTTACAAACAAACAC
AACCTGGCGCCGATGGTTTTGAGTTGCAGCACCCGTACGTACTCGATGCTGGGATTCCTATATCACAATTAACAGTGTGCCCCCATCAATGGATTAACCT
ACGGACCAATAACTGTGCCACAATAATAGTGCCGTATATGAACACACTGCCTTTCGACTCTGCCCTGAACCATTGCAACTTTGGGCTGTTGGTGGTGCCC
ATTAGCCCACTAGATTTTGACCAAGGGGCAACTCCGGTTATCCCTATTACAATCACTCTAGCTCCAATGTGCTCTGAGTTTGCAGGTCTCAGACAGGCGG
TCACTCAAGGTTTTCCCACCGAGCCAAAACCAGGAACGAATCAATTTTTGACCACCGATGACGGTGTCTCAGCACCCATTTTACCAAATTTCCACCCCAC
ACCATGTATTCACATACCCGGTGAAGTCAGAAACCTGCTTGAGTTGTGTCAAGTGGAGACCATTCTTGAGGTTAACAATGTACCCACCAATGCCACCAGT
CTGATGGAAAGGCTACGATTCCCGGTGTCCGCGCAAGCGGGAAAAGGTGAATTGTGTGCCGTGTTTAGGGCCGACCCTGGAAGAGACGGTCCATGGCAAT
CAACAATGCTGGGCCAGTTGTGTGGATATTACACCCAGTGGTCAGGATCACTGGAGGTTACTTTTATGTTCACCGGGTCTTTCATGGCCACGGGTAAAAT
GCTCATAGCTTATACACCTCCTGGTGGCCCCTTACCCAAAGATCGGGCCACAGCAATGCTGGGCACACATGTTATCTGGGATTTTGGGCTACAATCATCT
GTCACCCTTGTAATACCATGGATTAGCAACACCCACTACAGAGCGCATGCCCGGGATGGAGTGTTCGATTACTATACCACAGGACTGGTTAGTATCTGGT
ATCAAACAAACTACGTGGTTCCAATTGGGGCACCCAACACAGCTTACATAATAGCACTAGCGGCAGCCCAGAAGAATTTTACCATGAAACTGTGTAAAGA
CACCAGTCACATATTACAGACAGCCTCTATTCAGGGAGATAGAGTGGCAGATGTGATAGAGAGCTCTATAGGAGATAGTGTGAGTAGGGCACTTACCCAG
GCCCTGCCAGCTCCAACAGGTCAGAACACGCAGGTGAGCAGTCATCGACTAGACACTGGTGAAGTTCCAGCGCTCCAAGCTGCTGAAATAGGGGCATCGT
CAAATACTAGTGATGAGAGTATGATTGAGACACGATGCGTTCTTAATTCACACAGTACGGCAGAGACCACCCTGGACAGCTTCTTCAGTAGGGCAGGCTT
GGTAGGAGAGATAGATCTCCCTACTGAGGGTACCACTAATCCAAATGGTTATGCTAATTGGGATATAGACATAACTGGTTACGCACAAATGCGCAGGAAA
GTGGAGCTGTTCACCTACATGCGCTTTGATGCGGAATTCACTTTTGTTGCGTGCACTCCTACTGGTCAGGTTGTCCCACAATTACTTCAGTATATGTTTG
TTCCCCCTGGTGCTCCCAAACCAGAGTCTAGAGAATCACTTGCTTGGCAGACAGCCACAAACCCCTCAGTTTTTGTCAAGTTGACTGATCCCCCGGCACA
GGTCTCAGTTCCGTTCATGTCACCCGCGAGCGCTTACCAGTGGTTTTACGACGGGTACCCCACGTTTGGAGAACACAAACAGGAGAAAGACCTTGAGTAT
GGAGCGTGCCCTAATAATATGATGGGCACTTTCTCGGTGCGAACTGTGGGTTCATCAAAGTCCAAGTATTCTTTGGTTGTCAGGATATATATGAGAATGA
AGCATGTCAGGGCGTGGATACCTCGCCCGATGCGCAACCAAAACTACCTGTTTAAAGCCAATCCAAACTATGCCGGTAACTCCATCAAACCGACCGGCAC
TAGTCGTGCTGCCATTACTACCCTTGGAAAGTTCGGCCAGCAATCTGGGGCCATCTACGTGGGCAACTTCAGAGTGGTTAATCGTCACCTCGCTACTCAT
AATGACTGGGCGAACCTCGTCTGGGAAGATAGCTCCCGCGACCTATTAGTGTCGTCTACCACCGCCCAGGGCTGTGATACAATTGCACGTTGTGACTGTC
AAACAGGAGTGTACTATTGTAATTCCAAAAGAAAGCACTATCCAGTCAGCTTCTCCAAACCCAGCCTCATATATGTGGAGGCTAGCGAGTATTACCCTGC
TAGATACCAATCGCACCTGATGCTTGCAGCAGGCCACTCTGAGCCCGGCGACTGCGGGGGCATCTTAAGGTGTCAACATGGTGTAGTTGGTATAGTGTCC
ACGGGTGGCAACGGGCTCGTTGGTTTTGCTGATGTGAGGGATCTCTTGTGGTTGGATGAAGAGGCCATGGAGCAAGGTGTGTCTGACTACATTAAGGGGC
TCGGTGACGCATTTGGAACAGGTTTCACTGATGCTGTATCCAGGGAAGTTGAAGCCCTCAGGAACCACCTCATAGGATCTGATGGAGCAGTGGAAAAAAT
CCTAAAGAACCTTATTAAGCTGATTTCAGCGTTAGTAATTGTGATTAGGAGCGATTATGATATGGTCACCCTCACAGCAACTTTAGCCCTGATTGGTTGT
CATGGAAGTCCCTGGGCTTGGATTAAAGCCAAAACAGCATCCATTTTAGGTATCCCCATCGCCCAGAAGCAGAGCGCTTCTTGGCTAAAGAAATTTAATG
ATATGGCGAGTGCTGCCAAGGGTTTAGAATGGATATCCAACAAAATTAGTAAGTTCATTGACTGGCTCAGGGAGAAGATTGTTCCAGCAGCTAAAGAGAA
AGCAGAATTTTTAACCAATTTGAAGCAATTACCACTATTAGAGAACCAGATCACGAACTTGGAGCAGTCCGCTGCCTCGCAAGAGGACCTTGAAGCTATG
TTTGGGAATGTGTCATACCTCGCCCATTTCTGTCGCAAGTTCCAACCATTATACGCCACGGAGGCCAAGCGAGTCTATGTTCTAGAGAAGAGAATGAACA
ATTACATGCAGTTCAAGAGCAAACACCGTATTGAACCTGTATGTCTCATCATTAGAGGCTCACCAGGCACTGGAAAGTCCCTTGCGACCGGCATCATTGC
CCGGGCCATAGCAGACAAGTACCACTCTAGTGTGTACTCACTCCCACCGGATCCTGACCATTTTGACGGGTACAAACAGCAAGTGGTTACAGTTATGGAT
GACCTGTGCCAGAATCCTGACGGCAAAGACATGTCATTATTTTGCCAGATGGTATCCACCGTGGATTTTATTCCACCAATGGCTTCTCTCGAAGAAAAGG
GAGTTTCTTTCACATCTAAATTTGTTATCGCATCCACCAACGCCAGCAACATTATAGTGCCCACAGTGTCTGACTCTGACGCCATTCGTCGCAGGTTCTA
CATGGATTGCGACATTGAGGTCACAGACTCATACAAAACAGACTTGGGTAGACTAGACGCTGGGCGGGCTGCTAAGTTATGCTCTGAAAACAACACCGCA
AATTTCAAACGATGCAGCCCACTAGTGTGTGGGAAAGCTATTCAACTTAGAGACAGGAAATCCAAGGTCAGGTATAGCGTGGACACAGTGGTCTCTGAAC
TTATTAGAGAATACAATAGCAGATCCGCTATTGGTAACACAATTGAAGCATTATTCCAAGGCCCACCCAAGTTCAGGCCAATAAGGATCAGTCTTGAGGA
GAAGCCAGCCCCAGACGCTATTAGCGATCTCCTTGCTAGTGTGGATAGCGAGGAAGTGCGCCAATACTGTAGGGAACAAGGCTGGATTATCCCTGAAACT
CCCACCAATGTTGAACGACATCTTAATAGAGCAGTGCTAGTCGTGCAATCCATCACTACTGTGGTGGCAGTCGTCTCACTGGTGTACGTCATTTACAAGC
TCTTTGCGGGGTTTCAAGGTGCGTATTCTGGAGCTCCCAAGCAAGTGCTCAAGAAGCCTGTCCTCCGCACGGCAACAGTGCAGGGTCCAAGCCTTGATTT
TGCCCTATCCTTGCTGAGGAGGAACATCAGGCAAGTCCAAACAGACCAAGGGCATTTTACCATGTTGGGTGTCAGGGATCGCCTGGCTGTTCTCCCGCGG
CACTCACAGCCCGGGAAGACTATTTGGGTGGAACACAAACTTGTGAACATCCTTGATGCAGTCGAGCTGGTGGACGAGCAGGGCGTTAATTTGGAACTCA
CATTGGTGACACTAGATATTAATGAAAAATTTAGAGATATCACCAAGTTCATTCCAGAGACCATTAGCGGCGCTAGTGATGCAACTCTAGTGATCAACAC
AGAACATATGCCGTCAATGTTTGTCCCTGTGGGGGACGTCGTGCAGTACGGGTTCTTGAACCTCAGTGGAAAGCCAACACATAGGACCATGATGTACAAT
TTCCCTACAAAAGCAGGACAGTGTGGAGGCGTGGTTACATCAGTCGGTAAGATTGTTGGTATTCACATTGGTGGCAACGGGCGCCAAGGGTTCTGCGCTG
GTTTGAAGAGGAGCTACTTTGCGAGTATGCAAGGTGAGATCCAATGGGTGAAGCCTAACAAGGAAACTGGCAGACTAAACATCAATGGACCAACTCGCAC
TAAGTTGGAGCCTAGTGTATTTCATGATGTGTTTGAAGGCAACAAGGAACCAGCAGTTTTAACAAGTAAAGACCCTAGATTGGAGGTCGACTTTGAACAA
GCCCTGTTTTCCAAGTATGTGGGCAATGTTTTACACGAGCCCGATGAATATGTGACTCAAGCTGCCCTCCACTATGCGAATCAACTTAAACAATTGGACA
TAAACACTAGCAAGATGAGCATGGAGGAAGCGTGCTATGGCACTGAAAACCTGGAAGCAATAGACCTCTGCACTAGTGCTGGGTATCCATACAGTGCCCT
TGGTATCAAGAAAAGAGACATTCTCGACCCCATAACCAGGGATGTGTCTAAGATGAAATTCTACATGGATAAATACGGACTAGATCTGCCATACTCTACC
TATGTGAAGGATGAACTTAGATCTCTGGATAAAATCAAGAAAGGAAAGTCACGCCTGATAGAGGCCAGCAGCTTGAATGACTCTGTCTACCTCAGAATGA
CTTTTGGGCACCTTTACGAGGTGTTTCATGCTAACCCTGGTACTGTGACTGGCTCAGCAGTAGGTTGCAACCCAGACGTGTTTTGGAGTAAACTACCGAT
TCTGCTGCCTGGGTCACTCTTTGCCTTTGACTACTCAGGATATGATGCTAGTCTCAGCCCGGTATGGTTCAGGGCTCTAGAAGTTGTGTTACGGGAGATT
GGGTATTCAGAGGAGGCCGTGTCCCTAATAGAAGGAATCAACCACACCCACCATGTGTACCGGAATAAAACATACTGTGTACTTGGTGGGATGCCCTCAG
GGTGCTCTGGTACTTCCATCTTCAATTCAATGATCAACAACATCATCATTAGAACCCTTTTGATCAAAACCTTTAAGGGAATAGACCTGGATGAGTTGAA
CATGGTGGCCTATGGGGACGATGTGCTGGCCAGTTACCCTTTTCCTATTGATTGCCTTGAATTGGCTAAGACTGGCAAAGAGTATGGTTTGACCATGACT
CCTGCAGACAAATCACCCTGTTTCAATGAAGTAACATGGGAGAATGCTACCTTCCTGAAGAGAGGGTTCTTGCCAGACCACCAATTTCCATTCTTAATTC
ACCCTACGATGCCCATGAGAGAGATCCATGAGTCCATTCGATGGACTAAGGACGCGCGTAACACCCAGGATCACGTGCGCTCCCTGTGTCTATTGGCATG
GCACAATGGTAAGGATGAATATGAAAAGTTTGTGAGTGCAATTAGATCAGTTCCAGTTGGAAAAGCGTTGGCCATTCCTAACTTTGAGAATCTGAGAAGA
AATTGGCTCGAATTGTTTTAATATTACAGTTTAAAGCTGAACCCCACTAGAAATCTGGT

## >EV-A71_big plaque isolate (BP1) DE NOVO ASSEMBLY

ATTTTTAAAACAAAGGCTGTGGGTTGTACCCACTCACAGGGCCCACGTGGCGCTAGCACTCTGGTTCTGCGGAACCTTTGTGCGCCTGTTTTACGCCCC
CCCCCCAATTTGCAACTTAGAAGCAATACACAACACTGATCAACAGCAGGCATGGCGCACCAGCTATGTCTTGATCAAGCACTTCTGTTTCCCCGGGCCG
AGTATCAATAGACTGTTCACGCGGTTGAAGGAGAAAGCGCCCGTTATCCGGCTAACTACTTCGAGAAACCTAGTAGCACCATTGAAGCTGCAGAGTGCTT
CGCTCGGCACTTCCCCCGTGTAGATCAGGTCGATGAGTCACTGCAATCCCCACGGGCGACCGTGGCAGTGGCTGCGCTGGCGGCCTGCCTATGGGGCAAC
CCATAGGACGCTCTAATGTGGACATGGTGCGAAGAGTCTATTGAGCTAGTTAGTAGTCCTCCGGCCCCTGAATGCGGCTAATCCTAACTGTGGAGCACAT
GCCTTCAATCCAGAGGGTAGTGTGTCGTAATGGGCAACTCTGCAGCGGAACCGACTACTTTGGGTGTCCGTGTTTCCTTTTATCTTTACATTGGCTGCTT
ATGGTGACGATTATAGAATTGTTACCATATAGCTATTGGATTGGCCATCCGGTGTGCAATAGAGCTATTATATACCTGTTTGTTGGCTTTGTACCACTAA
CCTTAAAATCTATAACCACCCTCGATTTTATATTAACCCTCAATACAATCAAACATGGGCTCACAGGTGTCTACTCAGCGATCCGGCTCCCACGAGAACT
CCAATTCAGCTACAGAAGGCTCCACCATTAATTACACTACCATCAACTATTACAAAGACTCCTATGCTGCGACAGCGGGCAAACAGAGCCTCAAGCAAGA
CCCTGATAAATTTGCTAACCCTGTCAAGGACATTTTCACAGAAATGGCTGCACCACTGAAGTCTCCATCCGCTGAGGCTTGTGGTTACAGTGATCGCGTG
GCACAACTCACCATTGGAAACTCCACCATCACTACACAGGAGGCGGCGAATATCATAGTCGGTTATGGTGAGTGGCCCTCATACTGCTCTGATGACGATG
CTACAGCGGTGGACAAGCCAACGCGCCCAGATGTTTCAGTGAATAGGTTTTATACGTTGGATACTAAATTGTGGGAAAAGTCATCCAAGGGGTGGTATTG
GAAGTTTCCTGATGTACTGACTGAGACCGGAGTCTTTGGCCAGAATGCACAGTTTCACTATTTATATAGGTCAGGATTTTGCATTCATGTGCAATGTAAT
GCTAGCAAGTTCCATCAAGGAGCGTTGTTAGTCGCCATACTTCCAGAGTATGTTATAGGGACAGTGGCAGGCGGCACAGGAACTGAGGACAGCCACCCTC
CTTACAAACAAACACAACCTGGCGCCGATGGTTTTGAGTTGCAGCACCCGTACGTACTCGATGCTGGGATTCCTATATCACAATTAACAGTGTGCCCCCA
CCAATGGATTAACCTACGGACCAATAACTGTGCCACAATAATAGTGCCGTATATGAACACACTGCCTTTCGACTCTGCCCTGAACCATTGCAACTTTGGG
CTGTTGGTGGTGCCCATTAGCCCACTAGATTTTGACCAAGGGGCAACTCCGGTTATCCCTATTACAATCACTCTAGCTCCAATGTGCTCTGAGTTTGCAG
GTCTCAGACAGGCGGTCACTCAAGGTTTTCCCACCGAGCCAAAACCAGGAACGAATCAATTTTTGACCACCGATGACGGTGTCTCAGCACCCATTTTACC
AAATTTCCACCCCACACCATGTATTCACATACCCGGTGAAGTCAGAAACCTGCTTGAGTTGTGTCAAGTGGAGACCATTCTTGAGGTTAACAATGTACCC
ACCAATGCCACCAGTCTGATGGAAAGGCTACGATTCCCGGTGTCCGCGCAAGCGGGAAAAGGTGAATTGTGTGCCGTGTTTAGGGCCGACCCTGGAAGAG
ACGGTCCATGGCAATCAACAATGCTGGGCCAGTTGTGTGGATATTACACCCAGTGGTCAGGATCACTGGAGGTTACTTTTATGTTCACCGGGTCTTTCAT
GGCCACGGGTAAAATGCTCATAGCTTATACACCTCCTGGTGGCCCCTTACCCAAAGATCGGGCCACAGCAATGCTGGGCACACATGTTATCTGGGATTTT
GGGCTACAATCATCTGTCACCCTTGTAATACCATGGATTAGCAACACCCACTACAGAGCGCATGCCCGGGATGGAGTGTTCGATTACTATACCACAGGAC
TGGTTAGTATCTGGTATCAAACAAACTACGTGGTTCCAATTGGGGCACCCAACACAGCTTACATAATAGCACTAGCGGCAGCCCAGAAGAATTTTACCAT
GAAACTGTGTAAAGACACCAGTCACATATTACAGACAGCCTCTATTCAGGGAGATAGAGTGGCAGATGTGATAGAGAGCTCTATAGGAGATAGTGTGAGT
AGGGCACTTACCCAGGCCCTGCCAGCTCCAACAGGTCAGAACACGCAGGTGAGCAGTCATCGACTAGACACTGGTGAAGTTCCAGCGCTCCAAGCTGCTG
AAATAGGGGCATCGTCAAATACTAGTGATGAGAGTATGATTGAGACACGATGCGTTCTTAATTCACACAGTACGGCAGAGACCACCCTGGACAGCTTCTT
CAGTAGGGCAGGCTTGGTAGGAGAGATAGATCTCCCTCTTGAGGGTACCACTAATCCAAGTGGTTATGCTAATTGGGATATAGACATAACTGGTTACGCA
CAAATGCGCAGGAAAGTGGAGCTGTTCACCTACATGCGCTTTGATGCGGAATTCACTTTTGTTGCGTGCACTCCTACTGGTCAGGTTGTCCCACAATTAC
TTCAGTATATGTTTGTTCCCCCTGGTGCTCCCAAACCAGAGTCTAGAGAATCACTTGCTTGGCAGACAGCCACAAACCCCTCAGTTTTTGTCAAGTTGAC
TGATCCCCCGGCACAGGTCTCAGTTCCGTTCATGTCACCCGCGAGCGCTTACCAGTGGTTTTACGACGGGTACCCCACGTTTGGAGAACACAAACAGGAG
AAAGACCTTGAGTATGGAGCGTGCCCTAATAATATGATGGGCACTTTCTCGGTGCGAAATGTGGGTTCATCAAAGTCCAAGTATCCTTTGGTTGTCAGGA
TATATATGAGAATGAAGCATGTCAGGGCGTGGATACCTCGCCCGATGCGCAACCAAAACTACCTGTTTAAAGCCAATCCAAACTATGCCGGTGACTCCAT
CAAACCGACCGGCACTAGTCGTACTGCCATTACTACCCTTGGAAAGTTCGGCCAGCAATCTGGGGCCATCTACGTGGGCAACTTCAGAGTGGTTAATCGT
CACCTCGCTACTCATAATGACTGGGCGAACCTCGTCTGGGAAGATAGCTCCCGCGACCTATTAGTGTCGTCTACCACCGCCCAGGGCTGTGATACAATTG
CACGTTGTGACTGTCAAACAGGAGTGTACTATTGTAATTCCAAAAGAAAGCACTATCCAGTCAGCTTCTCCAAACCCAGCCTCATATATGTGGAGGCTAG
CGAGTATTACCCTGCTAGATACCAATCGCACCTGATGCTTGCAGCAGGCCACTCTGAGCCCGGCGACTGCGGGGGCATCTTAAGGTGTCAACATGGTGTA
GTTGGTATAGTGTCCACGGGTGGCAACGGGCTCGTTGGTTTTGCTGATGTGAGGGATCTCTTGTGGTTGGATGAAGAGGCCATGGAGCAAGGTGTGTCTG
ACTACATTAAGGGGCTCGGTGACGCATTTGGAACAGGTTTCACTGATGCTGTATCCAGGGAAGTTGAAGCCCTCAGGAACCACCTCATAGGATCTGATGG
AGCAGTGGAAAAAATCCTAAAGAACCTTATTAAGCTGATTTCAGCGTTAGTAATTGTGATTAGGAGCGATTATGATATGGTCACCCTCACAGCAACTTTA
GCCCTGATTGGTTGTCATGGAAGTCCCTGGGCTTGGATTAAAGCCAAAACAGCATCCATTTTAGGTATCCCCATCGCCCAGAAGCAGAGCGCTTCTTGGC
TAAAGAAATTTAATGATATGGCGAGTGCTGCCAAGGGTTTAGAATGGATATCCAACAAAATTAGTAAGTTCATTGACTGGCTCAGGGAGAAGATTGTTCC
AGCAGCTAAAGAGAAAGCAGAATTTTTAACCAATTTGAAGCAATTACCACTATTAGAGAACCAGATCACGAACTTGGAGCAGTCCGCTGCCTCGCAAGAG
GACCTTGAAGCTATGTTTGGGAATGTGTCATACCTCGCCCATTTCTGTCGCAAGTTCCAACCATTATACGCCACAGAGGCCAAGCGAGTCTATGTTCTAG
AGAAGAGAATGAACAATTACATGCAGTTCAAGAGCAAACACCGTATTGAACCTGTATGTCTCATCATTAGAGGCTCACCAGGCACTGGAAAGTCCCTTGC
GACCGGCATCATTGCCCGGGCCATAGCAGACAAGTACCACTCTAGTGTGTACTCACTCCCACCGGATCCTGACCATTTTGACGGGTACAAACAGCAAGTG
GTTACAGTTATGGATGACCTGTGCCAGAATCCTGACGGCAAAGACATGTCATTATTTTGCCAGATGGTATCCACCGTGGATTTTATTCCACCAATGGCTT
CTCTCGAAGAAAAGGGAGTTTCTTTCACATCTAAATTTGTTATCGCATCCACCAACGCCAGCAACATTATAGTGCCCACAGTGTCTGACTCTGACGCCAT
TCGTCGCAGGTTCTACATGGATTGCGACATTGAGGTCACAGACTCATACAAAACAGACTTGGGTAGACTAGACGCTGGGCGGGCTGCTAAGTTATGCTCT
GAAAACAACACCGCAAATTTCAAACGATGCAGCCCACTAGTGTGTGGGAAAGCTATTCAACTTAGAGACAGGAAATCCAAGGTCAGGTATAGCGTGGACA
CAGTGGTCTCTGAACTTATTAGAGAATACAATAGCAGATCCGCTATTGGTAACACAATTGAAGCATTATTCCAAGGCCCACCCAAGTTCAGGCCAATAAG
GATCAGTCTTGAGGAGAAGCCAGCCCCAGACGCTATTAGCGATCTCCTTGCTAGTGTGGATAGCGAGGAAGTGCGCCAATACTGTAGGGAACAAGGCTGG
ATTATCCCTGAAACTCCCACCAATGTTGAACGACATCTTAATAGAGCAGTGCTAGTCGTGCAATCCATCACTACTGTGGTGGCAGTCGTCTCACTGGTGT
ACGTCATTTACAAGCTCTTTGCGGGGTTTCAAGGTGCGTATTCTGGAGCTCCCAAGCAAGTGCTCAAGAAGCCTGTCCTCCGCACGGCAACAGTGCAGGG
TCCAAGCCTTGATTTTGCCCTATCCTTGCTGAGGAGGAACATCAGGCAAGTCCAAACAGACCAAGGGCATTTTACCATGTTGGGTGTCAGGGATCGCCTG
GCTGTTCTCCCGCGGCACTCACAGCCCGGGAAGACTATTTGGGTGGAACACAAACTTGTGAACATCCTTGATGCAGTCGAGCTGGTGGACGAGCAGGGCG
TTAATTTGGAACTCACATTGGTGACACTAGATATTAATGAAAAATTTAGAGATATCACCAAGTTCATTCCAGAGACCATTAGCGGCGCTAGTGATGCAAC
TCTAGTGATCAACACAGAACATATGCCGTCAATGTTTGTCCCTGTGGGGGACGTCGTGCAGTACGGGTTCTTGAACCTCAGTGGAAAGCCAACACATAGG
ACCATGATGTACAATTTCCCTACAAAAGCAGGACAGTGTGGAGGCGTGGTTACATCAGTCGGTAAGATTGTTGGTATTCACATTGGTGGCAACGGGCGCC
AAGGGTTCTGCGCTGGTTTGAAGAGGAGCTACTTTGCGAGTATGCAAGGTGAGATCCAATGGGTGAAGCCTAACAAGGAAACTGGCAGACTAAACATCAA
TGGACCAACTCGCACTAAGTTGGAGCCTAGTGTATTTCATGATGTGTTTGAAGGCAACAAGGAACCAGCAGTTTTAACAAGTAAAGACCCTAGATTGGAG
GTCGACTTTGAACAAGCCCTGTTTTCCAAGTATGTGGGCAATGTTTTACACGAGCCCGATGAATATGTGACTCAAGCTGCCCTCCACTATGCGAATCAAC
TTAAACAATTGGACATAAACACTAGCAAGATGAGCATGGAGGAAGCGTGCTATGGCACTGAAAACCTGGAAGCAATAGACCTCTGCACTAGTGCTGGGTA
TCCATACAGTGCCCTTGGTATCAAGAAAAGAGACATTCTCGACCCCATAACCAGGGATGTGTCTAAGATGAAATTCTACATGGATAAATACGGACTAGAT
CTGCCATACTCTACCTATGTGAAGGATGAACTTAGATCTCTGGATAAAATCAAGAAAGGAAAGTCACGCCTGATAGAGGCCAGCAGCTTGAATGACTCTG
TCTACCTCAGAATGACTTTTGGGCACCTTTACGAGGTGTTTCATGCTAACCCTGGTACTGTGACTGGCTCAGCAGTAGGTTGCAACCCAGACGTGTTTTG
GAGTAAACTACCGATTCTGCTGCCTGGGTCACTCTTTGCCTTTGACTACTCAGGATATGATGCTAGTCTCAGCCCGGTATGGTTCAGGGCTCTAGAAGTT
GTGTTACGGGAGATTGGGTATTCAGAGGAGGCCGTGTCCCTAATAGAAGGAATCAACCACACCCACCATGTGTACCGGAATAAAACATACTGTGTACTTG
GTGGGATGCCCTCAGGGTGCTCTGGTACTTCCATCTTCAATTCAATGATCAACAACATCATCATTAGAACCCTTTTGATCAAAACCTTTAAGGGAATAGA
CMTGGATGAGTTGAACATGGTGGCCTATGGGGACGATGTGCTGGCCAGTTACCCTTTTCCTATTGATTGCCTTGAATTGGCTAAGACTGGCAAAGAGTAT
GGTTTGACCATGACTCCTGCAGACAAATCACCCTGTTTCAATGAAGTAACATGGGAGAATGCTACCTTCCTGAAGAGAGGGTTCTTGCCAGACCACCAAT
TTCCATTCTTAATTCACCCTACGATGCCCATGAGAGAGATCCATGAGTCCATTCGATGGACTAAGGACGCGCGTAACACCCAGGATCACGTGCGCTCCCT
GTGTCTATTGGCATGGCACAATGGTAAGGATGAATATGAAAAGTTTGTGAGTGCAATTAGATCAGTTCCAGTTGGAAAAGCGTTGGCCATTCCTAACTTT
GAGAATCTGAGAAGAAATTGGCTCGAATTGTTTTAATATTACAGTTTAAAGCTGAACCCCACTAGAAATCTGGTCGTGT

## >EV-A71_big plaque isolate (BP2) DE NOVO ASSEMBLY

GCTGTGGGTTGTACCCACTCACAGGGCCCACGTGGCGCTAGCACTCTGGTTCTGCGGAACCTTTGTGCGCCTGTTTTACGCCCCCCCCCCAATTTGCAAC
TTAGAAGCAATACACAACACTGATCAACAGCAGGCATGGCGCACCAGCTATGTCTTGATCAAGCACTTCTGTTTCCCCGGGCCGAGTATCAATAGACTGT
TCACGCGGTTGAAGGAGAAAGCGCCCGTTATCCGGCTAACTACTTCGAGAAACCTAGTAGCACCATTGAAGCTGCAGAGTGCTTCGCTCGGCACTTCCCC
CGTGTAGATCAGGTCGATGAGTCACTGCAATCCCCACGGGCGACCGTGGCAGTGGCTGCGCTGGCGGCCTGCCTATGGGGCAACCCATAGGACGCTCTAA
TGTGGACATGGTGCGAAGAGTCTATTGAGCTAGTTAGTAGTCCTCCGGCCCCTGAATGCGGCTAATCCTAACTGTGGAGCACATGCCTTCAATCCAGAGG
GTAGTGTGTCGTAATGGGCAACTCTGCAGCGGAACCGACTACTTTGGGTGTCCGTGTTTCCTTTTATCTTTACATTGGCTGCTTATGGTGACGATTATAG
AATTGTTACCATATAGCTATTGGATTGGCCATCCGGTGTGCAATAGAGCTATTATATACCTGTTTGTTGGCTTTGTACCACTAACCTTAAAATCTATAAC
CACCCTCGATTTTATATTAACCCTCAATACAATCAAACATGGGCTCACAGGTGTCTACTCAGCGATCCGGCTCCCACGAGAACTCCAATTCAGCTACAGA
AGGCTCCACCATTAATTACACTACCATCAACTATTACAAAGACTCCTATGCTGCGACAGCGGGCAAACAGAGCCTCAAGCAAGACCCTGATAAATTTGCT
AACCCTGTCAAGGACATTTTCACTGAAATGGCTGCACCACTGAAGTCTCCATCCGCTGAGGCTTGTGGTTACAGTGATCGCGTGGCACAACTCACCATTG
GAAACTCCACCATCACTACACAGGAGGCGGCGAATATCATAGTCGGTTATGGTGAGTGGCCCTCATACTGCTCTGATGACGATGCTACAGCGGTGGACAA
GCCAACGCGCCCAGATGTTTCAGTGAATAGGTTTTATACGTTGGATACTAAATTGTGGGAAAAGTCATCCAAGGGGTGGTATTGGAAGTTTCCTGATGTA
CTGACTGAGACCGGAGTCTTTGGCCAGAATGCACAGTTTCACTATTTATATAGGTCAGGATTTTGCATTCATGTGCAATGTAATGCTAGCAAGTTCCATC
AAGGAGCGTTGTTAGTCGCCATACTTCCAGAGTATGTTATAGGGACAGTGGCAGGCGGCACAGGAACTGAGGACAGCCACCCTCCTTACAAACAAACACA
ACCTGGCGCCGATGGTTTTGAGTTGCAGCACCCGTACGTACTCGATGCTGGGATTCCTATATCACAATTAACAGTGTGCCCCCACCAATGGATTAACCTA
CGGACCAATAACTGTGCCACAATAATAGTGCCGTATATGAACACACTGCCTTTCGACTCTGCCCTGAACCATTGCAACTTTGGGCTGTTGGTGGTGCCCA
TTAGCCCACTAGATTTTGACCAAGGGGCAACTCCGGTTATCCCTATTACAATCACTCTAGCTCCAATGTGCTCTGAGTTTGCAGGTCTCAGACAGGCGGT
CACTCAAGGTTTTCCCACCGAGCCAAAACCAGGAACGAATCAATTTTTGACCACCGATGACGGTGTCTCAGCACCCATTTTACCAAATTTCCACCCCACA
CCATGTATTCACATACCCGGTGAAGTCAGAAACCTGCTTGAGTTGTGTCAAGTGGAGACCATTCTTGAGGTTAACAATGTACCCACCAATGCCACCAGTC
TGATGGAAAGGCTACGATTCCCGGTGTCCGCGCAAGCGGGAAAAGGTGAATTGTGTGCCGTGTTTAGGGCCGACCCTGGAAGAGACGGTCCATGGCAATC
AACAATGCTGGGCCAGTTGTGTGGATATTACACCCAGTGGTCAGGATCACTGGAGGTTACTTTTATGTTCACCGGGTCTTTCATGGCCACGGGTAAAATG
CTCATAGCTTATACACCTCCTGGTGGCCCCTTACCCAAAGATCGGGCCACAGCAATGCTGGGCACACATGTTATCTGGGATTTTGGGCTACAATCATCTG
TCACCCTTGTAATACCATGGATTAGCAACACCCACTACAGAGCGCATGCCCGGGATGGAGTGTTCGATTACTATACCACAGGACTGGTTAGTATCTGGTA
TCAAACAAACTACGTGGTTCCAATTGGGGCACCCAACACAGCTTACATAATAGCACTAGCGGCAGCCCAGAAGAATTTTACCATGAAACTGTGTAAAGAC
ACCAGTCACATATTACAGACAGCCTCTATTCAGGGAGATAGAGTGGCAGATGTGATAGAGAGCTCTATAGGAGATAGTGTGAGTAGGGCACTTACCCAGG
CCCTGCCAGCTCCAACAGGTCAGAACACGCAGGTGAGCAGTCATCGACTAGACACTGGTGAAGTTCCAGCGCTCCAAGCTGCTGAAATAGGGGCATCGTC
AAATACTAGTGATGAGAGTATGATTGAGACACGATGCGTTCTTAATTCACACAGTACGGCAGAGACCACCCTGGACAGCTTCTTCAGTAGGGCAGGCTTG
GTAGGAGAGATAGATCTCCCTCTTGAGGGTACCACTAATCCAAGTGGTTATGCTAATTGGGATATAGACATAACTGGTTACGCACAAATGCGCAGGAAAG
TGGAGCTGTTCACCTACATGCGCTTTGATGCGGAATTCACTTTTGTTGCGTGCACTCCTACTGGTCAGGTTGTCCCACAATTACTTCAGTATATGTTTGT
TCCCCCTGGTGCTCCCAAACCAGAGTCTAGAGAATCACTTGCTTGGCAGACAGCCACAAACCCCTCAGTTTTTGTCAAGTTGACTGATCCCCCGGCACAG
GTCTCAGTTCCGTTCATGTCACCCGCGAGCGCTTACCAGTGGTTTTACGACGGGTACCCCACGTTTGGAGAACACAAACAGGAGAAAGACCTTGAGTATG
GAGCGTGCCCTAATAATATGATGGGCACTTTCTCGGTGCGAAATGTGGGTTCATCAAAGTCCAAGTATCCTTTGGTTGTCAGGATATATATGAGAATGAA
GCATGTCAGGGCGTGGATACCTCGCCCGATGCGCAACCAAAACTACCTGTTTAAAGCCAATCCAAACTATGCCGGTGACTCCATCAAACCGACCGGCACT
AGTCGTACTGCCATTACTACCCTTGGAAAGTTCGGCCAGCAATCTGGGGCCATCTACGTGGGCAACTTCAGAGTGGTTAATCGTCACCTCGCTACTCATA
ATGACTGGGCGAACCTCGTCTGGGAAGATAGCTCCCGCGACCTATTAGTGTCGTCTACCACCGCCCAGGGCTGTGATACAATTGCACGTTGTGACTGTCA
AACAGGAGTGTACTATTGTAATTCCAAAAGAAAGCACTATCCAGTCAGCTTCTCCAAACCCAGCCTCATATATGTGGAGGCTAGCGAGTATTACCCTGCT
AGATACCAATCGCACCTGATGCTTGCAGCAGGCCACTCTGAGCCCGGCGACTGCGGGGGCATCTTAAGGTGTCAACATGGTGTAGTTGGTATAGTGTCCA
CGGGTGGCAACGGGCTCGTTGGTTTTGCTGATGTGAGGGATCTCTTGTGGTTGGATGAAGAGGCCATGGAGCAAGGTGTGTCTGACTACATTAAGGGCCT
CGGTGACGCATTTGGAACAGGTTTCACTGATGCTGTATCCAGGGAAGTTGAAGCCCTCAGGAACCACCTCATAGGATCTGATGGAGCAGTGGAAAAAATC
CTAAAGAACCTTATTAAGCTGATTTCAGCGTTAGTAATTGTGATTAGGAGCGATTATGATATGGTCACCCTCACAGCAACTTTAGCCCTGATTGGTTGTC
ATGGAAGTCCCTGGGCTTGGATTAAAGCCAAAACAGCATCCATTTTAGGTATCCCCATCGCCCAGAAGCAGAGCGCTTCTTGGCTAAAGAAATTTAATGA
TATGGCGAGTGCTGCCAAGGGTTTAGAATGGATATCCAACAAAATTAGTAAGTTCATTGACTGGCTCAGGGAGAAGATTGTTCCAGCAGCTAAAGAGAAA
GCAGAATTTTTAACCAATTTGAAGCAATTACCACTATTAGAGAACCAGATCACGAACTTGGAGCAGTCCGCTGCCTCGCAAGAGGACCTTGAAGCTATGT
TTGGGAATGTGTCATACCTCGCCCATTTCTGTCGCAAGTTCCAACCATTATACGCCACAGAGGCCAAGCGAGTCTATGTTCTAGAGAAGAGAATGAACAA
TTACATGCAGTTCAAGAGCAAACACCGTATTGAACCTGTATGTCTCATCATTAGAGGCTCACCAGGCACTGGAAAGTCCCTTGCGACCGGCATCATTGCC
CGGGCCATAGCAGACAAGTACCACTCTAGTGTGTACTCACTCCCACCGGATCCTGACCATTTTGACGGGTACAAACAGCAAGTGGTTACAGTTATGGATG
ACCTGTGCCAGAATCCTGACGGCAAAGACATGTCATTATTTTGCCAGATGGTATCCACCGTGGATTTTATTCCACCAATGGCTTCTCTCGAAGAAAAGGG
AGTTTCTTTCACATCTAAATTTGTTATCGCATCCACCAACGCCAGCAACATTATAGTGCCCACAGTGTCTGACTCTGACGCCATTCGTCGCAGGTTCTAC
ATGGATTGCGACATTGAGGTCACAGACTCATACAAAACAGACTTGGGTAGACTAGACGCTGGGCGGGCTGCTAAGTTATGCTCTGAAAACAACACCGCAA
ATTTCAAACGATGCAGCCCACTAGTGTGTGGGAAAGCTATTCAACTTAGAGACAGGAAATCCAAGGTCAGGTATAGCGTGGACACAGTGGTCTCTGAACT
TATTAGAGAATACAATAGCAGATCCGCTATTGGTAACACAATTGAAGCATTATTCCAAGGCCCACCCAAGTTCAGGCCAATAAGGATCAGTCTTGAGGAG
AAGCCAGCCCCAGACGCTATTAGCGATCTCCTTGCTAGTGTGGATAGCGAGGAAGTGCGCCAATACTGTAGGGAACAAGGCTGGATTATCCCTGAAACTC
CCACCAATGTTGAACGACATCTTAATAGAGCAGTGCTAGTCGTGCAATCCATCACTACTGTGGTGGCAGTCGTCTCACTGGTGTACGTCATTTACAAGCT
CTTTGCGGGGTTTCAAGGTGCGTATTCTGGAGCTCCCAAGCAAGTGCTCAAGAAGCCTGTCCTCCGCACGGCAACAGTGCAGGGTCCAAGCCTTGATTTT
GCCCTATCCTTGCTGAGGAGGAACATCAGGCAAGTCCAAACAGACCAAGGGCATTTTACCATGTTGGGTGTCAGGGATCGCCTGGCTGTTCTCCCGCGGC
ACTCACAGCCCGGGAAGACTATTTGGGTGGAACACAAACTTGTGAACATCCTTGATGCAGTCGAGCTGGTGGACGAGCAGGGCGTTAATTTGGAACTCAC
ATTGGTGACACTAGATATTAATGAAAAATTTAGAGATATCACCAAGTTCATTCCAGAGACCATTAGCGGCGCTAGTGATGCAACTCTAGTGATCAACACA
GAACATATGCCGTCAATGTTTGTCCCTGTGGGGGACGTCGTGCAGTACGGGTTCTTGAACCTCAGTGGAAAGCCAACACATAGGACCATGATGTACAATT
TCCCTACAAAAGCAGGACAGTGTGGAGGCGTGGTTACATCAGTCGGTAAGATTGTTGGTATTCACATTGGTGGCAACGGGCGCCAAGGGTTCTGCGCTGG
TTTGAAGAGGAGCTACTTTGCGAGTATGCAAGGTGAGATCCAATGGGTGAAGCCTAACAAGGAAACTGGCAGACTAAACATCAATGGACCAACTCGCACT
AAGTTGGAGCCTAGTGTATTTCATGATGTGTTTGAAGGCAACAAGGAACCAGCAGTTTTAACAAGTAAAGACCCTAGATTGGAGGTCGACTTTGAACAAG
CCCTGTTTTCCAAGTATGTGGGCAATGTTTTACACGAGCCCGATGAATATGTGACTCAAGCTGCCCTCCACTATGCGAATCAACTTAAACAATTGGACAT
AAACACTAGCAAGATGAGCATGGAGGAAGCGTGCTATGGCACTGAAAACCTGGAAGCAATAGACCTYTGCACTAGTGCTGGGTATCCATACAGTGCCCTT
GGTATCAAGAAAAGAGACATTCTCGACCCCATAACCAGGGATGTGTCTAAGATGAAATTCTACATGGATAAATACGGACTAGATCTGCCATACTCTACCT
ATGTGAAGGATGAACTTAGATCTCTGGATAAAATCAAGAAAGGAAAGTCACGCCTGATAGAGGCCAGCAGCTTGAATGACTCTGTCTACCTCAGAATGAC
TTTTGGGCACCTTTACGAGGTGTTTCATGCTAACCCTGGTACTGTGACTGGCTCAGCAGTAGGTTGCAACCCAGACGTGTTTTGGAGTAAACTACCGATT
CTGCTGCCTGGGTCACTCTTTGCCTTTGACTACTCAGGATATGATGCTAGTCTCAGCCCGGTATGGTTCAGGGCTCTAGAAGTTGTGTTACGGGAGATTG
GGTATTCAGAGGAGGCCGTGTCCCTAATAGAAGGAATCAACCACACCCACCATGTGTACCGGAATAAAACATACTGTGTACTTGGTGGGATGCCCTCAGG
GTGCTCTGGTACTTCCATCTTCAATTCAATGATCAACAACATCATCATTAGAACCCTTTTGATCAAAACCTTTAAGGGAATAGACCTGGATGAGTTGAAC
ATGGTGGCCTATGGGGACGATGTGCTGGCCAGTTACCCTTTTCCTATTGATTGCCTTGAATTGGCTAAGACTGGCAAAGAGTATGGTTTGACCATGACTC
CTGCAGACAAATCACCCTGTTTCAATGAAGTAACATGGGAGAATGCTACCTTCCTGAAGAGAGGGTTCTTGCCAGACCACCAATTTCCATTCTTAATTCA
CCCTACGATGCCCATGAGAGAGATCCATGAGTCCATTCGATGGACTAAGGACGCGCGTAACACCCAGGATCACGTGCGCTCCCTGTGTCTATTGGCATGG
CACAATGGTAAGGATGAATATGAAAAGTTTGTGAGTGCAATTAGATCAGTTCCAGTTGGAAAAGCGTTGGCCATTCCTAACTTTGAGAATCTGAGAAGAA
ATTGGCTCGAATTGTTTTAATATTACAGTTTAAAGCTGAACCCCACTAGAAATCTGGTCG

**>EV-A71_big plaque isolate (BP3) DE NOVO ASSEMBLY**

```
---TTTTAAAACA--
GGCTGTGGGTTGTACCCACTCACAGGGCCCACGTGGCGCTAGCACTCTGGTTCTGCGGAACCTTTGTGCGCCTGTTTTACGCCCCCCCCCCAATTTGCAA
CTTAGAAGCAATACACAACACTGATCAACAGCAGGCATGGCGCACCAGCTATGTCTTGATCAAGCACTTCTGTTTCCCCGGGCCGAGTATCAATAGACTG
TTCACGCGGTTGAAGGAGAAAGCGCCCGTTATCCGGCTAACTACTTCGAGAAACCTAGTAGCACCATTGAAGCTGCAGAGTGCTTCGCTCGGCACTTCCC
CCGTGTAGATCAGGTCGATGAGTCACTGCAATCCCCACGGGCGACCGTGGCAGTGGCTGCGCTGGCGGCCTGCCTATGGGGCAACCCATAGGACGCTCTA
ATGTGGACATGGTGCGAAGAGTCTATTGAGCTAGTTAGTAGTCCTCCGGCCCCTGAATGCGGCTAATCCTAACTGTGGAGCACATGCCTTCAATCCAGAG
GGTAGTGTGTCGTAATGGGCAACTCTGCAGCGGAACCGACTACTTTGGGTGTCCGTGTTTCCTTTTATCTTTACATTGGCTGCTTATGGTGACGATTATA
GAATTGTTACCATATAGCTATTGGATTGGCCATCCGGTGTGCAATAGAGCTATTATATACCTGTTTGTTGGCTTTGTACCACTAACCTTAAAATCTATAA
CCACCCTCGATTTTATATTAACCCTCAATACAATCAAACATGGGCTCACAGGTGTCTACTCAGCGATCCGGCTCCCACGAGAACTCCAATTCAGCTACAG
AAGGCTCCACCATTAATTACACTACCATCAACTATTACAAAGACTCCTATGCTGCGACAGCGGGCAAACAGAGCCTCAAGCAAGACCCTGATAAATTTGC
TAACCCTGTCAAGGACATTTTCACTGAAATGGCTGCACCACTGAAGTCTCCATCCGCTGAGGCTTGTGGTTACAGTGATCGCGTGGCACAACTCACCATT
GGAAACTCCACCATCACTACACAGGAGGCGGCGAATATCATAGTCGGTTATGGTGAGTGGCCCTCATACTGCTCTGATGACGATGCTACAGCGGTGGACA
AGCCAACGCGCCCAGATGTTTCAGTGAATAGGTTTTATACGTTGGATACTAAATTGTGGGAAAAGTCATCCAAGGGGTGGTATTGGAAGTTTCCTGATGT
ACTGACTGAGACCGGAGTCTTTGGCCAGAATGCACAGTTTCACTATTTATATAGGTCAGGATTTTGCATTCATGTGCAATGTAATGCTAGCAAGTTCCAT
CAAGGAGCGTTGTTAGTCGCCATACTTCCAGAGTATGTTATAGGGACAGTGGCAGGCGGCACAGGAACTGAGGACAGCCACCCTCCTTACAAACAAACAC
AACCTGGCGCCGATGGTTTTGAGTTGCAGCACCCGTACGTACTCGATGCTGGGATTCCTATATCACAATTAACAGTGTGCCCCCACCAATGGATTAACCT
ACGGACCAATAACTGTGCCACAATAATAGTGCCGTATATGAACACACTGCCTTTCGACTCTGCCCTGAACCATTGCAACTTTGGGCTGTTGGTGGTGCCC
ATTAGCCCACTAGATTTTGACCAAGGGGCAACTCCGGTTATCCCTATTACAATCACTCTAGCTCCAATGTGCTCTGAGTTTGCAGGTCTCAGACAGGCGG
TCACTCAAGGTTTTCCCACCGAGCCAAAACCAGGAACGAATCAATTTTTGACCACCGATGACGGTGTCTCAGCACCCATTTTACCAAATTTCCACCCCAC
ACCATGTATTCACATACCCGGTGAAGTCAGAAACCTGCTTGAGTTGTGTCAAGTGGAGACCATTCTTGAGGTTAACAATGTACCCACCAATGCCACCAGT
CTGATGGAAAGGCTACGATTCCCGGTGTCCGCGCAAGCGGGAAAAGGTGAATTGTGTGCCGTGTTTAGGGCCGACCCTGGAAGAGACGGTCCATGGCAAT
CAACAATGCTGGGCCAGTTGTGTGGATATTACACCCAGTGGTCAGGATCACTGGAGGTTACTTTTATGTTCACCGGGTCTTTCATGGCCACGGGTAAAAT
GCTCATAGCTTATACACCTCCTGGTGGCCCCTTACCCAAAGATCGGGCCACAGCAATGCTGGGCACACATGTTATCTGGGATTTTGGGCTACAATCATCT
GTCACCCTTGTAATACCATGGATTAGCAACACCCACTACAGAGCGCATGCCCGGGATGGAGTGTTCGATTACTATACCACAGGACTGGTTAGTATCTGGT
ATCAAACAAACTACGTGGTTCCAATTGGGGCACCCAACACAGCTTACATAATAGCACTAGCGGCAGCCCAGAAGAATTTTACCATGAAACTGTGTAAAGA
CACCAGTCACATATTACAGACAGCCTCTATTCAGGGAGATAGAGTGGCAGATGTGATAGAGAGCTCTATAGGAGATAGTGTGAGTAGGGCACTTACCCAG
GCCCTGCCAGCTCCAACAGGTCAGAACACGCAGGTGAGCAGTCATCGACTAGACACTGGTGAAGTTCCAGCGCTCCAAGCTGCTGAAATAGGGGCATCGT
CAAATACTAGTGATGAGAGTATGATTGAGACACGATGCGTTCTTAATTCACACAGTACGGCAGAGACCACCCTGGACAGCTTCTTCAGTAGGGCAGGCTT
GGTAGGAGAGATAGATCTCCCTCTTGAGGGTACCACTAATCCAAGTGGTTATGCTAATTGGGATATAGACATAACTGGTTACGCACAAATGCGCAGGAAA
GTGGAGCTGTTCACCTACATGCGCTTTGATGCGGAATTCACTTTTGTTGCGTGCACTCCTACTGGTCAGGTTGTCCCACAATTACTTCAGTATATGTTTG
TTCCCCCTGGTGCTCCCAAACCAGAGTCTAGAGAATCACTTGCTTGGCAGACAGCCACAAACCCCTCAGTTTTTGTCAAGTTGACTGATCCCCCGGCACA
GGTCTCAGTTCCGTTCATGTCACCCGCGAGCGCTTACCAGTGGTTTTACGACGGGTACCCCACGTTTGGAGAACACAAACAGGAGAAAGACCTTGAGTAT
GGAGCGTGCCCTAATAATATGATGGGCACTTTCTCGGTGCGAAATGTGGGTTCATCAAAGTCCAAGTATCCTTTGGTTGTCAGGATATATATGAGAATGA
AGCATGTCAGGGCGTGGATACCTCGCCCGATGCGCAACCAAAACTACCTGTTTAAAGCCAATCCAAACTATGCCGGTGACTCCATCAAACCGACCGGCAC
TAGTCGTACTGCCATTACTACCCTTGGAAAGTTCGGCCAGCAATCTGGGGCCATCTACGTGGGCAACTTCAGAGTGGTTAATCGTCACCTCGCTACTCAT
AATGACTGGGCGAACCTCGTCTGGGAAGATAGCTCCCGCGACCTATTAGTGTCGTCTACCACCGCCCAGGGCTGTGATACAATTGCACGTTGTGACTGTC
AAACAGGAGTGTACTATTGTAATTCCAAAAGAAAGCACTATCCAGTCAGCTTCTCCAAACCCAGCCTCATATATGTGGAGGCTAGCGAGTATTACCCTGC
TAGATACCAATCGCACCTGATGCTTGCAGCAGGCCACTCTGAGCCCGGCGACTGCGGGGGCATCTTAAGGTGTCAACATGGTGTAGTTGGTATAGTGTCC
ACGGGTGGCAACGGGCTCGTTGGTTTTGCTGATGTGAGGGATCTCTTGTGGTTGGATGAAGAGGCCATGGAGCAAGGTGTGTCTGACTACATTAAGGGGC
TCGGTGACGCATTTGGAACAGGTTTCACTGATGCTGTATCCAGGGAAGTTGAAGCCCTCAGGAACCACCTCATAGGATCTGATGGAGCAGTGGAAAAAAT
CCTAAAGAACCTTATTAAGCTGATTTCAGCGTTAGTAATTGTGATTAGGAGCGATTATGATATGGTCACCCTCACAGCAACTTTAGCCCTGATTGGTTGT
CATGGAAGTCCCTGGGCTTGGATTAAAGCCAAAACAGCATCCATTTTAGGTATCCCCATCGCCCAGAAGCAGAGCGCTTCTTGGCTAAAGAAATTTAATG
ATATGGCGAGTGCTGCCAAGGGTTTAGAATGGATATCCAACAAAATTAGTAAGTTCATTGACTGGCTCAGGGAGAAGATTGTTCCAGCAGCTAAAGAGAA
AGCAGAATTTTTAACCAATTTGAAGCAATTACCACTATTAGAGAACCAGATCACGAACTTGGAGCAGTCCGCTGCCTCGCAAGAGGACCTTGAAGCTATG
TTTGGGAATGTGTCATACCTCGCCCATTTCTGTCGCAAGTTCCAACCATTATACGCCACAGAGGCCAAGCGAGTCTATGTTCTAGAGAAGAGAATGAACA
ATTACATGCAGTTCAAGAGCAAACACCGTATTGAACCTGTATGTCTCATCATTAGAGGCTCACCAGGCACTGGAAAGTCCCTTGCGACCGGCATCATTGC
CCGGGCCATAGCAGACAAGTACCACTCTAGTGTGTACTCACTCCCACCGGATCCTGACCATTTTGACGGGTACAAACAGCAAGTGGTTACAGTTATGGAT
GACCTGTGCCAGAATCCTGACGGCAAAGACATGTCATTATTTTGCCAGATGGTATCCACCGTGGATTTTATTCCACCAATGGCTTCTCTCGAAGAAAAGG
GAGTTTCTTTCACATCTAAATTTGTTATCGCATCCACCAACGCCAGCAACATTATAGTGCCCACAGTGTCTGACTCTGACGCCATTCGTCGCAGGTTCTA
CATGGATTGCGACATTGAGGTCACAGACTCATACAAAACAGACTTGGGTAGACTAGACGCTGGGCGGGCTGCTAAGTTATGCTCTGAAAACAACACCGCA
AATTTCAAACGATGCAGCCCACTAGTGTGTGGGAAAGCTATTCAACTTAGAGACAGGAAATCCAAGGTCAGGTATAGCGTGGACACAGTGGTCTCTGAAC
TTATTAGAGAATACAATAGCAGATCCGCTATTGGTAACACAATTGAAGCATTATTCCAAGGCCCACCCAAGTTCAGGCCAATAAGGATCAGTCTTGAGGA
GAAGCCAGCCCCAGACGCTATTAGCGATCTCCTTGCTAGTGTGGATAGCGAGGAAGTGCGCCAATACTGTAGGGAACAAGGCTGGATTATCCCTGAAACT
CCCACCAATGTTGAACGACATCTTAATAGAGCAGTGCTAGTCGTGCAATCCATCACTACTGTGGTGGCAGTCGTCTCACTGGTGTACGTCATTTACAAGC
TCTTTGCGGGGTTTCAAGGTGCGTATTCTGGAGCTCCCAAGCAAGTGCTCAAGAAGCCTGTCCTCCGCACGGCAACAGTGCAGGGTCCAAGCCTTGATTT
TGCCCTATCCTTGCTGAGGAGGAACATCAGGCAAGTCCAAACAGACCAAGGGCATTTTACCATGTTGGGTGTCAGGGATCGCCTGGCTGTTCTCCCGCGG
CACTCACAGCCCGGGAAGACTATTTGGGTGGAACACAAACTTGTGAACATCCTTGATGCAGTCGAGCTGGTGGACGAGCAGGGCGTTAATTTGGAACTCA
CATTGGTGACACTAGATATTAATGAAAAATTTAGAGATATCACCAAGTTCATTCCAGAGACCATTAGCGGCGCTAGTGATGCAACTCTAGTGATCAACAC
AGAACATATGCCGTCAATGTTTGTCCCTGTGGGGGACGTCGTGCAGTACGGGTTCTTGAACCTCAGTGGAAAGCCAACACATAGGACCATGATGTACAAT
TTCCCTACAAAAGCAGGACAGTGTGGAGGCGTGGTTACATCAGTCGGTAAGATTGTTGGTATTCACATTGGTGGCAACGGGCGCCAAGGGTTCTGCGCTG
GTTTGAAGAGGAGCTACTTTGCGAGTATGCAAGGTGAGATCCAATGGGTGAAGCCTAACAAGGAAACTGGCAGACTAAACATCAATGGACCAACTCGCAC
TAAGTTGGAGCCTAGTGTATTTCATGATGTGTTTGAAGGCAACAAGGAACCAGCAGTTTTAACAAGTAAAGACCCTAGATTGGAGGTCGACTTTGAACAA
GCCCTGTTTTCCAAGTATGTGGGCAATGTTTTACACGAGCCCGATGAATATGTGACTCAAGCTGCCCTCCACTATGCGAATCAACTTAAACAATTGGACA
TAAACACTAGCAAGATGAGCATGGAGGAAGCGTGCTATGGCACTGAAAACCTGGAAGCAATAGACCTCTGCACTAGTGCTGGGTATCCATACAGTGCCCT
TGGTATCAAGAAAAGAGACATTCTCGACCCCATAACCAGGGATGTGTCTAAGATGAAATTCTACATGGATAAATACGGACTAGATCTGCCATACTCTACC
TATGTGAAGGATGAACTTAGATCTCTGGATAAAATCAAGAAAGGAAAGTCACGCCTGATAGAGGCCAGCAGCTTGAATGACTCTGTCTACCTCAGAATGA
CTTTTGGGCACCTTTACGAGGTGTTTCATGCTAACCCTGGTACTGTGACTGGCTCAGCAGTAGGTTGCAACCCAGACGTGTTTTGGAGTAAACTACCGAT
TCTGCTGCCTGGGTCACTCTTTGCCTTTGACTACTCAGGATATGATGCTAGTCTCAGCCCGGTATGGTTCAGGGCTCTAGAAGTTGTGTTACGGGAGATT
GGGTATTCAGAGGAGGCCGTGTCCCTAATAGAAGGAATCAACCACACCCACCATGTGTACCGGAATAAAACATACTGTGTACTTGGTGGGATGCCCTCAG
GGTGCTCTGGTACTTCCATCTTCAATTCAATGATCAACAACATCATCATTAGAACCCTTTTGATCAAAACCTTTAAGGGAATAGACCTGGATGAGTTGAA
CATGGTGGCCTATGGGGACGATGTGCTGGCCAGTTACCCTTTTCCTATTGATTGCCTTGAATTGGCTAAGACTGGCAAAGAGTATGGTTTGACCATGACT
CCTGCAGACAAATCACCCTGTTTCAATGAAGTAACATGGGAGAATGCTACCTTCCTGAAGAGAGGGTTCTTGCCAGACCACCAATTTCCATTCTTAATTC
ACCCTACGATGCCCATGAGAGAGATCCATGAGTCCATTCGATGGACTAAGGACGCGCGTAACACCCAGGATCACGTGCGCTCCCTGTGTCTATTGGCATG
GCACAATGGTAAGGATGAATATGAAAAGTTTGTGAGTGCAATTAGATCAGTTCCAGTTGGAAAAGCGTTGGCCATTCCTAACTTTGAGAATCTGAGAAGA
AATTGGCTCGAATTGTTTTAATATTACAGTTTAAAGCTGAACCCCACTAGAAA
```

# >EV-A71_big plaque isolate (BP4) DE NOVO ASSEMBLY

GGCTGTGGGTTGTACCCACTCACAGGGCCCACGTGGCGCTAGCACTCTGGTTCTGCGGAACCTTTGTGCGCCTGTTTTACGCCCCCCCCCCAATTTGCAA
CTTAGAAGCAATACACAACACTGATCAACAGCAGGCATGGCGCACCAGCTATGTCTTGATCAAGCACTTCTGTTTCCCCGGGCCGAGTATCAATAGACTG
TTCACGCGGTTGAAGGAGAAAGCGCCCGTTATCCGGCTAACTACTTCGAGAAACCTAGTAGCACCATTGAAGCTGCAGAGTGCTTCGCTCGGCACTTCCC
CCGTGTAGATCAGGTCGATGAGTCACTGCAATCCCCACGGGCGACCGTGGCAGTGGCTGCGCTGGCGGCCTGCCTATGGGGCAACCCATAGGACGCTCTA
ATGTGGACATGGTGCGAAGAGTCTATTGAGCTAGTTAGTAGTCCTCCGGCCCCTGAATGCGGCTAATCCTAACTGTGGAGCACATGCCTTCAATCCAGAG
GGTAGTGTGTCGTAATGGGCAACTCTGCAGCGGAACCGACTACTTTGGGTGTCCGTGTTTCCTTTTATCTTTACATTGGCTGCTTATGGTGACGATTATA
GAATTGTTACCATATAGCTATTGGATTGGCCATCCGGTGTGCAATAGAGCTATTATATACCTGTTTGTTGGCTTTGTACCACTAACCTTAAAATCTATAA
CCACCCTCGATTTTATATTAACCCTCAATACAATCAAACATGGGCTCACAGGTGTCTACTCAGCGATCCGGCTCCCACGAGAACTCCAATTCAGCTACAG
AAGGCTCCACCATTAATTACACTACCATCAACTATTACAAAGACTCCTATGCTGCGACAGCGGGCAAACAGAGCCTCAAGCAAGACCCTGATAAATTTGC
TAACCCTGTCAAGGACATTTTCACTGAAATGGCTGCACCACTGAAGTCTCCATCCGCTGAGGCTTGTGGTTACAGTGATCGCGTGGCACAACTCACCATT
GGAAACTCCACCATCACTACACAGGAGGCGGCGAATATCATAGTCGGTTATGGTGAGTGGCCCTCATACTGCTCTGATGACGATGCTACAGCGGTGGACA
AGCCAACGCGCCCAGATGTTTCAGTGAATAGGTTTTATACGTTGGATACTAAATTGTGGGAAAAGTCATCCAAGGGGTGGTATTGGAAGTTTCCTGATGT
ACTGACTGAGACCGGAGTCTTTGGCCAGAATGCACAGTTTCACTATTTATATAGGTCAGGATTTTGCATTCATGTGCAATGTAATGCTAGCAAGTTCCAT
CAAGGAGCGTTGTTAGTCGCCATACTTCCAGAGTATGTTATAGGGACAGTGGCAGGCGGCACAGGAACTGAGGACAGCCACCCTCCTTACAAACAAACAC
AACCTGGCGCCGATGGTTTTGAGTTGCAGCACCCGTACGTACTCGATGCTGGGATTCCTATATCACAATTAACAGTGTGCCCCCACCAATGGATTAACCT
ACGGACCAATAACTGTGCCACAATAATAGTGCCGTATATGAACACACTGCCTTTCGACTCTGCCCTGAACCATTGCAACTTTGGGCTGTTGGTGGTGCCC
ATTAGCCCACTAGATTTTGACCAAGGGGCAACTCCGGTTATCCCTATTACAATCACTCTAGCTCCAATGTGCTCTGAGTTTGCAGGTCTCAGACAGGCGG
TCACTCAAGGTTTTCCCACCGAGCCAAAACCAGGAACGAATCAATTTTTGACCACCGATGACGGTGTCTCAGCACCCATTTTACCAAATTTCCACCCCAC
ACCATGTATTCACATACCCGGTGAAGTCAGAAACCTGCTTGAATTGTGTCAAGTGGAGACCATTCTTGAGGTTAACAATGTACCCACCAATGCCACCAGT
CTGATGGAAAGGCTACGATTCCCGGTGTCCGCGCAAGCGGGAAAAGGTGAATTGTGTGCCGTGTTTAGGGCCGACCCTGGAAGAGACGGTCCATGGCAAT
CAACAATGCTGGGCCAGTTGTGTGGATATTACACCCAGTGGTCAGGATCACTGGAGGTTACTTTTATGTTCACCGGGTCTTTCATGGCCACGGGTAAAAT
GCTCATAGCTTATACACCTCCTGGTGGCCCCTTACCCAAAGATCGGGCCACAGCAATGCTGGGCACACATGTTATCTGGGATTTTGGGCTACAATCATCT
GTCACCCTTGTAATACCATGGATTAGCAACACCCACTACAGAGCGCATGCCCGGGATGGAGTGTTCGATTACTATACCACAGGACTGGTTAGTATCTGGT
ATCAAACAAACTACGTGGTTCCAATTGGGGCACCCAACACAGCTTACATAATAGCACTAGCGGCAGCCCAGAAGAATTTTACCATGAAACTGTGTAAAGA
CACCAGTCACATATTACAGACAGCCTCTATTCAGGGAGATAGAGTGGCAGATGTGATAGAGAGCTCTATAGGAGATAGTGTGAGCAGGGCACTTACCCAG
GCCCTGCCAGCTCCAACAGGTCAGAACACGCAGGTGAGCAGTCATCGACTAGACACTGGTGAAGTTCCAGCGCTCCAAGCTGCTGAAATAGGGGCATCGT
CAAATACTAGTGATGAGAGTATGATTGAGACACGATGCGTTCTTAATTCACACAGTACGGCAGAGACCACCCTGGACAGCTTCTTCAGTAGGGCAGGCTT
GGTAGGAGAGATAGATCTCCCTCTTGAGGGTACCACTAATCCAAGTGGTTATGCTAATTGGGATATAGACATAACTGGTTACGCACAAATGCGCAGGAAA
GTGGAGCTGTTCACCTACATGCGCTTTGATGCGGAATTCACTTTTGTTGCGTGCACTCCTACTGGTCAGGTTGTCCCACAATTACTTCAGTATATGTTTG
TTCCCCCTGGTGCTCCCAAACCAGAGTCTAGAGAATCACTTGCTTGGCAGACAGCCACAAACCCCTCAGTTTTTGTCAAGTTGACTGATCCCCCGGCACA
GGTCTCAGTTCCGTTCATGTCACCCGCGAGCGCTTACCAGTGGTTTTACGACGGGTACCCCACGTTTGGAGAACACAAACAGGAGAAAGACCTTGAGTAT
GGAGCGTGCCCTAATAATATGATGGGCACTTTCTCGGTGCGAAATGTGGGTTCATCAAAGTCCAAGTATCCTTTGGTTGTCAGGATATATATGAGAATGA
AGCATGTCAGGGCGTGGATACCTCGCCCGATGCGCAACCAAAACTACCTGTTTAAAGCCAATCCAAACTATGCCGGTGACTCCATCAAACCGACCGGCAC
TAGTCGTACTGCCATTACTACCCTTGGAAAGTTCGGCCAGCAATCTGGGGCCATCTACGTGGGCAACTTCAGAGTGGTTAATCGTCACCTCGCTACTCAT
AATGACTGGGCGAACCTCGTCTGGGAAGATAGCTCCCGCGACCTATTAGTGTCGTCTACCACCGCCCAGGGCTGTGATACAATTGCACGTTGTGACTGTC
AAACAGGAGTGTACTATTGTAATTCCAAAAGAAAGCACTATCCAGTCAGCTTCTCCAAACCCAGCCTCATATATGTGGAGGCTAGCGAGTATTACCCTGC
TAGATACCAATCGCACCTGATGCTTGCAGCAGGCCACTCTGAGCCCGGCGACTGCGGGGGCATCTTAAGGTGTCAACATGGTGTAGTTGGTATAGTGTCC
ACGGGTGGCAACGGGCTCGTTGGTTTTGCTGATGTGAGGGATCTCTTGTGGTTGGATGAAGAGGCCATGGAGCAAGGTGTGTCTGACTACATTAAGGGGC
TCGGTGACGCATTTGGAACAGGTTTCACTGATGCTGTATCCAGGGAAGTTGAAGCCCTCAGGAACCACCTCATAGGATCTGATGGAGCAGTGGAAAAAAT
CCTAAAGAACCTTATTAAGCTGATTTCAGCGTTAGTAATTGTGATTAGGAGCGATTATGATATGGTCACCCTCACAGCAACTTTAGCCCTGATTGGTTGT
CATGGAAGTCCCTGGGCTTGGATTAAAGCCAAAACAGCATCCATTTTAGGTATCCCCATCGCCCAGAAGCAGAGCGCTTCTTGGCTAAAGAAATTTAATG
ATATGGCGAGTGCTGCCAAGGGTTTAGAATGGATATCCAACAAAATTAGTAAGTTCATTGACTGGCTCAGGGAGAAGATTGTTCCAGCAGCTAAAGAGAA
AGCAGAATTTTTAACCAATTTGAAGCAATTACCACTATTAGAGAACCAGATCACGAACTTGGAGCAGTCCGCTGCCTCGCAAGAGGACCTTGAAGCTATG
TTTGGGAATGTGTCATACCTCGCCCATTTCTGTCGCAAGTTCCAACCATTATACGCCACAGAGGCCAAGCGAGTCTATGTTCTAGAGAAGAGAATGAACA
ATTACATGCAGTTCAAGAGCAAACACCGTATTGAACCTGTATGTCTCATCATTAGAGGCTCACCAGGCACTGGAAAGTCCCTTGCGACCGGCATCATTGC
CCGGGCCATAGCAGACAAGTACCACTCTAGTGTGTACTCACTCCCACCGGATCCTGACCATTTTGACGGGTACAAACAGCAAGTGGTTACAGTTATGGAT
GACCTGTGCCAGAATCCTGACGGCAAAGACATGTCATTATTTTGCCAGATGGTATCCACCGTGGATTTTATTCCACCAATGGCTTCTCTCGAAGAAAAGG
GAGTTTCTTTCACATCTAAATTTGTTATCGCATCCACCAACGCCAGCAACATTATAGTGCCCACAGTGTCTGACTCTGACGCCATTCGTCGCAGGTTCTA
CATGGATTGCGACATTGAGGTCACAGACTCATACAAAACAGACTTGGGTAGACTAGACGCTGGGCGGGCTGCTAAGTTATGCTCTGAAAACAACACCGCA
AATTTCAAACGATGCAGCCCACTAGTGTGTGGGAAAGCTATTCAACTTAGAGACAGGAAATCCAAGGTCAGGTATAGCGTGGACACAGTGGTCTCTGAAC
TTATTAGAGAATACAATAGCAGATCCGCTATTGGTAACACAATTGAAGCATTATTCCAAGGCCCACCCAAGTTCAGGCCAATAAGGATCAGTCTTGAGGA
GAAGCCAGCCCCAGACGCTATTAGCGATCTCCTTGCTAGTGTGGATAGCGAGGAAGTGCGCCAATACTGTAGGGAACAAGGCTGGATTATCCCTGAAACT
CCCACCAATGTTGAACGACATCTTAATAGAGCAGTGCTAGTCGTGCAATCCATCACTACTGTGGTGGCAGTCGTCTCACTGGTGTACGTCATTTACAAGC
TCTTTGCGGGGTTTCAAGGTGCGTATTCTGGAGCTCCCAAGCAAGTGCTCAAGAAGCCTGTCCTCCGCACGGCAACAGTGCAGGGTCCAAGCCTTGATTT
TGCCCTATCCTTGCTGAGGAGGAACATCAGGCAAGTCCAAACAGACCAAGGGCATTTTACCATGTTGGGTGTCAGGGATCGCCTGGCTGTTCTCCCGCGG
CACTCACAGCCCGGGAAGACTATTTGGGTGGAACACAAACTTGTGAACATCCTTGATGCAGTCGAGCTGGTGGACGAGCAGGGCGTTAATTTGGAACTCA
CATTGGTGACACTAGATATTAATGAAAAATTTAGAGATATCACCAAGTTCATTCCAGAGACCATTAGCGGCGCTAGTGATGCAACTCTAGTGATCAACAC
AGAACATATGCCGTCAATGTTTGTCCCTGTGGGGGACGTCGTGCAGTACGGGTTCTTGAACCTCAGTGGAAAGCCAACACATAGGACCATGATGTACAAT
TTCCCTACAAAAGCAGGACAGTGTGGAGGCGTGGTTACATCAGTCGGTAAGATTGTTGGTATTCACATTGGTGGCAACGGGCGCCAAGGGTTCTGCGCTG
GTTTGAAGAGGAGCTACTTTGCGAGTATGCAAGGTGAGATCCAATGGGTGAAGCCTAACAAGGAAACTGGCAGACTAAACATCAATGGACCAACTCGCAC
TAAGTTGGAGCCTAGTGTATTTCATGATGTGTTTGAAGGCAACAAGGAACCAGCAGTTTTAACAAGTAAAGACCCTAGATTGGAGGTCGACTTTGAACAA
GCCCTGTTTTCCAAGTATGTGGGCAATGTTTTACACGAGCCCGATGAATATGTGACTCAAGCTGCCCTCCACTATGCGAATCAACTTAAACAATTGGACA
TAAACACTAGCAAGATGAGCATGGAGGAAGCGTGCTATGGCACTGAAAACCTGGAAGCAATAGACCTCTGCACTAGTGCTGGGTATCCATACAGTGCCCT
TGGTATCAAGAAAAGAGACATTCTCGACCCCATAACCAGGGATGTGTCTAAGATGAAATTCTACATGGATAAATACGGACTAGATCTGCCATACTCTACC
TATGTGAAGGATGAACTTAGATCTCTGGATAAAATCAAGAAAGGAAAGTCACGCCTGATAGAGGCCAGCAGCTTGAATGACTCTGTCTACCTCAGAATGA
CTTTTGGGCACCTTTACGAGGTGTTTCATGCTAACCCTGGTACTGTGACTGGCTCAGCAGTAGGTTGCAACCCAGACGTGTTTTGGAGTAAACTACCGAT
TCTGCTGCCTGGGTCACTCTTTGCCTTTGACTACTCAGGATATGATGCTAGTCTCAGCCCGGTATGGTTCAGGGCTCTAGAAGTTGTGTTACGGAGATT
GGGTATTCAGAGGAGGCCGTGTCCCTAATAGAAGGAATCAACCACACCCACCATGTGTACCGGAATAAAACATACTGTGTACTTGGTGGGATGCCCTCAG
GGTGCTCTGGTACTTCCATCTTCAATTCAATGATCAACAACATCATCATTAGAACCCTTTTGATCAAAACCTTTAAGGGAATAGACCTGGATGAGTTGAA
CATGGTGGCCTATGGGGACGATGTGCTGGCCAGTTACCCTTTTCCTATTGATTGCCTTGAATTGGCTAAGACTGGCAAAGAGTATGGTTTGACCATGACT
CCTGCAGACAAATCACCCTGTTTCAATGAAGTAACATGGGAGAATGCTACCTTCCTGAAGAGAGGGTTCTTGCCAGACCACCAATTTCCATTCTTAATTC
ACCCTACGATGCCCATGAGAGAGATCCATGAGTCCATTCGATGGACTAAGGACGCGCGTAACACCCAGGATCACGTGCGCTCCCTGTGTCTATTGGCATG
GCACAATGGTAAGGATGAATATGAAAAGTTTGTGAGTGCAATTAGATCAGTTCCAGTTGGAAAAGCGTTGGCCATTCCTAACTTTGAGAATCTGAGAAGA
AATTGGCTCGAATTGTTTTAATATTACAGTTTAAAGCTGAACCCCACTAGAAATCTGGTCG

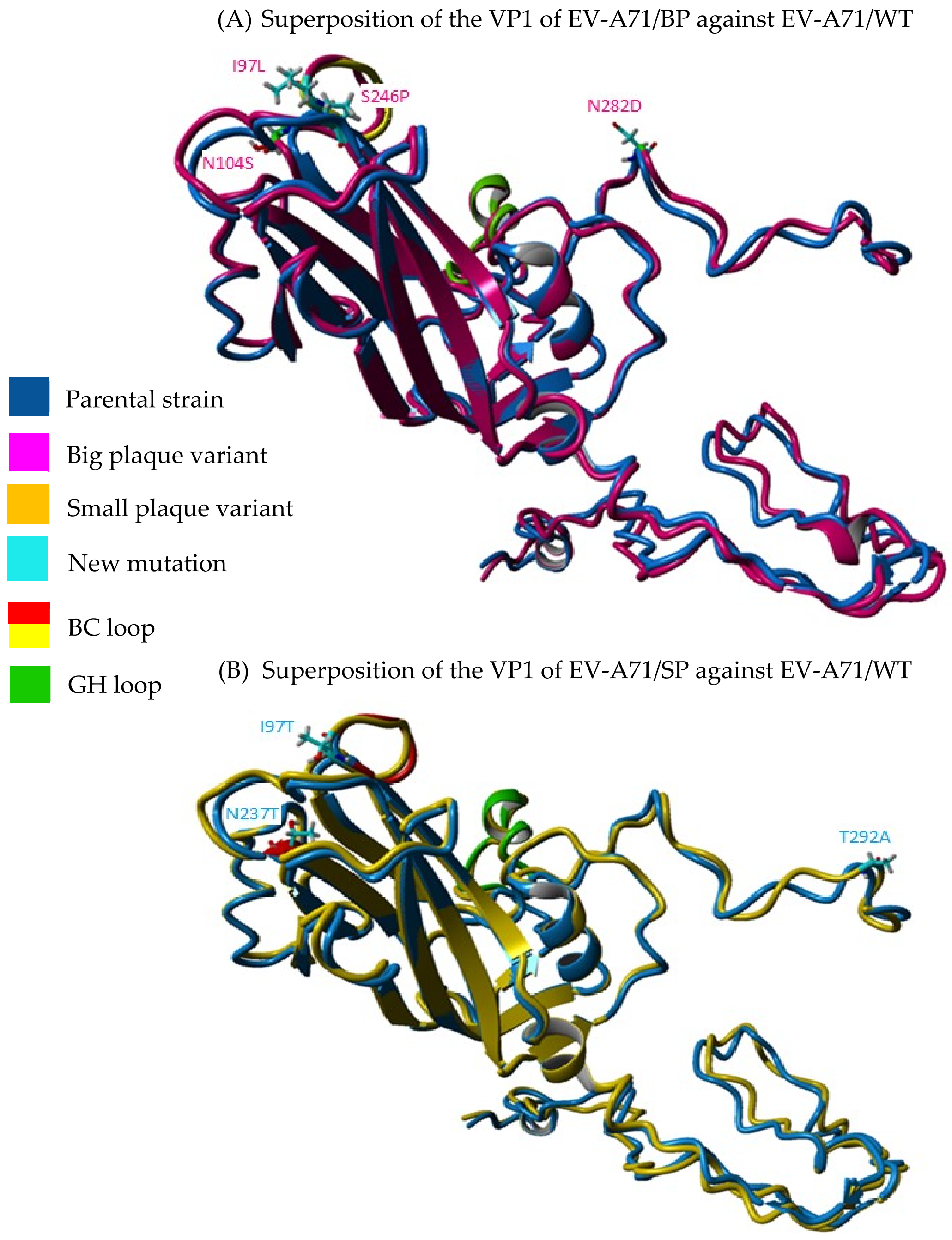


Supplementary Figure S3: The 3D structure of the VP1 protein of the EV-A71/WT superposed against the EV-A71/BP and EV-A71/SP variants. The EV-A71/WT (blue) is showed against the **(A)** EV-A71/BP (magenta) and **(B)** EV-A71/SP variant (yellow) was performed using YASARA. The 4 amino acid substitutions in the EV-A71/BP variant (I97L, N104S, S246P and N282D) and the 3 amino acid substitutions in the EV-A71/SP variant (I97T, N237T and T292A).
